# Flexible polyfluorinated bis-diazirines as molecular adhesives†

**DOI:** 10.1039/d0sc06283a

**Published:** 2021-02-01

**Authors:** Chakravarthi Simhadri, Liting Bi, Mathieu L. Lepage, Mahdi Takaffoli, Zhipeng Pei, Stefania F. Musolino, Abbas S. Milani, Gino A. DiLabio, Jeremy E. Wulff

**Affiliations:** Department of Chemistry, University of Victoria Victoria BC V8W 3V6 Canada wulff@uvic.ca; Materials and Manufacturing Research Institute, University of British Columbia Kelowna BC V1V 1V7 Canada; Department of Chemistry, University of British Columbia Kelowna BC V1V 1V7 Canada

## Abstract

Motivated by a desire to develop flexible covalent adhesives that afford some of the same malleability in the adhesive layer as traditional polymer-based adhesives, we designed and synthesized two flexible, highly fluorinated bis-diazirines. Both molecules are shown to function as effective crosslinkers for polymer materials, and to act as strong adhesives when painted between two polymer objects of low surface energy, prior to thermal activation. Data obtained from lap-shear experiments suggests that greater molecular flexibility is correlated with improved mechanical compliance in the adhesive layer.

## Abstract 2

Flexible, highly fluorinated covalent adhesives are synthesized, and are shown to afford comparable C–H insertion efficiency and adhesion strength relative to a rigid analogue, while providing improved mechanical compliance in the adhesion layer.

## Graphical abstract

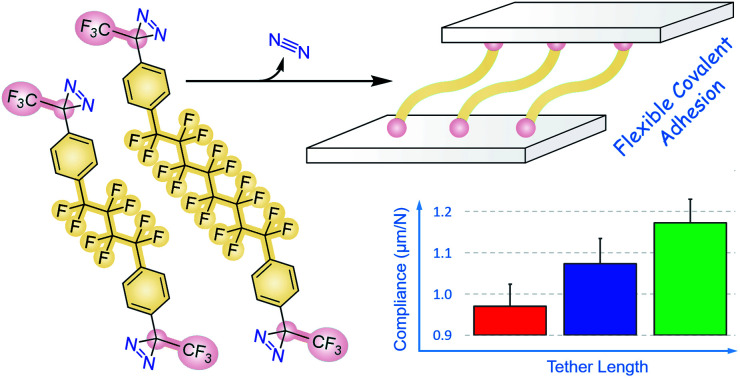

## Introduction

**Fig. 1:**
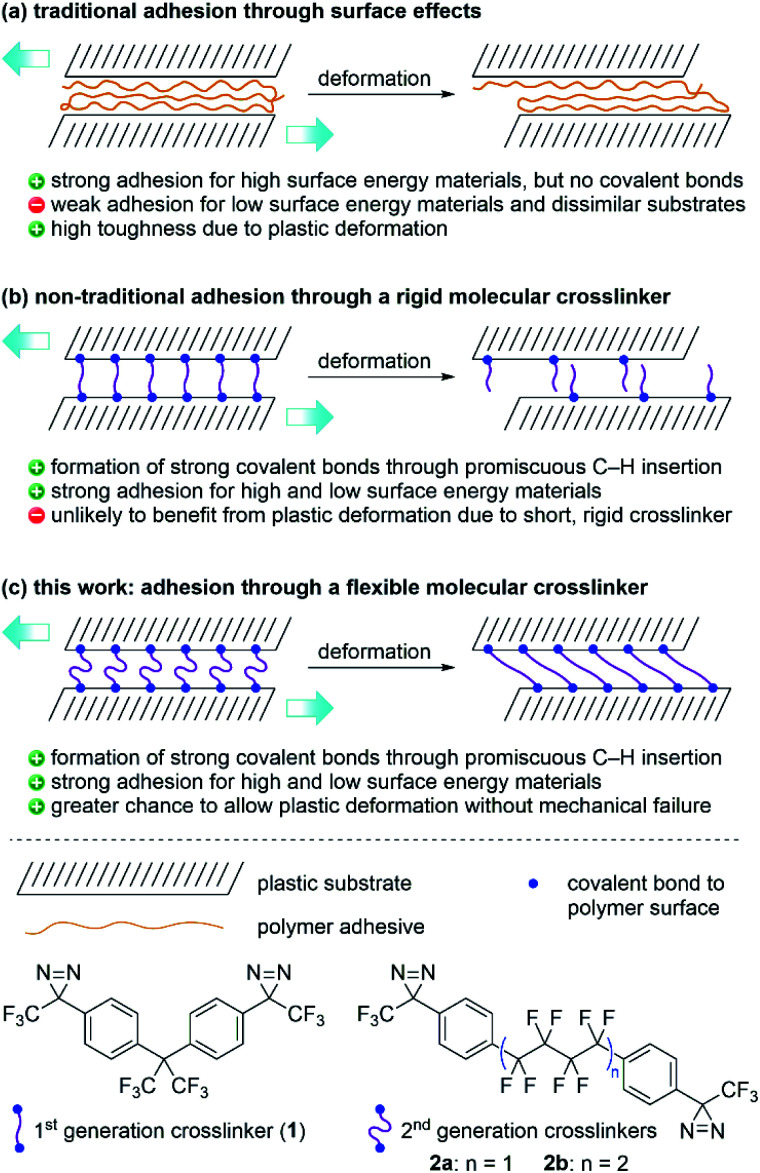
Strategies for adhesion using (a) traditional polymer adhesives, (b) rigid molecular crosslinkers, and (c) flexible molecular crosslinkers, together with structures of the 1^st^ and 2^nd^ generation bis-diazirines.

Traditional adhesives are polymeric materials that work to hold two objects together through physical adsorption effects that depend on surface energy (Fig. 1a).^1–3^ Household adhesives like cyanoacrylates (‘super glues’) that spontaneously polymerize when exposed to moisture, structural adhesives (*e.g.* polyurethanes), pressure-sensitive adhesives used in tape and bandages (*e.g.* polyacrylates), and hobby glue used for paper and wood (*e.g.* polyvinyl acetate or polyvinyl alcohol) all provide adhesion to substrate materials through a combination of van der Waals forces, hydrogen bonds, and mechanical interlocking. These types of polymeric adhesives can provide both strong bonds (especially when the substrate surface contains polar functional groups) and also tough bonds, since the polymer adhesive itself can undergo plastic deformation without experiencing catastrophic mechanical failure.^4,5^ However, adhesion of low-surface energy materials (*e.g.* polyethylene or polypropylene) remains challenging.

An alternative—but less extensively studied—approach to adhesion is to use a reagent that can form strong covalent bonds with the substrate surface. For example, transglutaminase enzymes are used in the food industry as “meat glue” to hold together small cuts of meat, poultry and fish.^6^ This process works by enzymatically linking glutamine residues on one protein surface to lysine residues on an adjacent protein surface. Similarly, polymers functionalized with electrophilic *N*-hydroxysuccinimide (NHS) esters can be used to covalently link amine-functionalized surfaces like human tissue together, providing strong adhesive bonds that can be exploited in wound-healing applications.^7,8^ As is the case with traditional adhesives, however, it is not immediately apparent how one might apply this strategy to low-surface energy polymer substrates, since such materials lack reactive functional groups.

We recently described a bis-diazirine reagent (**1**; Fig. 1b) that can be used as a universal crosslinker for aliphatic polymers.^9^ Compound **1** works by releasing N_2_ upon thermal or photochemical activation to afford reactive carbene species that are capable of undergoing efficient C–H insertion with a wide range of polymer materials. Because bis-diazirine **1** can react twice, it is able to form new connections between polymer strands, resulting in outcomes characteristic of polymer crosslinking: increased average molecular weight, loss of solubility, increased glass transition temperature, loss of melting transition, *etc.*^9–12^ As an added benefit, **1** can be employed as an adhesive for low surface-energy polymers. Simply applying the crosslinker between two pieces of high-density polyethylene (HDPE) prior to thermal curing resulted in strong adhesive bonds of up to *ca.* 2.3 MPa.^9^ However, the rigidity of the linker group in **1** essentially rules out the possibility of any significant plastic deformation at the point of connection between crosslinked polymer strands. This could be a particular problem when the reagent is used as an adhesive since mechanical toughness of an adhesive joint is often thought to rely upon the ability of the adhesant to undergo deformation.^1,4,5^

Mindful of the potential benefits of polymer crosslinkers possessing greater conformational flexibility, we sought to design an analogue of **1** containing a less rigid linker motif. At the same time, however, we wanted to obey the original design rules that had influenced the creation of our first-generation crosslinker. These included: (1) the absence of any labile C–O or C–N bonds that might limit the robustness of crosslinked products; (2) the use of an electron-deficient linker to provide favorable diazirine and carbene electronics, and improved handling under ambient conditions;^13^ and (3) the absence of any aliphatic C–H bonds, to reduce the likelihood of self-reaction. These design constraints led to the selection of **2a** and **2b** (Fig. 1c)—each containing linear perfluoroalkyl linker groups—as our targets for synthesis and materials evaluation. We also considered fluorinating the aromatic rings in **2a**/**b**, but computational investigations (refer to the ESI† for details) indicated that reactions of carbenes with aromatic rings preferentially occur *via* cyclopropanation pathways rather than C–H insertions. Since *in silico* fluorination did not substantively increase the energy barrier for these unwanted reactions, we opted to keep the aryl C–H bonds in our second-generation crosslinkers intact.

## Results and discussion

### Crosslinker synthesis

**Scheme 1:**
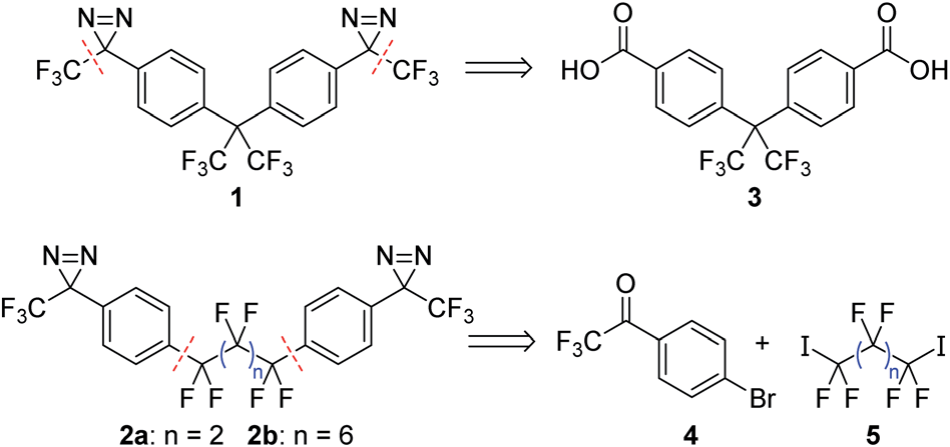
Retrosynthesis for crosslinkers **1** and **2a**/**b**, illustrating the different bond disconnections required for each type of target molecule.

Target compounds **2a** and **2b** were significantly more challenging to access than our 1^st^ generation crosslinker, **1**. Whereas **1** could be rapidly prepared through a ‘building out’ strategy, starting from the commercially available diacid **3** and installing the trifluoromethyl groups *via* the use of the Ruppert–Prakash reagent (TMSCF_3_),^9^ the lack of a readily available core structure mapping onto **2a**/**b** meant that these analogues needed to be synthesized through a ‘building in’ approach where the perfluoroalkyl linker was attached to a suitable aromatic building block (Scheme 1). Additional challenges included a lack of convenient NMR handles (in addition to the obvious dearth of ^1^H nuclei, the large number of inter-coupling ^19^F atoms increased the difficulty of interpreting ^19^F and ^13^C NMR spectra of intermediates) as well as low sensitivities for many of our molecules to mass spectrometric detection (possibly due to difficulties associated with the ionization of polyfluorinated compounds). These spectroscopic challenges alone would not have been a significant impediment, but many of the intermediates we studied en route to **2a** and **2b** also turned out to be *fluxional*, since the increased electrophilicity of dependent functional groups frequently led to the formation of interconverting hydrates and oligomers that further complicated NMR analysis (*vide infra*).

**Scheme 2:**
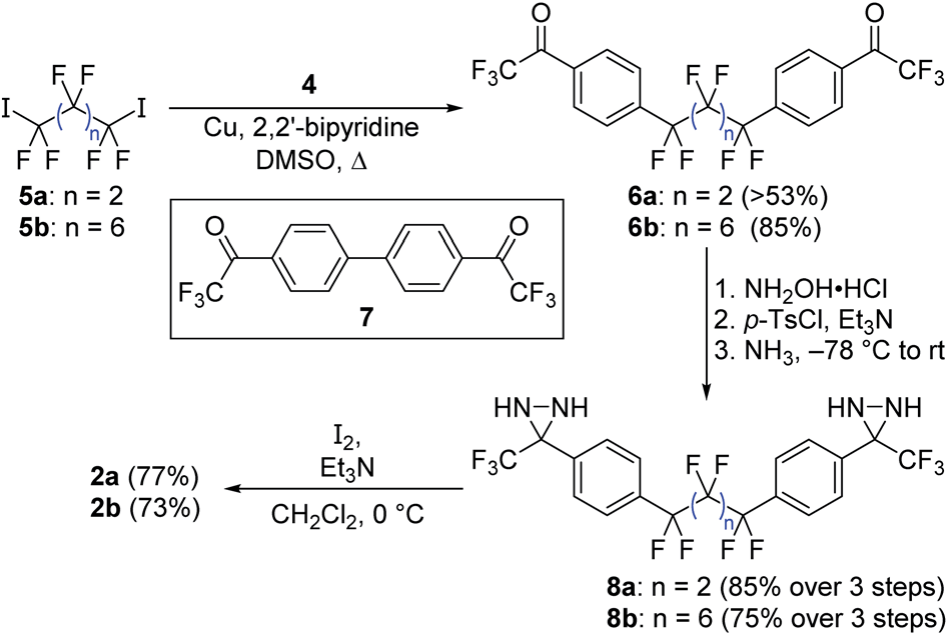
Synthesis of target compounds **2a** and **2b** through a copper-promoted coupling of a perfluoroalkyl iodide to an aryl bromide incorporating a trifluoromethyl ketone.

Following the exploration of several unsuccessful routes, we found that aryl bromide **4** (already functionalized with a trifluoromethylketone moiety that we expected to be able to convert into the corresponding diazirine) could be coupled efficiently to perfluoroalkyl diiodides **5a** and **5b** using copper catalysis (Scheme 2).^14^ This method avoids the formation of strongly anionic centers that could trigger unwanted elimination reactions from the perfluoroalkyl scaffold, and forges the two key C–C bonds necessary to complete the synthesis of the target compound. While the attachment of monovalent perfluoroalkyl sidechains to aromatic molecules in this manner is well known,^15–18^ and has often been exploited to design new fluorous-phase reagents,^19–25^ the use of divalent perfluoroalkyl reagents to establish fluorinated linkers between two aromatic rings is less common,^26–28^ perhaps due to competing metallacycle formation.^29^

The reaction of **4** with 1,4-diiodoperfluorobutane (**5a**) on 5 gram scale provided a crude product that was triturated with dichloromethane to afford 53% of pure **6a** as a white solid. Additional desired product was observed in the supernatant, but recovery was complicated by the presence of homodimer **7** (which could not be readily separated chromatographically), and by the fact that both **6a** and **7** readily form hydrates in the presence of air. To maximize the amount of material available for later studies, the mixture of **6a** and **7** was carried forward separately from the pure **6a** that was available by trituration.

Oxime formation, tosylation, and ammonia addition were carried out using similar protocols to those previously employed for crosslinker **1**,^9^ which in turn were based on literature precedent.^11^ The tendency for both **6a** and **7** to form hydrates did not restrict the ability of the substrates to form oximes. A mixture of hydrated and non-hydrated forms of **6a** (with or without **7**) could be subjected directly to standard oxime formation conditions, leading to the production of the desired intermediate in near-quantitative yield. As with the parent ketone, purification and characterization of the oxime intermediate was challenging (this time due to the presence of *E*,*E*, *Z*,*Z* and *E*,*Z* isomers), but once again the crude mixture could be carried onto the next step without further purification.

Following the three-step protocol described above, 2.7 grams (85%) of pure diaziridine **8a** were prepared from 3.0 grams of triturated **6a**, while a further 0.7 grams of pure **8a** was obtained from supernatant-derived material that was contaminated with **7** (the bis-diaziridine derivative of **7** was made at the same time, but the two species could finally be separated chromatographically at the diaziridine stage).

Synthesis of the longer crosslinker was made somewhat easier by the fact that the ketone intermediate (**6b**) could be separated from **7** using column chromatography in good yield (85%). This was similarly converted to the corresponding bis-diaziridine (**8b**). Both diaziridines were then oxidized to the desired bis-diazirine target compounds (**2a** and **2b**) without incident.

### Evaluation of crosslinking activity

**Scheme 3:**
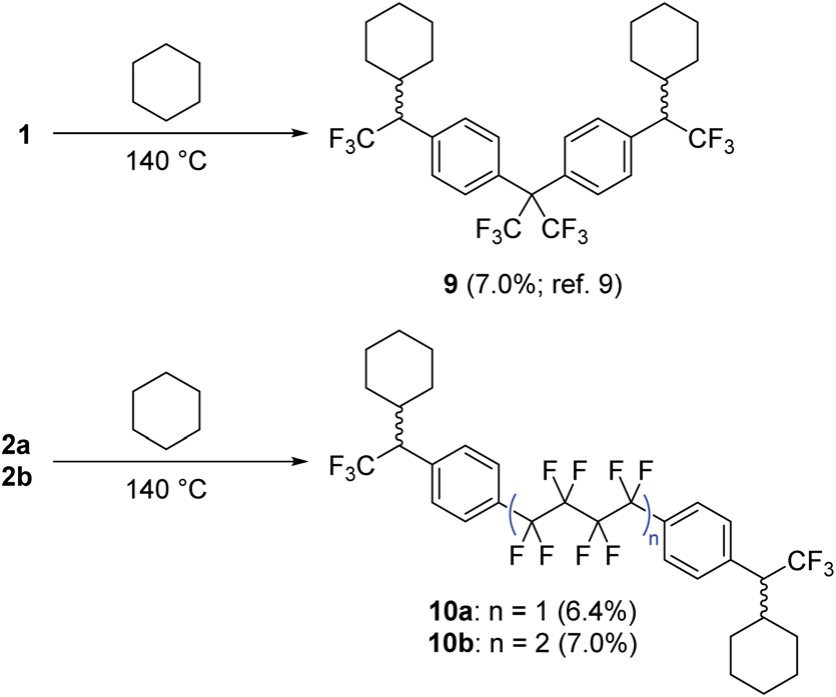
Comparison of cyclohexane crosslinking efficacy for **1**, **2a** and **2b**. Adducts **9**, **10a** and **10b** were fully characterized following each experiment. Refer to ref. 9 for spectroscopic details for compound **9**, and the ESI† for spectroscopic details for **10a** and **10b**.

Following confirmation by Yoshida analysis^9,30^ that **2a** and **2b** are not likely to present a risk of explosion (see Fig. S52 in the ESI†), we sought to characterize the two new compounds as molecular crosslinkers. We previously employed cyclohexane as a molecular model of polyethylene, and showed that **1** could crosslink this challenging substrate upon thermal activation.^9^ The isolated yield of the pure bis-cyclohexane adduct (**9**, Scheme 3) was 7.0% when the reaction was conducted at 140 °C, a number that we regarded as the lower limit of crosslinking efficacy since it does not include alternative crosslinked structures wherein the reagent oligomerizes prior to crosslinking, or structures wherein >1 C–H insertion occurs to the same cyclohexane unit. Repeating this experiment with **2a** and **2b**, we found that both compounds performed similarly to **1**, permitting the isolation of the purified cyclohexane adducts **10a** and **10b** in 6.4 and 7.0% yield, respectively (Scheme 3).

**Fig. 2:**
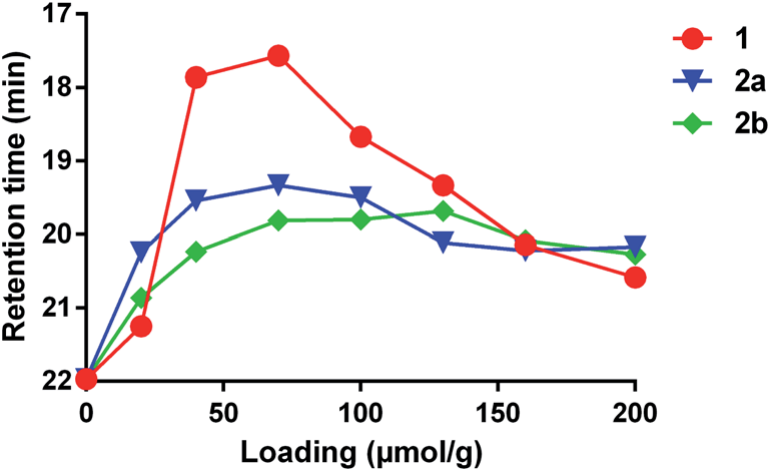
Comparison of PDMS crosslinking efficacy for **1**, **2a** and **2b**. GPC data (using viscosity detection) was recorded for low-viscosity PDMS (25 cSt) containing varying amounts of each crosslinker following thermal activation at 110 °C for 16 h (see ESI† for full details). The retention time at which the largest viscosity signal was observed for each experiment was then plotted against crosslinker concentration. The resulting curves confirm that all three reagents are capable of crosslinking PDMS.

We also compared the effectiveness of all three compounds in crosslinking low-molecular weight polydimethylsiloxane (PDMS). As expected, each compound was capable of increasing the average mass of the polymer, as evidenced by a shift of the peak to lower retention times in a GPC measurement (Fig. 2). The lowest retention times (*i.e.* highest average molecular weights of soluble polymer) were observed to occur at *ca.* 70 μmol crosslinker per gram of polymer for both **1** and **2a**; addition of further crosslinker beyond this concentration led to a shift of the main peak back to higher retention times—probably due to the fact that highly crosslinked PDMS is no longer soluble and is therefore lost when the sample is filtered.^9^ Crosslinker **2b** behaved similarly, but did not show as much of a dramatic change in the retention time as the concentration of reagent was increased beyond *ca.* 70 μmol per gram. Together with the crosslinking of cyclohexane described above, these data confirm that **1** and **2a**/**b** have similar crosslinking properties.

### Adhesion testing and mechanical compliance

**Fig. 3:**
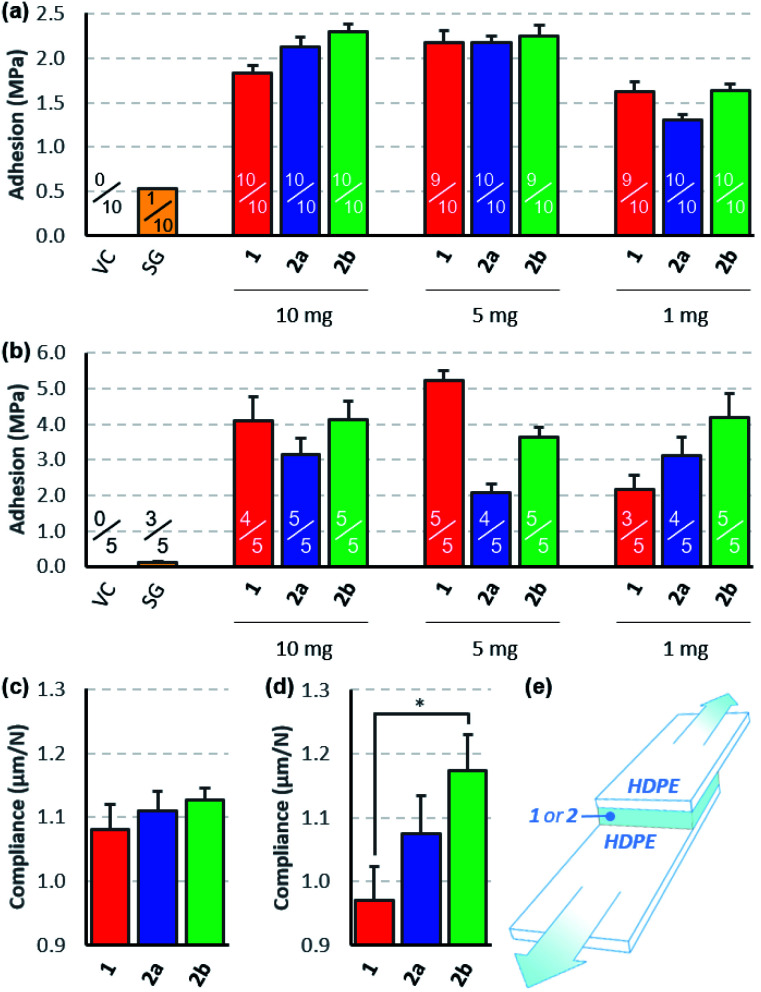
Lap-shear data for crosslinkers **1**, **2a** and **2b**. (a) Adhesion strength data collected using new HDPE bars, confirming bonding for all three crosslinkers but not for a traditional cyanoacrylate adhesive used as a control. Fractions indicate the number of samples that survived clamping into the instrument. (b) Adhesion strength data collected using >6 month old HDPE bars, showing improved bonding for all three crosslinkers, but not for the cyanoacrylate control. (c) The ratio of extension to force for lap-shear samples prepared from new HDPE bars treated with 1 mg of each crosslinker. (d) The ratio of extension to force for lap-shear samples prepared from aged HDPE bars treated with 1 mg of each crosslinker. (e) A schematic of the lap-shear samples used for the experiment. The overlap region is 2.54 cm wide × 1.27 cm long. VC = vehicle control. SG = super glue (≥10 mg). * indicates *p* < 0.05. Error bars indicate standard error in all cases.

To compare the effectiveness of the three bis-diazirines as adhesives for low surface energy polymers, we prepared lap-shear samples of high-density polyethylene (HDPE), using either 10, 5, or 1 mg of **1**, **2a** or **2b** in the 2.54 cm × 1.27 cm overlap region.^31^ Samples were cured in an oven set to 115 °C (the temperature was deliberately set near the beginning of the diazirine-activation curve observed from differential scanning calorimetry experiments (see Fig. S50 and S51†) in order to avoid unwanted softening of the HDPE substrate). Following several days storage at room temperature, the samples were pulled apart at 3 mm min^−1^ until failure was observed (Fig. 3e).^32^ To evaluate the role of polyethylene surface chemistry on adhesion, samples were made using both freshly received HDPE (Fig. 3a) and HDPE bars that had been stored in our lab for >6 months (Fig. 3b).

All three crosslinkers performed far better than the cyanoacrylate ‘super glue’ used as a control, although the measured adhesion strength differed depending on the source of HDPE used for the experiments. When newly purchased HDPE samples were used, we observed very similar levels of adhesion to those reported previously^9^ and found that all three crosslinkers afforded comparable levels of bonding. With samples made from older HDPE bars, we found significantly increased adhesion strength (up to >5 MPa) and greater variability in performance between the crosslinkers. It is well known that polyethylene surfaces are prone to oxidation upon standing, and that this surface oxidation contributes to increased adhesion,^33^ although evidently the surfaces were not sufficiently oxidized to result in successful bonding using the cyanoacrylate adhesive. We speculate that the presence of small amounts of hydroxyl or carboxylic acid groups on the surface of the aged HDPE led to an increase in the efficiency of covalent bond formation, since carbenes are known to insert more effectively into O–H bonds than C–H bonds.^34–36^

Although all three crosslinkers gave broadly similar levels of adhesion in most experiments (consistent with the similar yields of cyclohexane crosslinking described in Scheme 3), there were some differences that are worth noting. First, we observed that our first-generation crosslinker, **1**, provided optimal adhesion when applied at 5 mg per lap-shear sample (1.6 mg cm^−2^). This is consistent with our earlier report showing that 10 mg of **1** provided superior adhesion to 20 mg of **1**,^9^ and probably results from the fact that at very high loadings, crosslinker oligomerization outcompetes C–H insertion. At very low loadings, of course, there is insufficient crosslinker present to bond the two HDPE samples. Second, we found that the adhesive force provided by **2a** and **2b** was less sensitive to the amount of crosslinker used (at least within the range being tested here), and that **2b** always performed slightly better than **2a**, regardless of the amount of crosslinker used or the age of the HDPE used to prepare the samples. This is particularly notable given that equal weights of **2a** and **2b** were applied in the lap-shear experiment. Since **2b** has a molecular weight that is 35% larger than that of **2a**, this means that **2b** was a particularly good performer on a molar basis. Combining the trends noted above, we found that when we minimized the loading of crosslinker and employed the more accommodating of our available HDPE surfaces (Fig. 3b), **2a** and **2b** could provide superior adhesion to **1**—but this is clearly a rather specialized collection of parameters and should not be taken as an indication that our second generation crosslinkers are superior from the perspective of absolute adhesion.

Indeed, outside of subtle differences due to different packing preferences of the three molecules in the lap joint, ultimate tensile strength should be roughly constant for all three crosslinkers, since this parameter will mostly depend on the efficiency of C–H insertion, which should be almost identical for the three electronically similar molecules. On the other hand, if the different conformational flexibility of the three crosslinkers allows for greater deformation in the lap-shear sample prior to breakage (as hypothesized in Fig. 1c), we should see this reflected in a measure of mechanical compliance (or ‘stretchiness’) obtained by dividing the maximum extension of each sample prior to lap-shear failure (in microns) by the maximum force (in Newtons).^37^ Recognizing that at higher crosslinker loadings (10 mg or 5 mg per sample) crosslinker self-reaction and polymerization would complicate our analysis, we calculated mechanical compliance for both sets of lap-shear samples made with 1 mg crosslinker (0.3 mg cm^−2^).

This analysis revealed that for both new (Fig. 3c) and old (Fig. 3d) HDPE samples, compliance increased with increasing flexibility of the crosslinker used to prepare the sample. Importantly, this trend was maintained even in a case where the more rigid crosslinker (**1**) provided a superior adhesive force (*i.e.* compare the data in Fig. 3c with the data on the right-hand side of Fig. 3a). Of equal importance, the data obtained using the older HDPE samples (where the presence of trace O–H groups presumably further supports bonding to the surface over crosslinker self-reaction) showed a statistically significant difference in compliance (*p* < 0.05) as one moves from the least flexible to the most flexible crosslinker (Fig. 3d).

Additional evidence in favor of the hypothesis that more flexibility within the crosslinker structure can reduce the incidence of brittle fracture comes from the numbers of samples that survived the lap-shear testing protocol (indicated as fractions in Fig. 3a and b). This involves mounting the bonded samples between two clamps; if the sample is mounted imperfectly by the operator, the torque that results from tightening the clamps can snap the samples before the lap-shear experiment even begins. Adhesives that allow for greater plastic deformation within the adhesion layer should perform better in this mounting protocol, and indeed our data indicate that while 5 of 45 samples treated with crosslinker **1** snapped during loading, only 2 of 45 samples treated with **2a** suffered the same fate, and a mere 1 of the 45 samples treated with **2b** were lost.

Having thus compared the adhesive properties of **1**, **2a** and **2b** for HDPE samples, we briefly surveyed their utility for the adhesion of other nonpolar (Fig. S58†) and polar (Fig. S59†) polymers. We found that all three crosslinkers provided effective adhesion for polypropylene–polypropylene bonding (>2.5 MPa adhesion strength when 5 mg of **2b** was used), and found that all three compounds could likewise be used to bond dissimilar polymer materials. HDPE–polypropylene samples and ultra-high molecular weight polyethylene (UHMWPE)–polypropylene samples both exhibited strong bonds (>2.5 MPa) when treated with 5 mg of **2b**. Turning to polar polymer samples (poly(methyl methacrylate) or polycarbonate) we were surprised to find that the flexible crosslinkers **2a** and **2b** dramatically outperformed first-generation crosslinker **1**. Compound **2a** was particularly efficacious in these experiments. In fact, the use of 5 mg of **2a** provided a sufficiently strong bond for poly(methyl methacrylate) adhesion that all three tested lap-shear samples exhibited stock break failure, where the poly(methyl methacrylate) substrate broke (at 4.3 MPa, or 1400 N; see Fig. S59†) before rupture of the bond was observed.

## Conclusions

Taken together, our results indicate that crosslinkers **2a** and **2b** provided comparable (and in some cases superior) levels of adhesion to our first-generation bis-diazirine, **1**, while allowing for increased deformation within the joint. These data provide the most compelling evidence to date in favour of the value of flexible, highly fluorinated molecular adhesives, and suggest that flexible covalent adhesives—much like the flexible butyl- and octyl-cyanoacrylates in development for surgical applications^38^—could be preferred over less flexible analogues.

## Author contributions

C. S. synthesized crosslinkers **2a** and **2b**, performed the initial cyclohexane crosslinking experiments, and characterized C–H insertion products **10a** and **10b** with purification assistance from S. M. M. L. carried out the PDMS crosslinking measurements and analyzed the data. DSC experiments were done by C. S. and L. B. Lap-shear samples were prepared by L. B. and C. S., and adhesion strength was measured by M. T. and A. M. Computational experiments were carried out by Z. P. and G. D. The project was conceived by J. W., who also wrote the manuscript with input from all authors.

## Conflicts of interest

C. S., M. L. and J. W. are coauthors on U.S. Patent Application 62/839,062, which claims the use of crosslinkers described in this work. The authors declare no further conflict of interest.

## Supplementary Material

SC-012-D0SC06283A-s001
