# Supplementary material for: Flexible polyfluorinated bis-diazirines as molecular adhesives
Source: Chem Sci. 2021 Feb 1;12(11):4147–53. doi: 10.1039/d0sc06283a (PMC8179497; doi:10.1039/d0sc06283a)
Supplement: SC-012-D0SC06283A-s001 [file SC-012-D0SC06283A-s001.pdf]

Electronic Supplementary Material (ESI) for Chemical Science.
This journal is © The Royal Society of Chemistry 2021

Electronic Supplementary Information for:

Flexible Polyfluorinated Bis-Diazirines as Molecular Adhesives

Chakravarthi Simhadri, Liting Bi, Mathieu L. Lepage, Mahdi Takaffoli, Zhipeng Pei, Stefania F. Musolino, Abbas S. Milani, Gino A. DiLabio, and Jeremy E. Wulff *

Table of Contents **page**

Experimental Procedures S2
1. General considerations S2
2. Synthesis of crosslinkers S2
    2.1 Synthesis of bis-ketone **6a** S2
    2.2 Synthesis of bis-oxime **11a** S5
    2.3 Synthesis of tosyl oxime **12a** S7
    2.4 Synthesis of bis-diaziridine **8a** S10
    2.5 Synthesis of bis-diazirine **2a** S14
    2.6 Synthesis of bis-diazirine **14** S16
    2.7 Synthesis of bis-ketone **6b** S18
    2.8 Synthesis of bis-oxime **11b** S20
    2.9 Synthesis of tosyl oxime **12b** S22
    2.10 Synthesis of bis-diaziridine **8b** S24
    2.11 Synthesis of bis-diazirine **2b** S26
3. Crosslinking of cyclohexane, as a molecular model for polyethylene S28
    3.1 General procedure for the thermal crosslinking of cyclohexane S28
    3.2 Cyclohexane crosslinking with **2a** to afford **10a** S28
    3.3 Cyclohexane crosslinking with **2b** to afford **10b** S32
4. Assessment of Thermal Properties S36
    4.1 Differential scanning calorimetry (DSC) S36
    4.2 Assessment of explosivity S37
5. Crosslinking of PDMS S38
    5.1 Experimental procedure for comparison of crosslinking effects of **1**, **2a** and S38
6. Adhesion Testing S43
    6.1 Preparation of adhered HDPE–HDPE samples S43
    6.2 Preparation of adhered HDPE–PP and UHMWPE–PP samples S43
    6.3 Preparation of adhered PMMA–PMMA, PP–PP and PC–PC samples S43
    6.4 Lap-shear test S43
    6.5 Analysis of lap-shear samples following breakage S47
Supplemental Results and Discussion S49
1. In Silico Evaluation of Carbene Additions to Arenes vs. Perfluoroarenes S49
References S68

Experimental Procedures

1. General considerations

All reagents used for the synthesis of target compounds were purchased from MilliporeSigma except 4-bromo-2,2,2-trifluoroacetophenone and hexadecafluoro-1,8-diiodooctane, which were purchased from Matrix scientific and Manchester Organics, respectively. All commercial materials were used as received. Dry dichloromethane (DCM) was obtained by passing DCM over alumina in a commercial solvent purification system. Anhydrous dimethyl sulfoxide (DMSO) was prepared by further drying commercial anhydrous DMSO over activated 4 Å molecular sieves for at least 24 hours before use. Anhydrous tetrahydrofuran (THF) was obtained by freshly distilling THF from sodium and benzophenone. Reagent grade solvents were used for extractions and washings. Spectranalyzed™ pentane was used for the purification of bis-diazirine compounds (**2a**, **2b** and **14**) and crosslinked products (**10a** and **10b**).

NMR spectra were recorded at ambient temperature using either a Bruker AVANCE 300 (300.27 MHz for ^{1}H, 282.54 MHz for ^{19}F, 75.5 MHz for ^{13}C) or Bruker AVANCE Neo 500 (500.27 MHz for ^{1}H, 470.72 MHz for ^{19}F, 125 MHz for ^{13}C) spectrometer. Chemical shifts were reported in parts per million (ppm) and were calibrated to the central peak of residual NMR solvent (central peak of acetone-d6: ^{1}H NMR δ = 2.05 ppm, ^{13}C NMR δ = 29.84 ppm; chloroform-d: ^{1}H NMR δ = 7.26 ppm, ^{13}C NMR δ = 77.16 ppm, dichloromethane-d2: ^{1}H NMR δ = 5.32 ppm, ^{13}C NMR δ = 53.84 ppm). ^{13}C spectra and ^{19}F spectra were ^{1}H decoupled unless otherwise noted. Data is reported as follows: chemical shift (multiplicity (s = singlet, d = doublet, t = triplet, q = quartet, p = pentet, dt = doublet of triplet, tt = triplet of triplet, td = triplet of doublet, br-s = broad singlet, m = multiplet), coupling constant in Hz, integration). Spectral assignments were made for compounds that gave well-resolved spectra in HSQC and HMBC experiments. Reaction progress was monitored by either thin-layer chromatography on pre-coated aluminium-backed silica gel 60 F254 plates followed UV visualization or by ^{1}H/^{19}F NMR spectroscopy. IR spectra were recorded using a Perkin-Elmer ATR spectrometer. High resolution mass spectra (HRMS) were recorded on a Thermo Scientific Orbitrap Exactive Plus spectrometer using electrospray ionization experiments for all compounds unless otherwise noted. HRMS spectra of bis-diazirine compounds (**2a**, **2b** and **14**) and crosslinked products (**10a** and **10b**) were acquired using field desorption (FD) ionization on a JEOL AccuTOF spectrometer.

2. Synthesis of crosslinkers

*2.1 Synthesis of bis-ketone **6a***

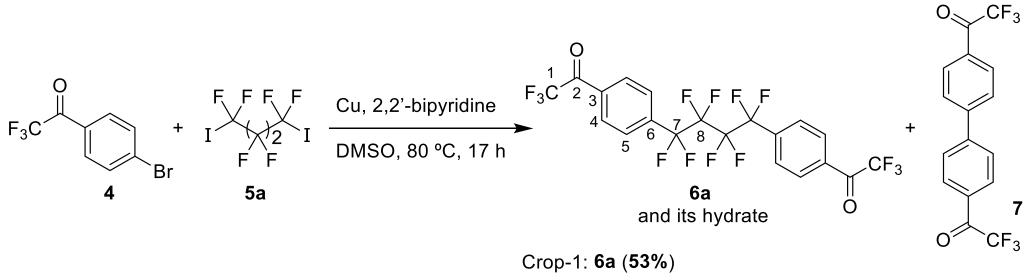

Crop-1: **6a** (**53%**)
Crop-2: mixture of **6a** + homo-coupled bisketone (**7**) + other impurities

Synthesis of **6a** was performed using a modification of a literature procedure.[1] In a two-neck 250 mL round bottom flask equipped with a magnetic stir bar and a condenser, dry DMSO (13.53 mL) was added to 4'-bromo-2,2,2-trifluoroacetophenone (**4**; 5.50 g, 21.74 mmol, 1 equiv.) under argon. To this stirring solution, octafluoro-1,4-diiodobutane (**5a**; 1.95 mL, 10.65 mmol, 0.49 equiv.), copper powder (12.71 g, 199.98 mmol, 9.2 equiv.), and 2,2'-bipyridine (0.68 g, 4.35 mmol, 0.2 equiv.) were added

simultaneously at room temperature. The reaction flask was evacuated and backfilled with argon and then heated to 80 °C. After 17 hours of stirring at 80 °C, the reaction mixture was cooled to room temperature, diluted with EtOAc (100 mL) followed by water (20 mL), and stirred for 10 minutes. The resulting slurry was filtered through a celite pad and the residue was washed with water and EtOAc. The filtrate was transferred into a separatory funnel and the layers were separated. The aqueous layer was extracted with EtOAc (3 × 50 mL). The organic layers were combined, washed subsequently with water (1 × 30 mL) and brine (1 × 30 mL), dried over Na_2SO_4, and concentrated in vacuo. The resulting green residue was triturated with a minimal amount of DCM and filtered, after which the solid was washed with DCM to afford **6a** (3.1 g, 53%) as a white solid (crop-1). The filtrate was concentrated to give 6.21 g of a green oil (crop-2). This crude mixture contained the desired product (**6a**), along with the homo coupling product (**7**), and other impurities. Purification attempts on this mixture were unsuccessful. Hence, the next steps were carried out separately with crop-1 and crop-2.

Triflurometyl ketones **6a** and **7** readily form hydrates. In order to simplify the NMR spectra for the product, NMR samples were prepared in acetone-d6 left to incubate for at least 6–8 hours before acquisition to allow hydration to proceed to completion. ^{1}H NMR (500 MHz, acetone-d6) δ 8.32 (d, *J* = 8.3 Hz, 0.2H, H-4), 8.00 (d, *J* = 8.3 Hz, 0.2H, H-5), 7.95 (d, *J* = 8.3 Hz, 3.8H, bis-ketone hydrate H-4), 7.73 (d, *J* = 8.3 Hz, 3.8H bis-ketone hydrate H-5), 6.83 (s, 4H, bis-ketone hydrate OHs). ^{19}F NMR (471 MHz, acetone-d6) δ –72.69 (0.26F, CF_3), –84.63 (5.74F, bis-ketone hydrate CF_3), –110.94 (t, *J* = 13.7 Hz, 3.69F), –111.79 (t, *J* = 14.1 Hz, 0.26F), –121.86 (t, *J* = 14.1 Hz, 4F). ^{13}C NMR (125 MHz, acetone-d6) δ 143.58 (bis-ketone hydrate C-3), 131.16 (q, $^{2}J_{C\text{-}F}$ = 2.1 Hz, bis-ketone hydrate C-6), 129.03 (bis-ketone hydrate C-4), 127.23 (t, $^{3}J_{C\text{-}F}$ = 6.5 Hz, bis-ketone hydrate C-5), 124.3 (q, $^{1}J_{C\text{-}F}$ = 289 Hz, bis-ketone hydrate C-1), 119.69–109.32 (m, bis-ketone hydrate C-7 and C-8), 93.80 (q, $^{2}J_{C\text{-}F}$ = 31.9 Hz, bis-ketone hydrate C-2). IR (ATR) υ (cm^{-1}): 3148, 1732, 1415, 1284, 1183, 1155, 1102, 1104, 849. HRMS (ESI+) m/z [M+H] calculated for $C_{20}H_9F_{14}O_2^{+}$: 547. 0374, found: 547.0383.

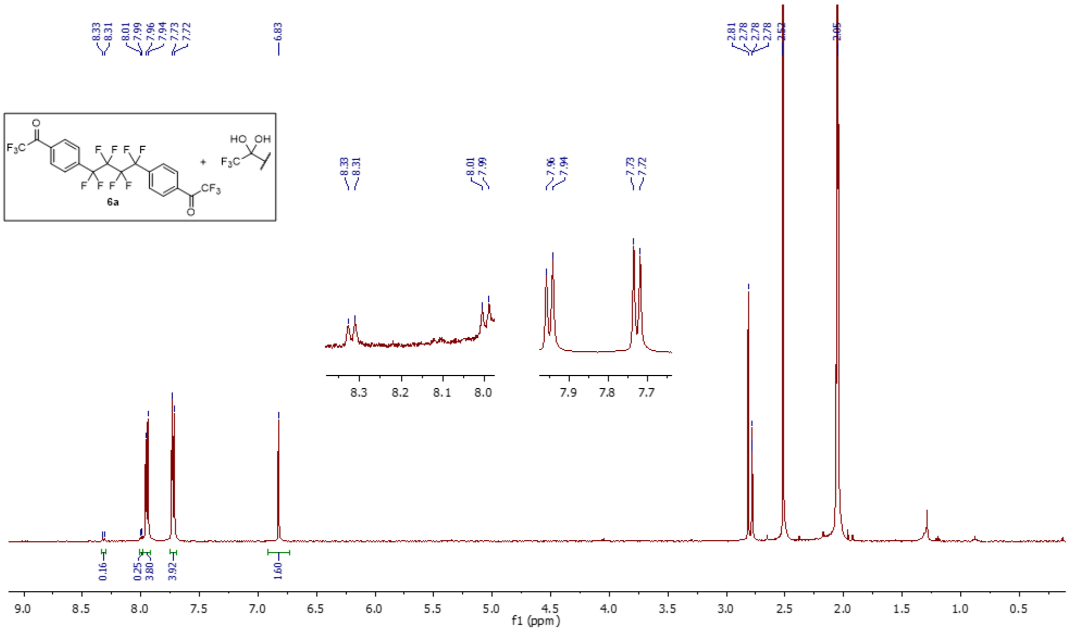

Figure S1. ^{1}H NMR spectrum of **6a** and its hydrate in acetone-d6 at 500 MHz. The hydrate is the dominant species in the equilibrating mixture.

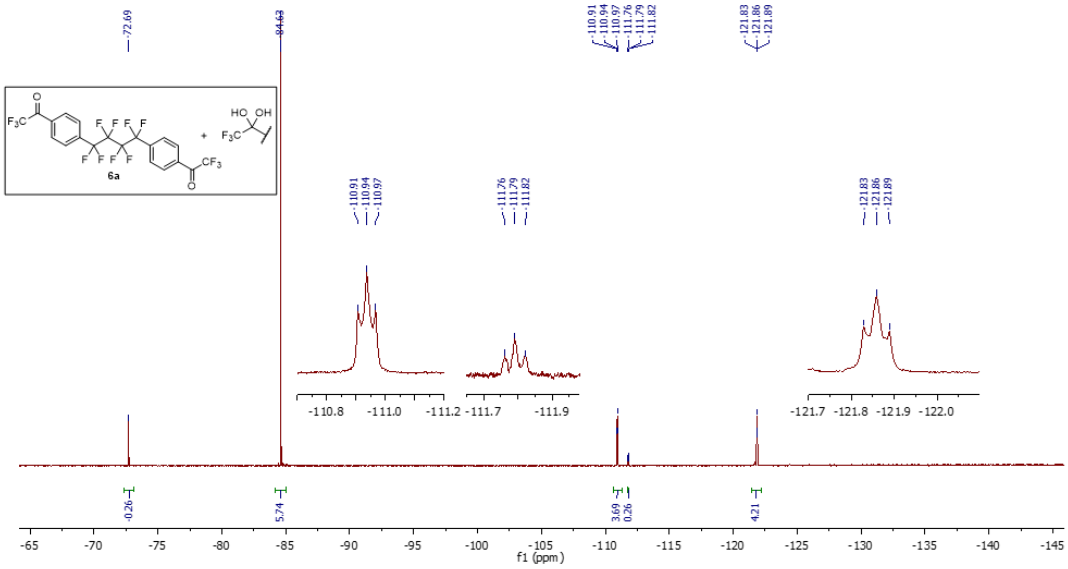

Figure S2. ^{19}F NMR spectrum of **6a** and its hydrate in acetone-d6 at 471 MHz. The hydrate is the dominant species in the equilibrating mixture.

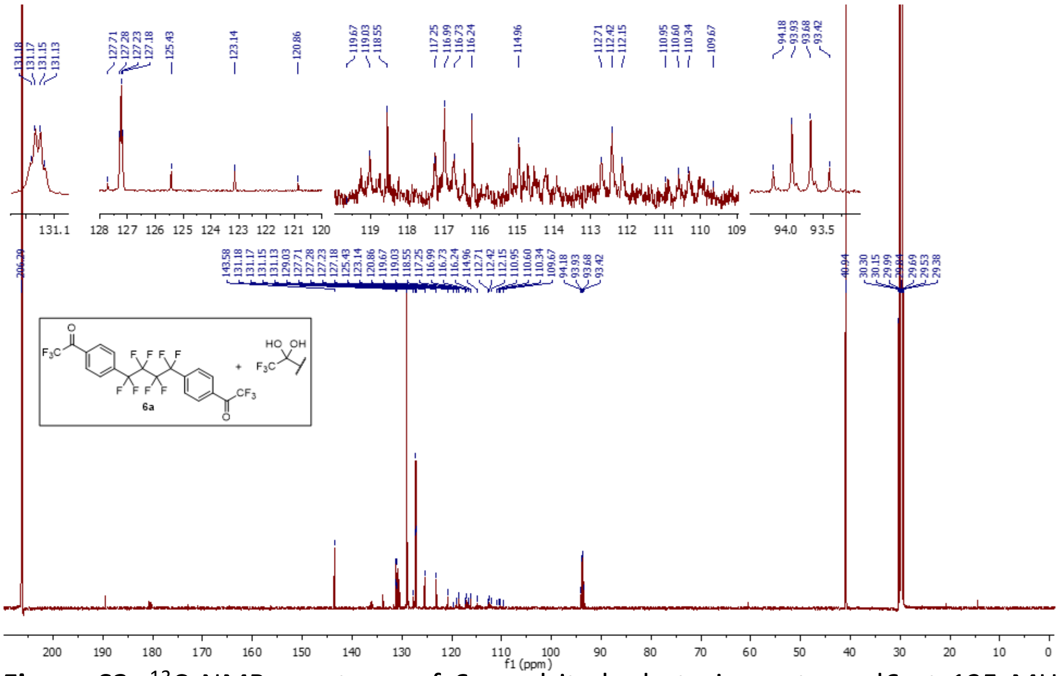

Figure S3. ^{13}C NMR spectrum of **6a** and its hydrate in acetone-d6 at 125 MHz. The hydrate is the dominant species in the equilibrating mixture.

2.2 Synthesis of bis-oxime ***11a***

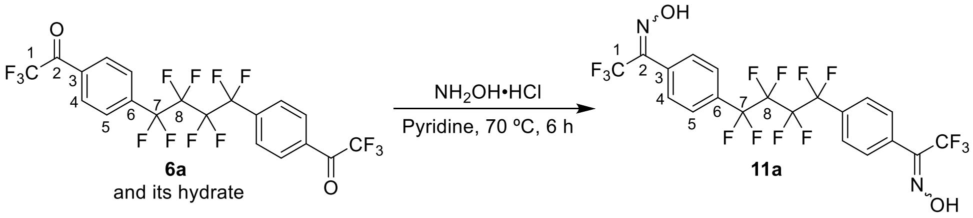

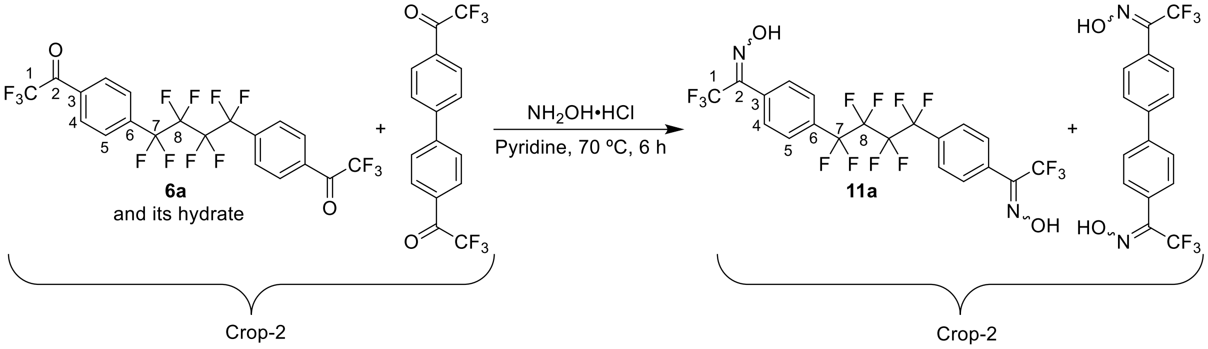

With crop-1 (pure ketone/ketone hydrate): To a stirring solution of **6a** (3.00 g, 5.49 mmol, 1 equiv.) in pyridine (30 mL), hydroxylamine hydrochloride (2.29 g, 32.95 mmol, 6 equiv.) was added at room temperature under argon. The reaction mixture was heated to 70 °C. After 6 hours of stirring at 70 °C, the reaction mixture was allowed to cool to room temperature, and was then concentrated in vacuo to remove pyridine. The resulting residue was partitioned between EtOAc (100 mL) and water (30 mL). The aqueous layer was further extracted with EtOAc (2 × 50 mL). The organic layers were combined and subsequently washed with water (1 × 30 mL), 1N HCl (3 × 20), water (2 × 20), and brine (1 × 30 mL). The organic layer was dried over Na_2SO_4 and concentrated to afford the desired product (3.5 g, 110% crude) as a white solid, which was subjected to the next step without purification. The product is mixture of syn- and anti- isomers. ^{1}H NMR (500 MHz, acetone-d6) δ 12.47 (broad-s, oxime-OH), 12.07 (broad-s, oxime-OH), 8.16–7.25 (m, 8H). ^{19}F NMR (471 MHz, acetone-d6) δ –63.04 (s, 2.81F), –66.79 (d, J = 2.0 Hz, 3.10F), −111.20 (p, J = 15 Hz, 3.9F), −121.13 – −123.06 (m, 3.88F). ^{13}C NMR (125 MHz, acetone-d6) δ 145.92 (q, $^2J_{C\text{-}F}$ = 32.3 Hz, C-2) and 145.77 (q, $^2J_{C\text{-}F}$ = 29.7 Hz, C-2'), 135.55, 131.80, 130.33, 129.74, 127.99 (q, J = 6.9 Hz), 121.92 (q, $^1J_{C\text{-}F}$ = 273.1 Hz, C-1) and 119.73 (q, $^1J_{C\text{-}F}$ = 282.1 Hz, C-1'), 118.88 (tt, $^1J_{C\text{-}F}$ = 256.7 Hz, $^2J_{C\text{-}F}$ = 29.3 Hz) and 118.88 (tt, J = 256.0 Hz, J = 29.7 Hz), 115.00–110.10 (m). IR (ATR) υ (cm^{-1}): 3292, 1410, 1283, 1175, 1137, 1110, 959, 852. HRMS (ESI–) m/z [M–H] calculated for $C_{20}H_9F_{14}N_2O_2^-$: 575.0446, found: 575.0427.

With crop-2: Reaction and work up were carried out similarly to the protocol described above using 6.2 g of the ketone mixture described above, together with 60 mL pyridine and 4.66 g hydroxylamine hydrochloride. After workup, 5.71 g crude product was obtained as a mixture of oximes, and was carried forward to the next step without any purification.

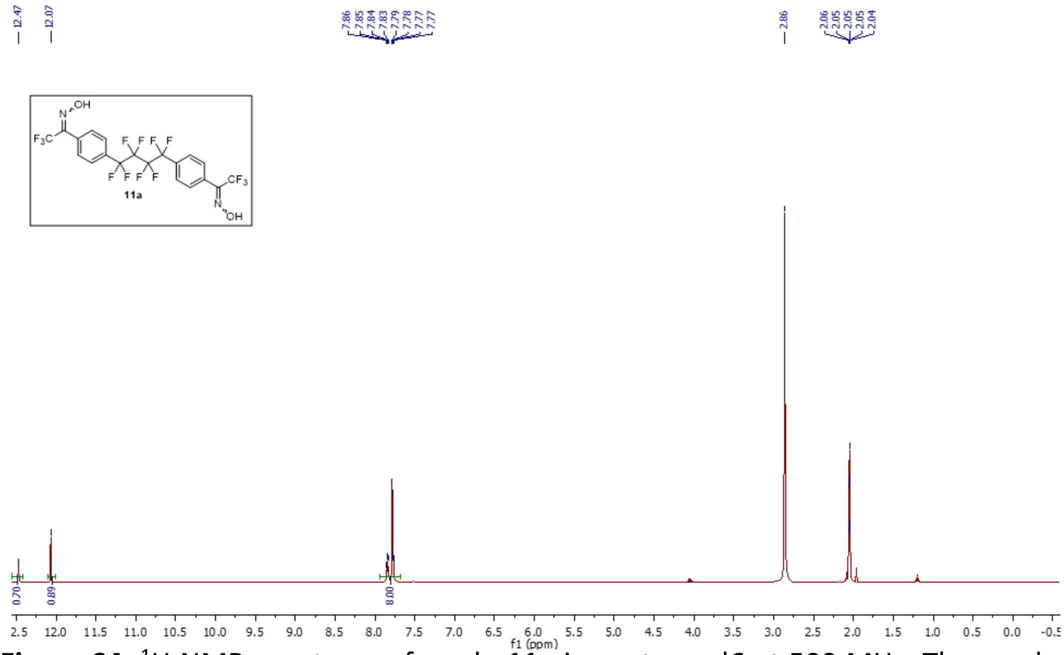

Figure S4. ^{1}H NMR spectrum of crude **11a** in acetone-d6 at 500 MHz. The product is a mixture of *E*,*E*, *E*,*Z*, and *Z*,*Z* isomers.

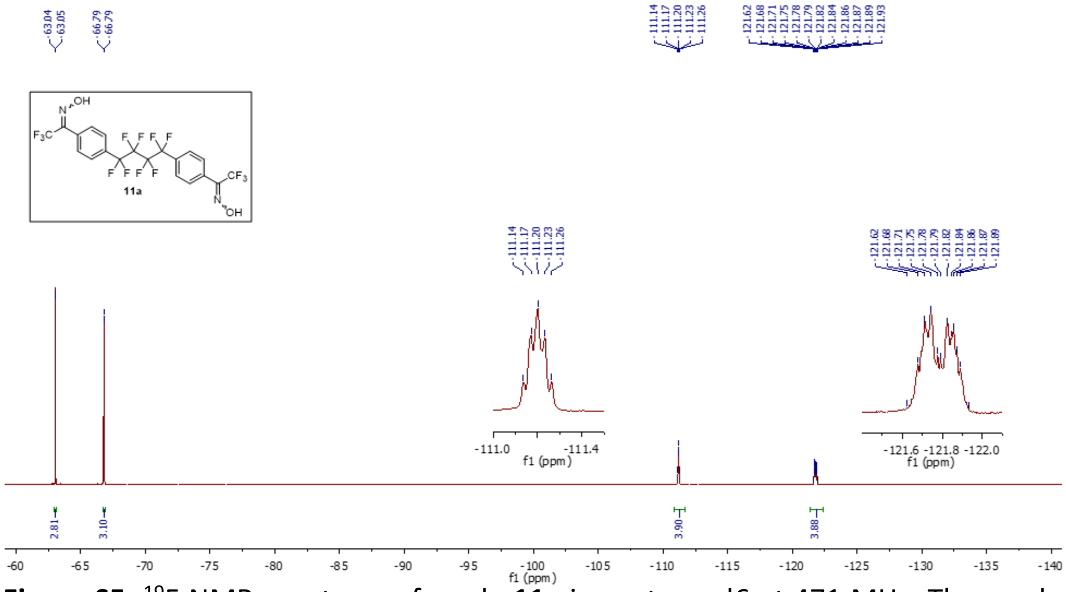

Figure S5. ^{19}F NMR spectrum of crude **11a** in acetone-d6 at 471 MHz. The product is a mixture of *E*,*E*, *E*,*Z*, and *Z*,*Z* isomers.

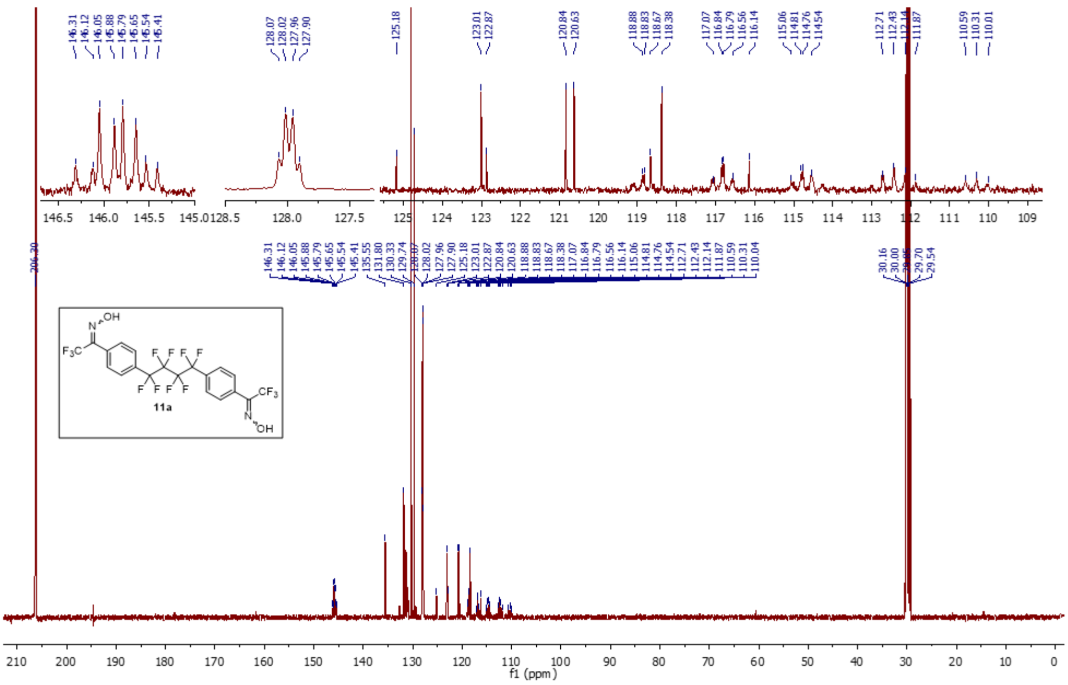

Figure S6. ^{13}C NMR spectrum of crude **11a** in acetone-d6 at 125 MHz. The product is a mixture of *E*,*E*, *E*,*Z*, and *Z*,*Z* isomers.

*2.3 Synthesis of bis-tosyl oxime **12a***

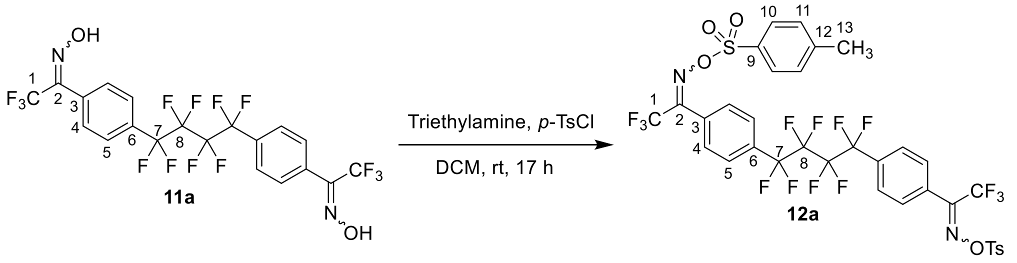

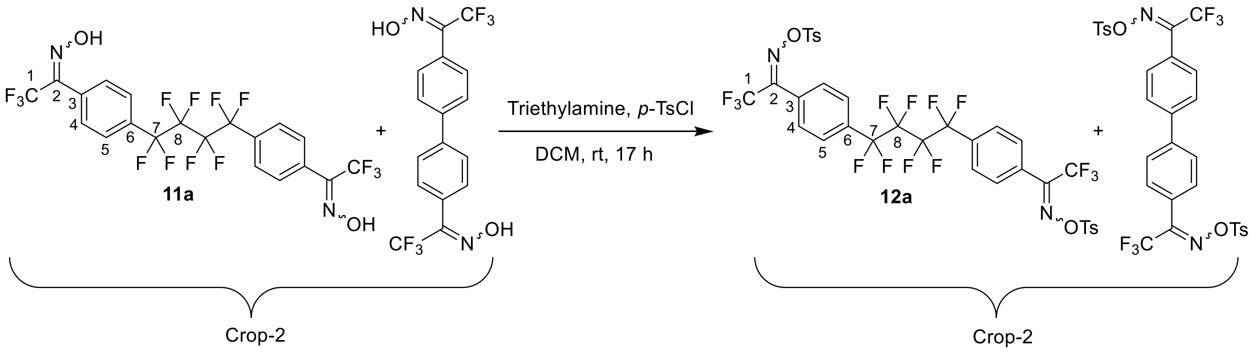

With crop-1: A suspension of crude bis-oxime **11a** (3.1 g, 5.38 mmol, 1 equiv.) in dry DCM (70 mL) was stirred at 0 °C under argon for 15 minutes. To this suspension, triethylamine (4.50 mL, 32.28 mmol, 6 equiv.) was added dropwise over 10 minutes followed by *p*-toluenesulfonyl chloride (2.15 g, 11.30 mmol, 2.1 equiv.) and DMAP (13 mg, 0.11 mmol, 0.02 equiv.) at the same temperature. The reaction mixture was then allowed to warm to room temperature and stirring was continued for 17 hours. The mixture was then diluted with DCM (100 mL) and transferred into a separatory funnel. The organic layer was then washed subsequently with water (40 mL), saturated NH_4Cl solution (2 × 30 mL), water (2 × 30 mL), and brine (1 × 30 mL), before being dried over Na_2SO_4, and concentrated to afford the desired *O*-tosyl oxime product as a white solid (5.05 g, 106% crude). Although **12a** can in principle exist as a mixture of *E*,*E*, *E*,*Z*, and *Z*,*Z* isomers, in practice one major species was observed by NMR. ^1H NMR (500 MHz, chloroform-d) δ 7.89 (d, *J* = 8.4 Hz, 4H, H-10), 7.71 (d, *J* = 8.5 Hz, 4H, H-5), 7.52 (d, *J* = 8.3 Hz,

4H, H-4), 7.40 (d, J = 8.1 Hz, 4H, H-11), 2.49 (s, 6H, H-13).^{19}F NMR (471 MHz, chloroform-d) δ –66.76 (6F), –111.17 (t, J = 13.3 Hz, 4F), –120.98 (t, J = 14.2 Hz, 4F). ^{13}C NMR (125 MHz, chloroform-d) δ 152.92 (q, $^2J_{C-F}$ = 34.1 Hz, C-2), 146.61 (C-9), 132.86 (t, $^2J_{C-F}$ = 24.9 Hz, C-6), 131.04 (C-12), 130.13 (C-11), 129.45 (C-10), 128.83 (C-4), 128.13 (C-3), 127.68 (t, $^3J_{C-F}$ = 6.0 Hz, C-5), 119.51 (q, $^1J_{C-F}$ = 277.1 Hz, C-1), 118.05–108.64 (m, C-7 and C-8), 21.91 (C-13). IR (ATR) υ (cm^{-1}): 1596, 1392, 1285, 1195, 1178, 1143,1125, 902, 818. HRMS (ESI+) m/z [M+Na$^+$] calculated for: $C_{34}H_{22}F_{14}N_2NaO_6S_2^+$: 907.0588, found: 907.0609.

With crop-2: Reaction and work up were carried out similarly to the protocol described above using 5.71 g of the crude oxime mixture, together with 100 mL of DCM, 8.3 mL of triethylamine, 3.97 g of p-TsCl and 24 mg of DMAP. After workup, 5.5 g crude product was obtained as a mixture of *O*-tosyl oximes, and was carried forward to the next step without any purification.

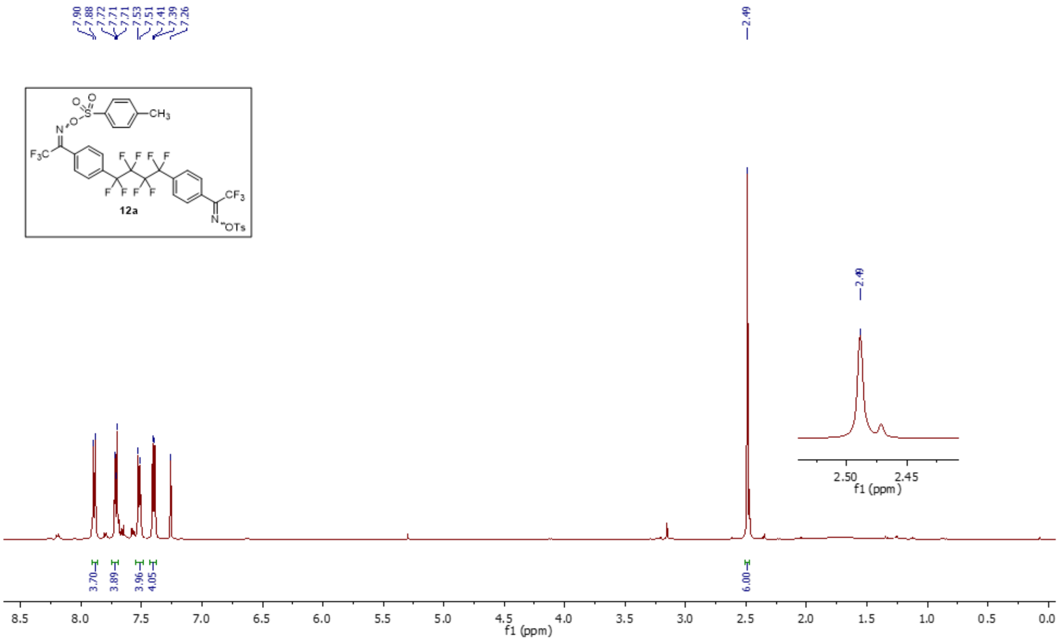

Figure S7. ^{1}H NMR spectrum of crude **12a** in chloroform-d at 500 MHz. One major geometric isomer was observed by NMR.

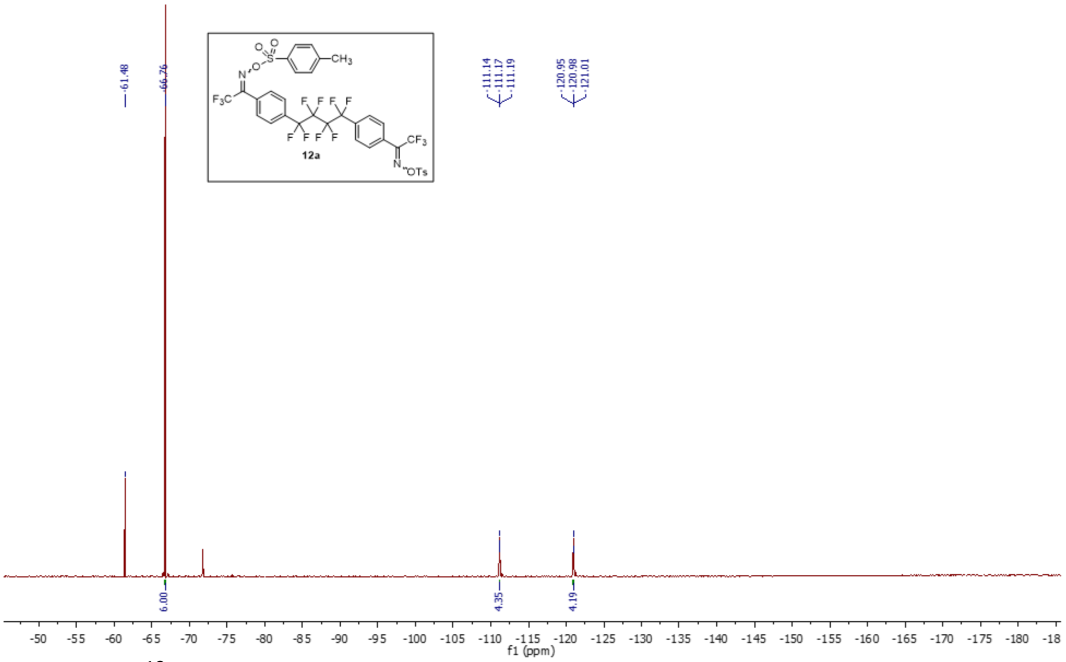

Figure S8. ^{19}F NMR spectrum of crude **12a** in chloroform-d at 471 MHz. One major geometric isomer was observed by NMR.

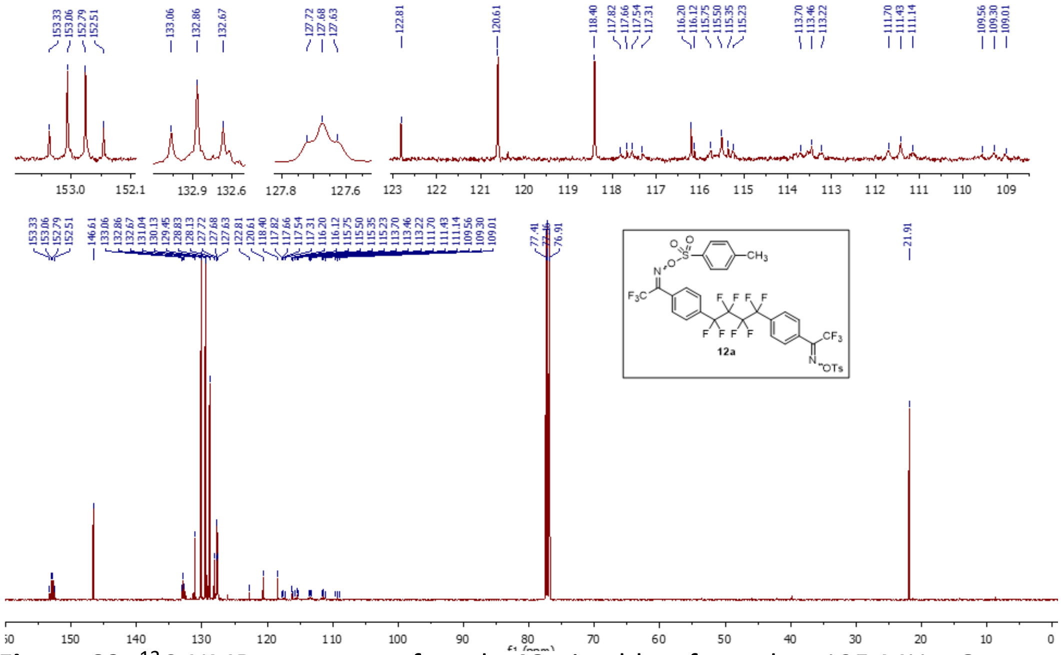

Figure S9. ^{13}C NMR spectrum of crude **12a** in chloroform-d at 125 MHz. One major geometric isomer was observed by NMR.

*2.4 Synthesis of bis-diaziridine **8a***

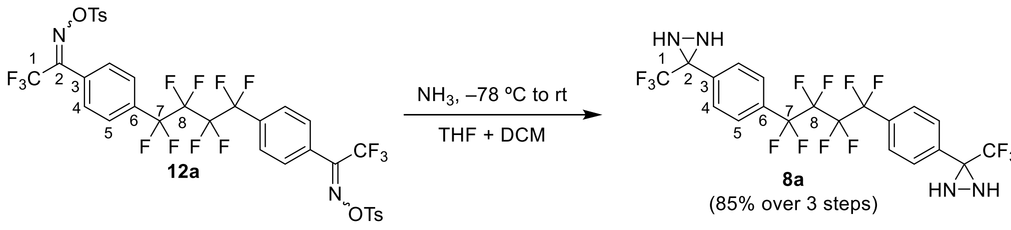

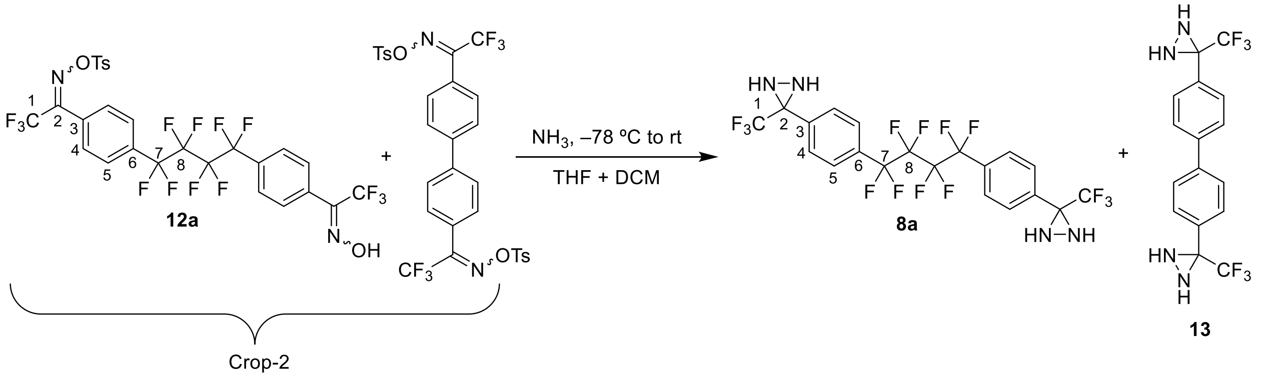

With crop-1: Ammonia gas was condensed into a oven dried three-neck 250 mL round-bottom flask that was equipped with a dewar containing dry ice and acetone on one neck, and septa on other two necks. After condensing *ca.* 100 mL of ammonia, the flask was cooled to –78 °C. A suspension of crude **12a** (4.9 g, 5.54 mmol) in a mixture of DCM and anhydrous THF (30 mL and 10 mL, respectively) was added dropwise via cannula over 10 minutes. After stirring for 2.5 hours at –78 °C, the reaction was allowed to gradually warm up to room temperature with continued stirring. After 14 h, the septa and dewar were removed from the reaction setup and stirring was continued to evaporate any remaining ammonia. The resulting white suspension was partitioned between EtOAc (200 mL) and water (30 mL). The layers were separated and the aqueous layer was extracted with EtOAc (1 × 60 mL). The combined organic layers were washed subsequently with water (1 × 40 mL) and brine (1 × 80 mL), dried over Na_2SO_4, and concentrated in vacuo. The residue was purified by silica gel flash-column chromatography, using a gradient of 15% to 25% EtOAc in hexanes to afford diaziridine **8a** (2.71 g, 85% yield over 3 steps from **6a**) as a white solid. ^{1}H NMR (500 MHz, chloroform-d) δ 7.76 (d, *J* = 8.2 Hz, 4H, H-4), 7.65 (d, *J* = 8.3 Hz, 4H, H-5), 2.87 (d, *J* = 8.9 Hz, 2H, diaziridine-H), 2.26 (d, *J* = 8.8 Hz, 2H, diaziridine-H). ^{19}F NMR (471 MHz, chloroform-d) δ –75.17 (s, 6F), –111.02 (t, *J* = 14.3 Hz, 4F), –121.22 (t, *J* = 13.9 Hz, 4F). ^{13}C NMR (125 MHz, chloroform-d) δ 135.50 (C-3), 131.63 (t, $^2J_{C\text{-}F}$ = 24.8 Hz, C-6), 128.52 (C-4), 127.59 (t, $^3J_{C\text{-}F}$ = 6.0 Hz, C-5), 123.39 (q, $^1J_{C\text{-}F}$ = 278.3 Hz, C-1), 115.69 (tt, $^1J_{C\text{-}F}$ = 257.9 Hz, $^2J_{C\text{-}F}$ = 31.0 Hz, C-7), 113.64 (tt, $^1J_{C\text{-}F}$ = 269 Hz, $^2J_{C\text{-}F}$ = 35.3 Hz, C-8), 57.80 (q, $^2J_{C\text{-}F}$ = 36.4 Hz, C-2). IR (ATR) υ (cm^{-1}): 3292, 3235, 1400, 1280, 1169, 1147, 1121, 1099, 845. HRMS (ESI+) m/z [M+H] calculated for $C_{20}H_{13}F_{14}N_4^+$: 575.0911, found: 575.0922.

With Crop-2: Reaction and work up were carried out similarly to the protocol described above using 5.5 g of crude tosyl oxime mixture, together with 40 mL of DCM, 8 mL of THF, and *ca.* 100 mL of condensed ammonia. After workup, the crude product was purified by silica gel flash-column chromatography to afford an additional 0.71 g of desired product **8a** as a white solid, and 0.4 g the homocoupled bis-diaziridine **13** as a light-yellow solid. Spectral data for **13**: ^{1}H NMR (300 MHz, chloroform-d) δ 7.71 (d, *J* = 8.2 Hz, 4H, H-4), 7.63 (dt, *J* = 8.2 Hz, 2.0 Hz 4H, H-5), 2.84 (d, *J* = 8.8 Hz, 2H, diaziridine-H), 2.26 (d, *J* = 8.8 Hz, 2H, diaziridine-H). ^{19}F NMR (283 MHz, chloroform-d) δ –75.40 (s, 6F). ^{13}C NMR (125 MHz, chloroform-d + acetone-d6) δ 142.05 (C-6), 131.57–131.04 (m,C-3), 128.84 (C-4), 127.65 (C-5), 123.64 (q, $^1J_{C\text{-}F}$ = 278.3 Hz, C-1), 57.83 (q, $^2J_{C\text{-}F}$ = 36.8 Hz, C2 from isomer 1) and 57.71 (q, $^2J_{C\text{-}F}$ = 36.1, C-2 from isomer 2).

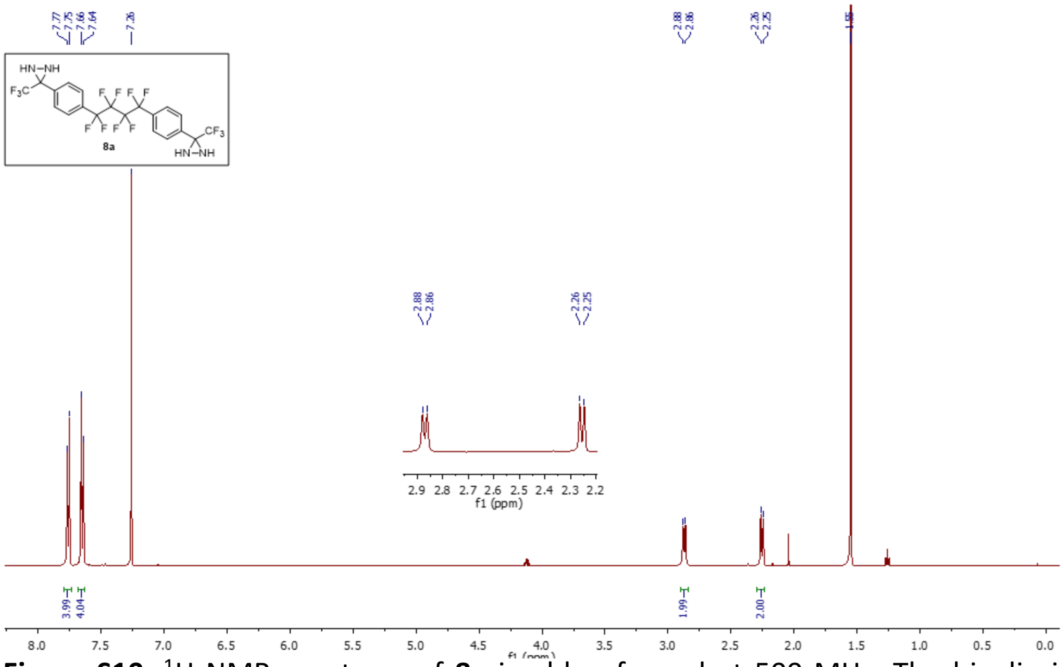

Figure S10. ^{1}H NMR spectrum of **8a** in chloroform-d at 500 MHz. The bis-diaziridine is a mixture of *meso*- and *rac*-diastereomers.

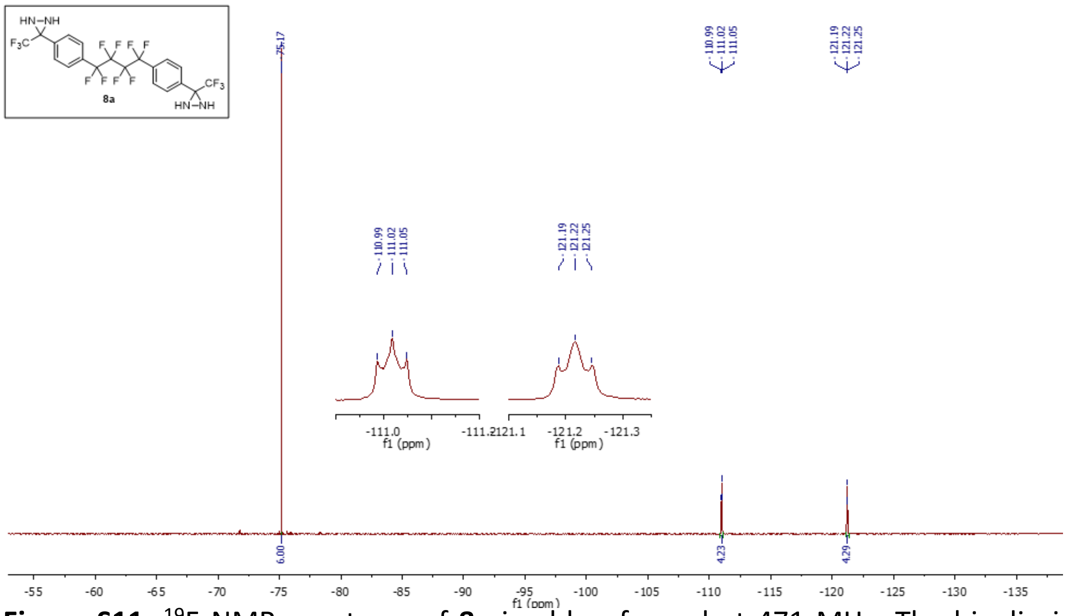

Figure S11. ^{19}F NMR spectrum of **8a** in chloroform-d at 471 MHz. The bis-diaziridine is a mixture of *meso*- and *rac*-diastereomers.

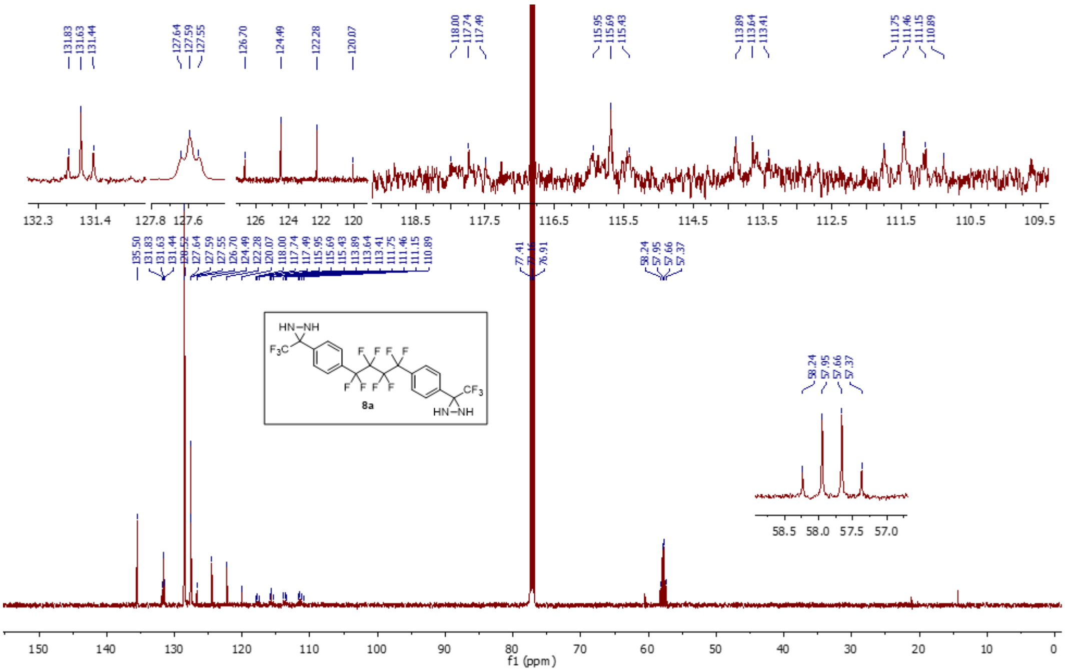

Figure S12. ^{13}C NMR spectrum of **8a** in chloroform-d at 125 MHz. The bis-diaziridine is a mixture of *meso*- and *rac*-diastereomers.

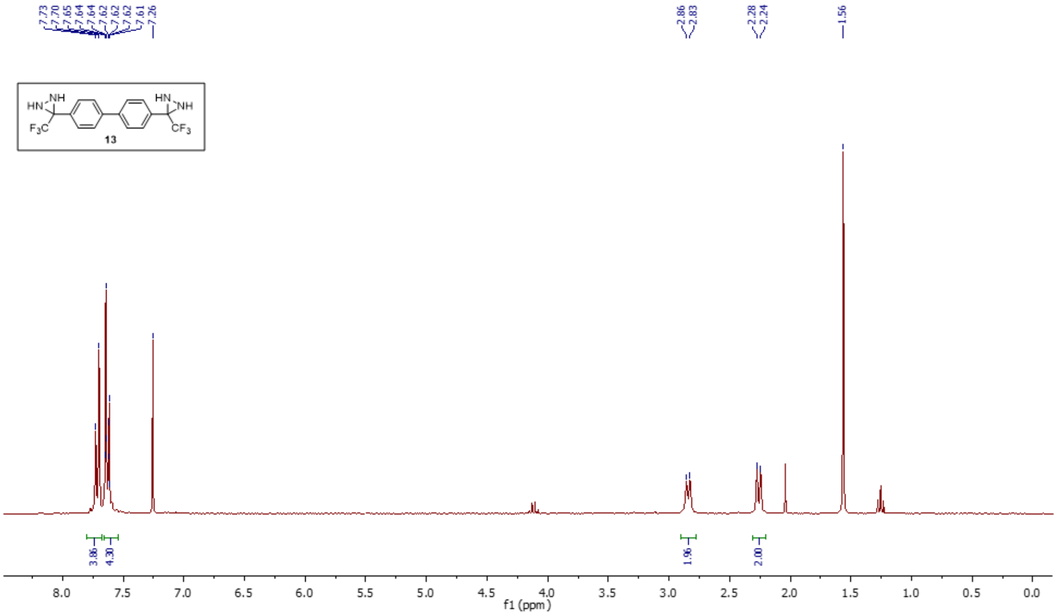

Figure S13. ^{1}H NMR spectrum of **13** in chloroform-d at 300 MHz. The bis-diaziridine is a mixture of *meso*- and *rac*-diastereomers.

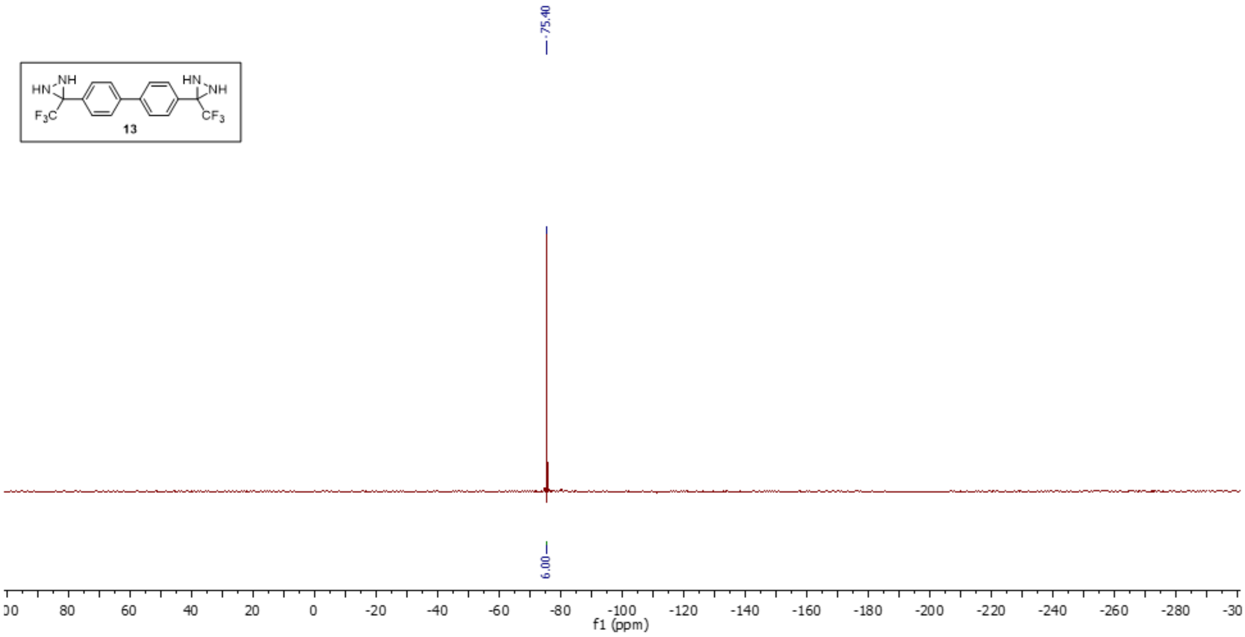

Figure S14. ^{19}F NMR spectrum of **13** in chloroform-d at 283 MHz. The bis-diaziridine is a mixture of *meso*- and *rac*-diastereomers.

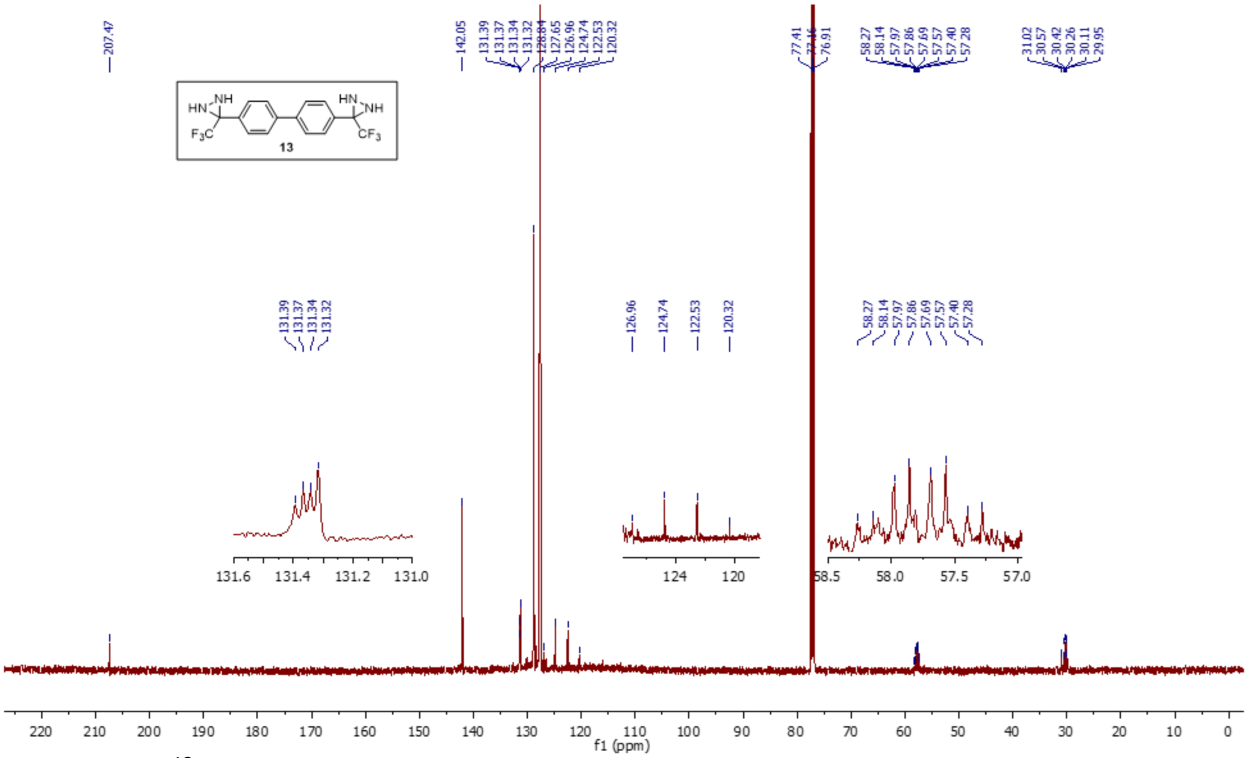

Figure S15a. ^{13}C NMR spectrum of **13** in chloroform-d + acetone-d6 at 125 MHz. The bis-diaziridine is a mixture of *meso*- and *rac*-diastereomers.

*2.5 Synthesis of bis-diazirine **2a***

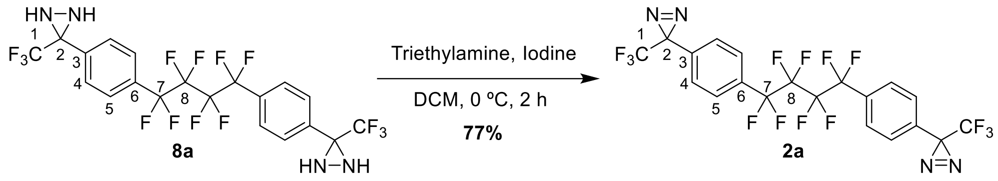

To a stirring solution of **8a** (2.1 g, 3.66 mmol) in DCM (21 mL) at 0 °C under argon, triethylamine (3.06 mL, 21.94 mmol, 6 equiv.) was added dropwise over 10 minutes. To the resulting mixture, iodine (2.04 g, 8.04, 2.2 equiv.) was added in three portions, and stirring was continued at same temperature. After 2 hours of stirring, the reaction mixture was diluted with DCM (60 mL) and transferred to a separatory funnel. The organic layer was washed subsequently with water (1× 20 mL), saturated $Na_2S_2O_3$ solution (2 × 20 mL), water (2× 30 mL), and brine (1 × 30 mL), and then dried over Na_2SO_4. To this organic layer, silica gel was added, and the mixture was concentrated in vacuo to adsorb the crude product onto the silica. This mixture was then loaded onto a silica gel column, and the desired product (1.604 g, 77%) was eluted with pentane as a shiny white crystals. ^{1}H NMR (500 MHz, chloroform-d) δ 7.61 (d, J = 8.5 Hz, 2H, H-5), 7.30 (d, J = 8.3 Hz, 2H, H-4). ^{19}F NMR (471 MHz, chloroform-d) δ –64.99 (s, 6F), –111.15 (t, J = 14.3 Hz, 4F), –121.31 (t, J = 14.3 Hz, 4F). ^{13}C NMR (125 MHz, chloroform-d) δ 133.11 (C-3), 131.03 (t, $^2J_{C\text{-}F}$ = 24.9 Hz, C-6), 127.57 (t, $^3J_{C\text{-}F}$ = 6.2 Hz, C-5), 126.71 (C-4), 121.97 (q, $^1J_{C\text{-}F}$ = 274.7 Hz, C-1), 115.59 (tt, $^1J_{C\text{-}F}$ = 256 Hz, $^2J_{C\text{-}F}$ = 32 Hz, C-7), 113.54 (tt, $^1J_{C\text{-}F}$ = 266 Hz, $^2J_{C\text{-}F}$ = 32 Hz, C-8) 28.41 (q, $^2J_{C\text{-}F}$ = 40.8 Hz, C-2). IR (ATR) υ (cm^{-1}): 1615, 1349, 1286, 1176, 1145, 1111, 940, 835. HRMS (FD+) m/z [M] calculated for $C_{20}H_8F_{14}N_4^+$: 570.0520, found: 570.0500.

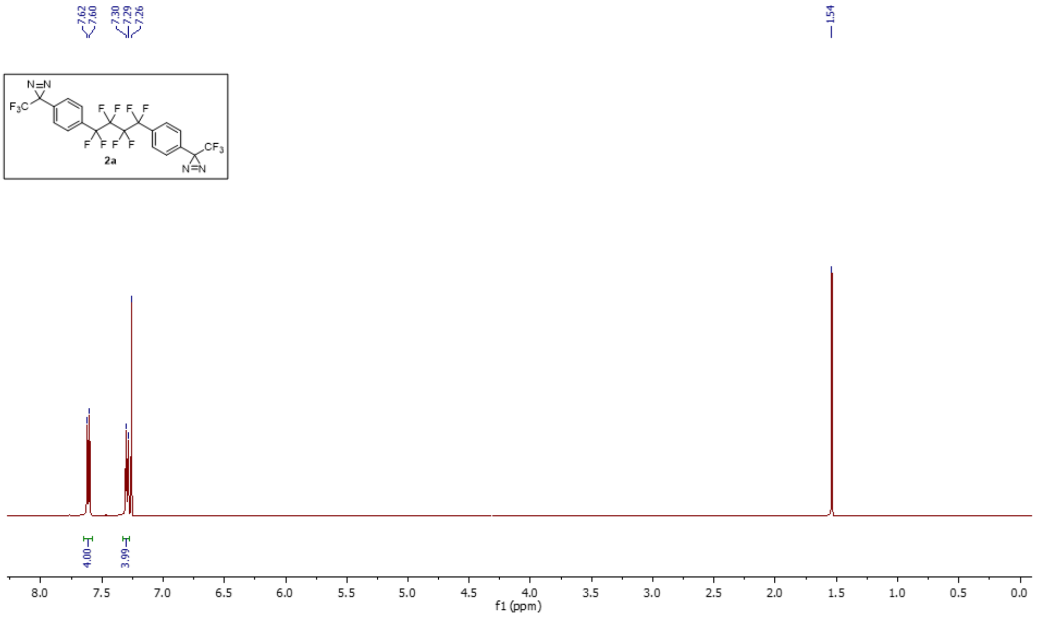

Figure S16. ^{1}H NMR spectrum of **2a** in chloroform-d at 500 MHz.

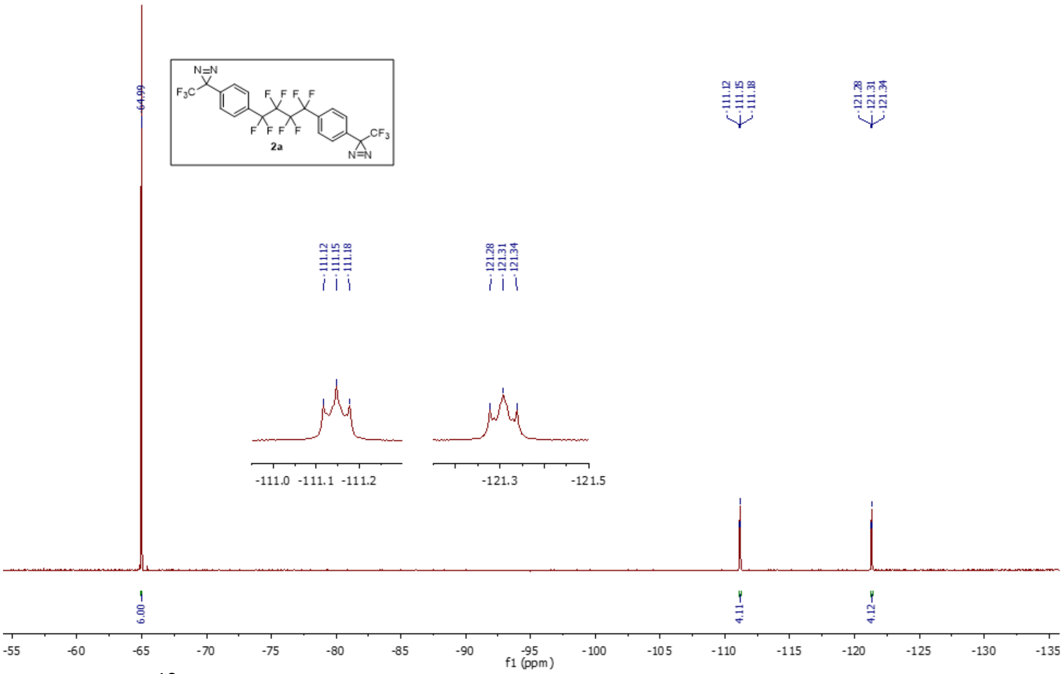

Figure S17. ^{19}F NMR spectrum of **2a** in chloroform-d at 471 MHz.

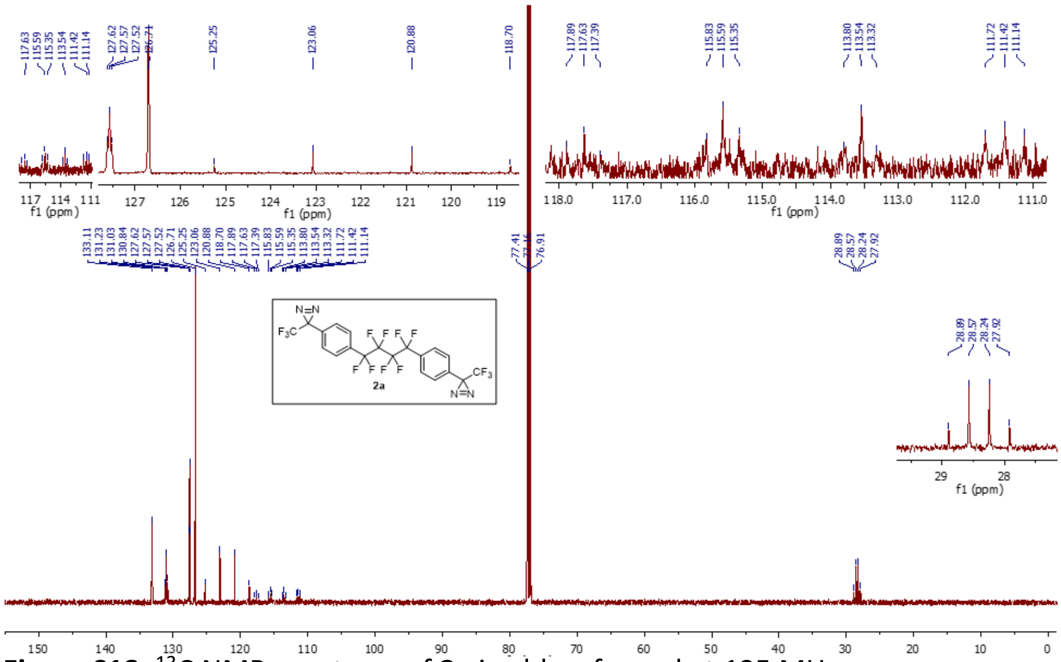

Figure S18. ^{13}C NMR spectrum of **2a** in chloroform-d at 125 MHz.

*2.6 Synthesis of bis-diazirine **14***

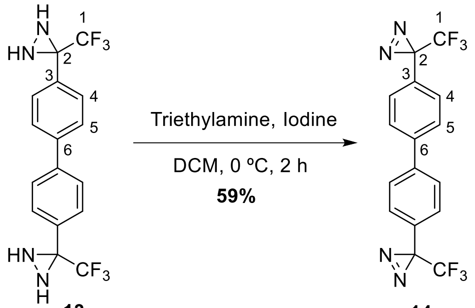

13 **14**

The reaction, work up, and purification were carried out similarly to the protocol described for the synthesis of **2a** with the following materials: **13** (0.38 g, 1.02 mmol), triethylamine (0.85 mL, 6.09 mmol), iodine (0.57 g, 2.23 mmol). Purification by flash-column chromatography afforded the desired product (220 mg, 59%) as a light-yellow solid. ^{1}H NMR (500 MHz, dichloromethane-d2) δ 7.64 (d, J = 8.5 Hz, 4H, H-5), 7.30 (d, J = 8.1 Hz, 4H, H-4). ^{19}F NMR (471 MHz, dichloromethane-d2) δ –65.62 (s, 6F). ^{13}C NMR (125 MHz, dichloromethane-d2) δ 141.48 (C-6), 129.07 (C-3), 127.92 (C-5), 127.46 (C-4), 122.61 (q, $^{1}J_{C\text{-}F}$ J = 274.5 Hz, C-1), 28.80 (q, $^{2}J_{C\text{-}F}$ = 40.5 Hz, C-2). IR (ATR) υ (cm^{-1}): 2091 (diazo isomer peak), 1606, 1606, 1501, 1345, 1236, 1188, 1140, 1120, 949, 825. HRMS (FD+) m/z [M] calculated for $C_{16}H_8F_6N_4^+$: 370.0648, found: 370.0668.

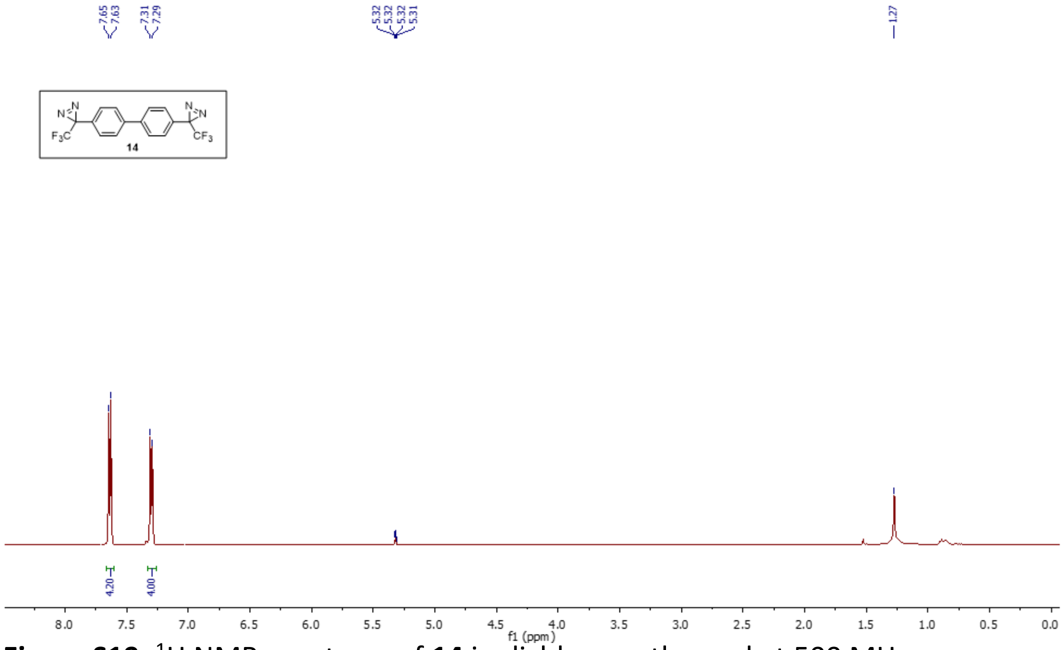

Figure S19. ^{1}H NMR spectrum of **14** in dichloromethane-d at 500 MHz.

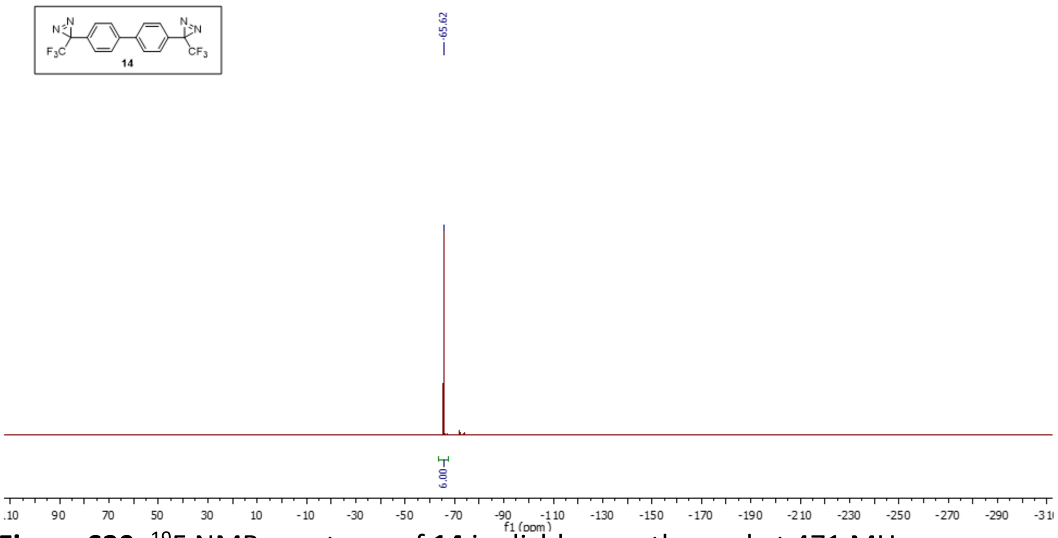

Figure S20. ^{19}F NMR spectrum of **14** in dichloromethane-d at 471 MHz.

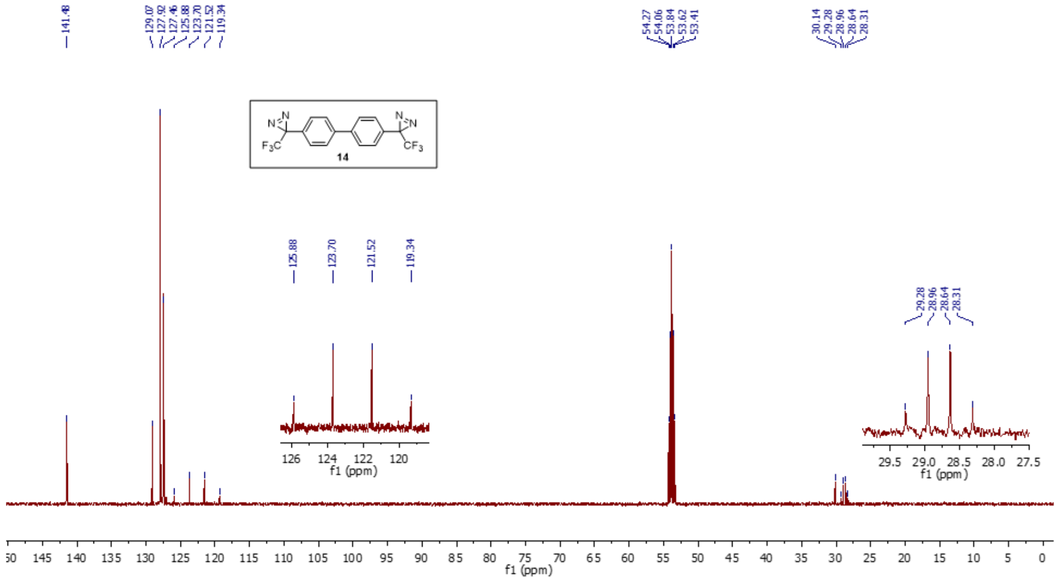

Figure S21. ^{13}C NMR spectrum of **14** in dichloromethane-d at 125 MHz.

*2.7 Synthesis of bis-ketone **6b***

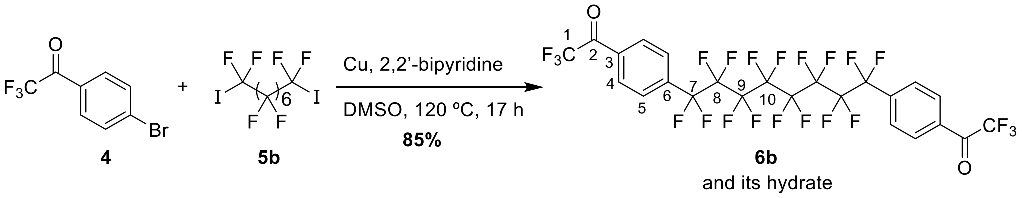

Synthesis of **6b** was performed using a modification of a literature procedure.[1] In a two-neck 250 mL round bottom flask equipped with a magnetic stir bar and a condenser, dry DMSO (37.46 mL) was added to 4'-bromo-2,2,2-trifluoroacetophenone (**4**; 11.85 g, 46.83 mmol, 1 equiv.) under argon. To this stirring solution, hexadecafluoro-1,8-diiodooctane (**5b**; 15 g, 22.95 mmol, 0.49 equiv.), copper powder (27.38 g, 430.8, 9.2 equiv.), and 2,2'-bipyridine (1.46 g, 9.37 mmol, 0.2 equiv.) were added simultaneously at room temperature. The reaction flask was evacuated and backfilled with argon and then heated to 120 °C. After 17 hours of stirring at 120 °C, the reaction mixture was cooled to room temperature, diluted with EtOAc (300 mL) followed by water (60 mL), and stirred for 10 minutes. The resultant slurry was filtered through a celite pad and the residue was washed with water followed by EtOAc. The filtrate was transferred into a separatory funnel and the layers were separated. The aqueous layer was extracted with EtOAc (3 × 70 mL). The organic layers were combined, washed subsequently with water (3 × 50 mL) and brine (1 × 50 mL), dried over Na_2SO_4, and concentrated in vacuo. The crude residue was purified by silica gel flash column chromatography (gradient of 10% to 22% EtOAc in hexanes) to afford the desired product (14.52 g, 85%) as a white solid.

Product **6b** was characterized as a mixture of the bisketone and its hydrate forms. ^{1}H NMR (500 MHz, acetone-d6) δ 8.35 (d, J = 8.2 Hz, 3.3H, H-4), 8.05 (d, J = 8.2 Hz, 3.5H, H-5), 7.99 (d, J = 8.3 Hz, 0.7H, bis-ketone hydrate H-4), 7.77 (d, J = 8.2 Hz, 0.7H, bis-ketone hydrate H-5), 6.87 (d, J = 2.5 Hz, 0.4H, bis-ketone hydrate OH). ^{19}F NMR (471 MHz, acetone-d6) δ –72.8 (s, 5.2F, CF_3), –84.7 (s, 0.8F, bis-ketone hydrate CF_3), –110.90 (t, J = 14.9 Hz, 0.5F), –111.76 (t, J = 14.4 Hz, 3.5 F), –121.66 (s, 4F), –122.2 – –122.3 (m, 8F). ^{13}C NMR (125 MHz, acetone-d6) δ 180.60 (q, $^2J_{C\text{-}F}$ = 35.6 Hz, C-2), 143.77 (bis-ketone hydrate C-3), 135.38 (td, $^2J_{C\text{-}F}$ = 24.2, $^1J_{C\text{-}F}$ = 3.1 Hz, C-6), 134.14 (C-3), 131.32 (q, $^4J_{C\text{-}F}$ = 2.2 Hz, C-4), 130.12 (t, $^2J_{C\text{-}F}$ = 23.3 Hz, bis-ketone hydrate C-6), 129.19 (bis-ketone hydrate C-4), 128.93 (t, $^3J_{C\text{-}F}$ = 6.6 Hz, C-5), 127.36 (t, $^3J_{C\text{-}F}$ = 6.5 Hz, bis-ketone hydrate C-5), 124.26 (q, $^1J_{C\text{-}F}$ = 287.6 Hz, bis-ketone hydrate C-1), 117.42 (q, $^1J_{C\text{-}F}$ = 290.5 Hz, C-1), 119.15–108.88 (m, bis-ketone core 4 × CF_2 and bis-ketone hydrate core 4 × CF_2), 93.88 (q, $^2J_{C\text{-}F}$ = 32.1 Hz) and 93.87 (q, $^2J_{C\text{-}F}$ = 32.1 Hz, bis-ketone hydrate C-2). IR (ATR) υ (cm^{-1}): 1725, 1417, 1305, 1175, 1151, 1136, 1120, 1101, 849. HRMS (ESI+) m/z [M+H] calculated $C_{24}H_9F_{22}O_2^+$: 747.0246, found: 747.0230.

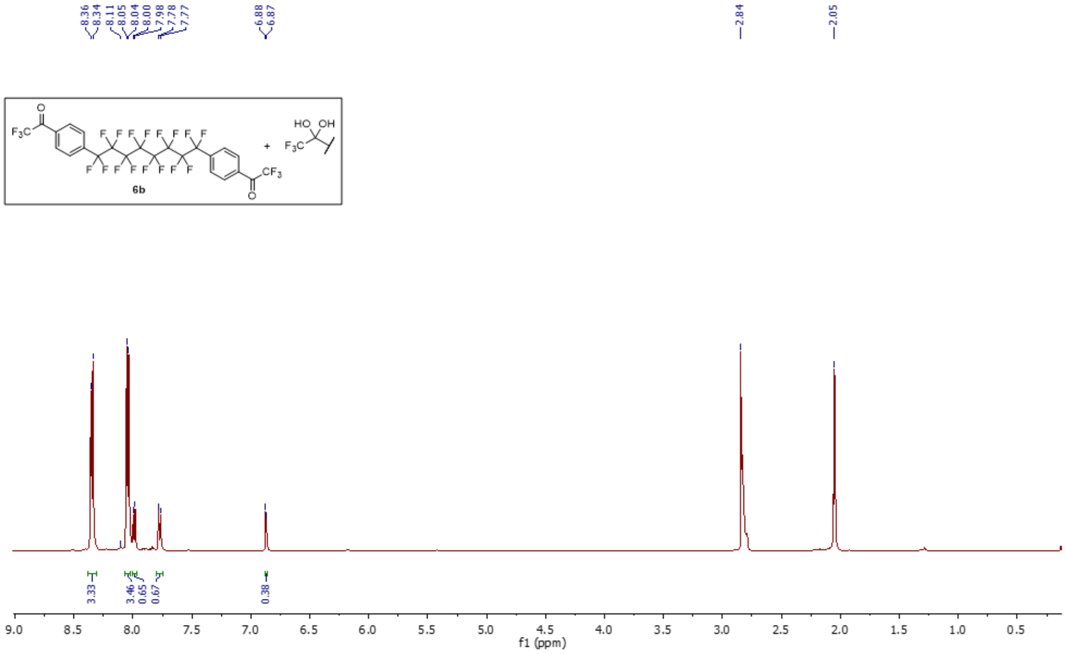

Figure S22. ^{1}H NMR spectrum of **6b** in acetone-d6 at 500 MHz. The product is a mixture of ketone and hydrate forms.

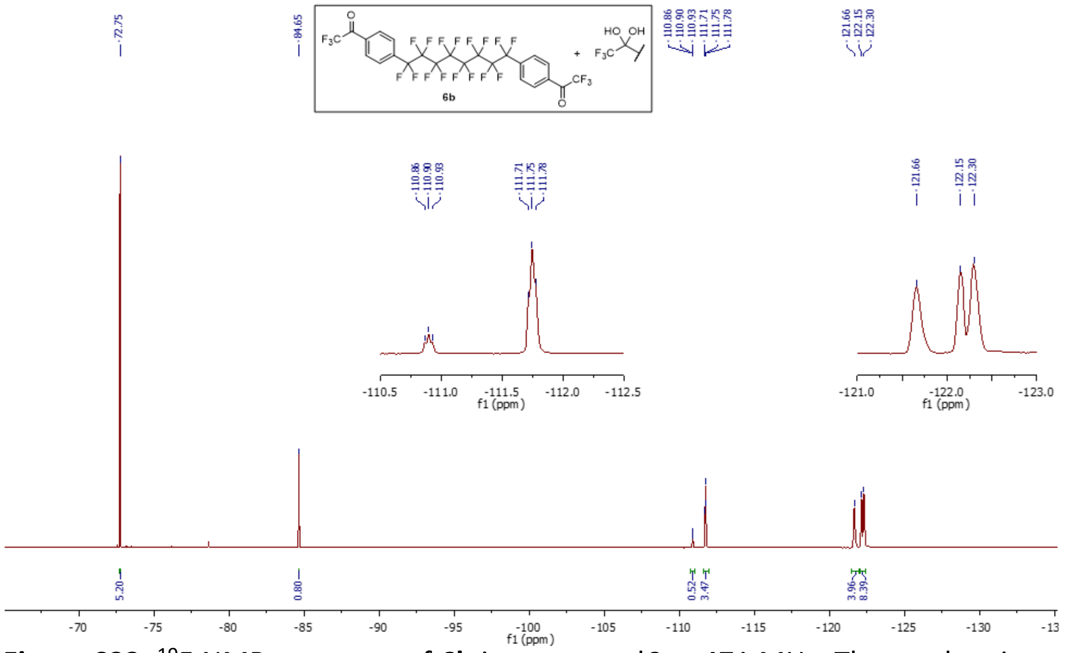

Figure S23. ^{19}F NMR spectrum of **6b** in acetone-d6 at 471 MHz. The product is a mixture of ketone and hydrate forms.

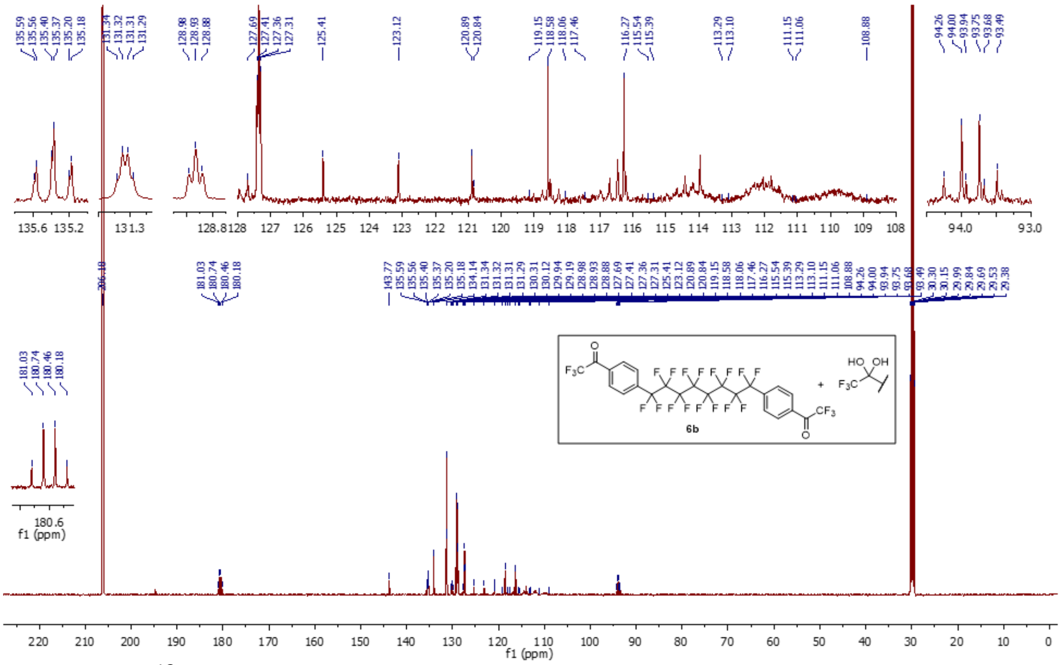

Figure S24. ^{13}C NMR spectrum of **6b** in acetone-d6 at 125 MHz. The product is a mixture of ketone and hydrate forms.

*2.8 Synthesis of bis-oxime **11b***

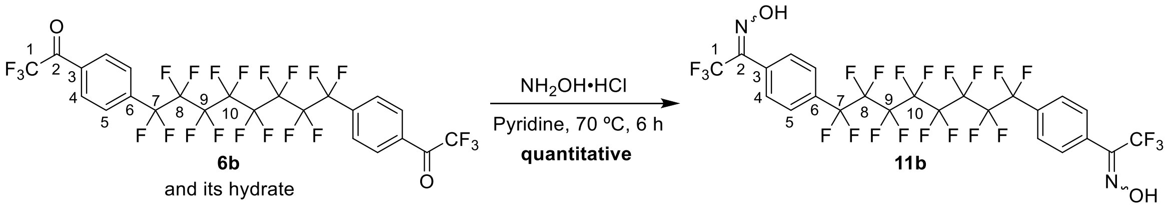

To a stirring solution of **6b** (14.44 g, 19.35 mmol, 1 equiv.) in pyridine (220 mL), hydroxylamine hydrochloride (8.07 g, 116.1 mmol, 6 equiv.) was added at room temperature under argon. The reaction mixture was heated to 70 °C. After 6 hours of stirring at 70 °C, the reaction mixture was allowed to cool to room temperature and was then concentrated to remove pyridine. The resulting residue was partitioned between EtOAc (400 mL) and water (80 mL). The aqueous layer was further extracted with EtOAc (2 × 50 mL). The organic layers were combined and subsequently washed with water (1 × 60 mL), 1N HCl (2 × 50 mL), water (2 × 60 mL), and brine (1 × 50 mL). The organic layer was dried over Na_2SO_4 and concentrated to afford the desired product (15.8 g, 105% crude) as a white solid, which was subjected tot he next step without purification. The product is a mixture of *syn*- and *anti*- isomers. ^1H NMR (500 MHz, acetone-d6) δ 12.45 (s, oxime-OH), 12.04 (s, oxime-OH), 7.92–7.79 (m, 8H). ^{19}F NMR (471 MHz, acetone-d6) δ –63.06 (s, 2.37F), –66.84 (s, 3.65F), –111.19 (dt, *J* = 29.3, 14.3 Hz, 3.22F), –121.71 (s, 4.17F), –122.12 – –122.41 (m, 8.61F). ^{13}C NMR (125 MHz, acetone-d6) δ 145.85 (q, $^2J_{C\text{-}F}$ = 32.5 Hz) and 145.82 (q, $^2J_{C\text{-}F}$ = 30.4 Hz), 135.90, 132.15, 130.49, 129.87, 128.02 (q, *J* = 7.0 Hz), 121.93 (q, $^1J_{C\text{-}F}$ = 273.1 Hz) and 119.52 (q, $^2J_{C\text{-}F}$ = 282.1 Hz), 116.78 (tt, *J* = 257,8, *J* = 30.8 Hz), 114.8–109.18 (m). IR (ATR) υ (cm^{-1}): 3300, 1410, 1304, 1172, 1136, 1102, 959, 832. HRMS (ESI–) m/z [M–H] calculated for $C_{24}H_9F_{22}N_2O_2^-$: 775.0318, found: 775.0295.

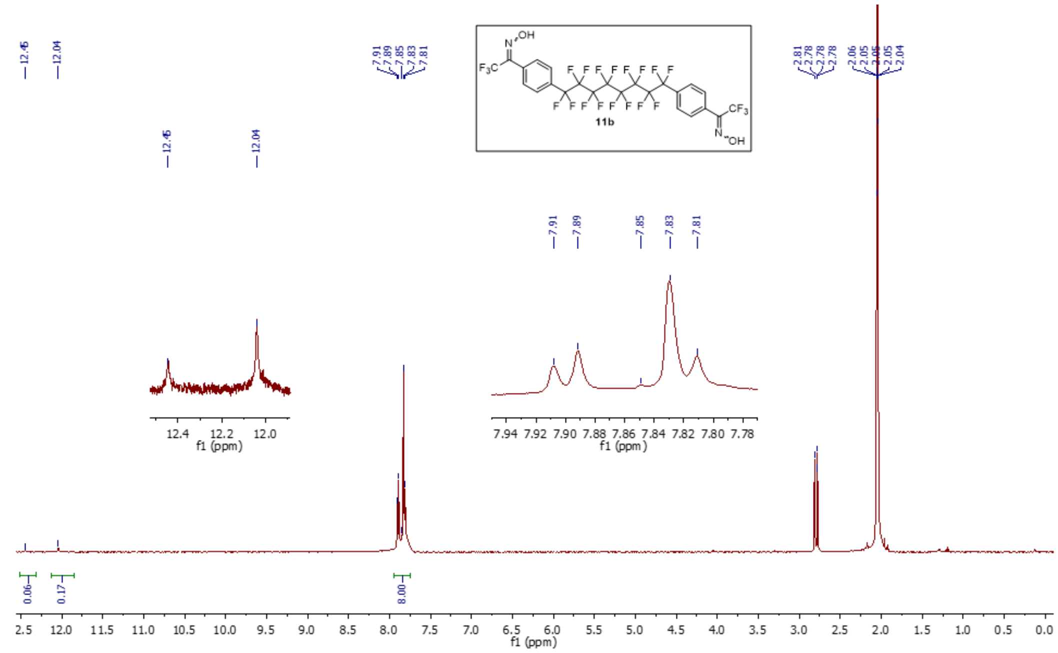

Figure S25. ^{1}H NMR spectrum of **11b** in acetone-d6 at 500 MHz. The product is a mixture of *E*,*E* *E*,*Z*, and *Z*,*Z* isomers.

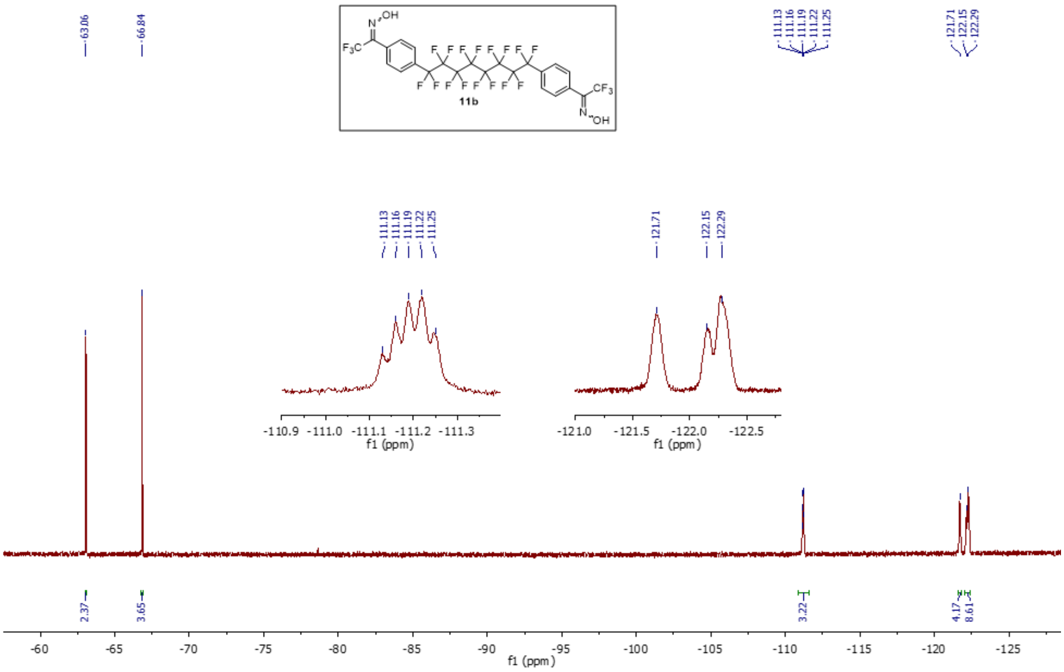

Figure S26. ^{19}F NMR spectrum of **11b** in acetone-d6 at 471 MHz. The product is a mixture of *E*,*E* *E*,*Z*, and *Z*,*Z* isomers.

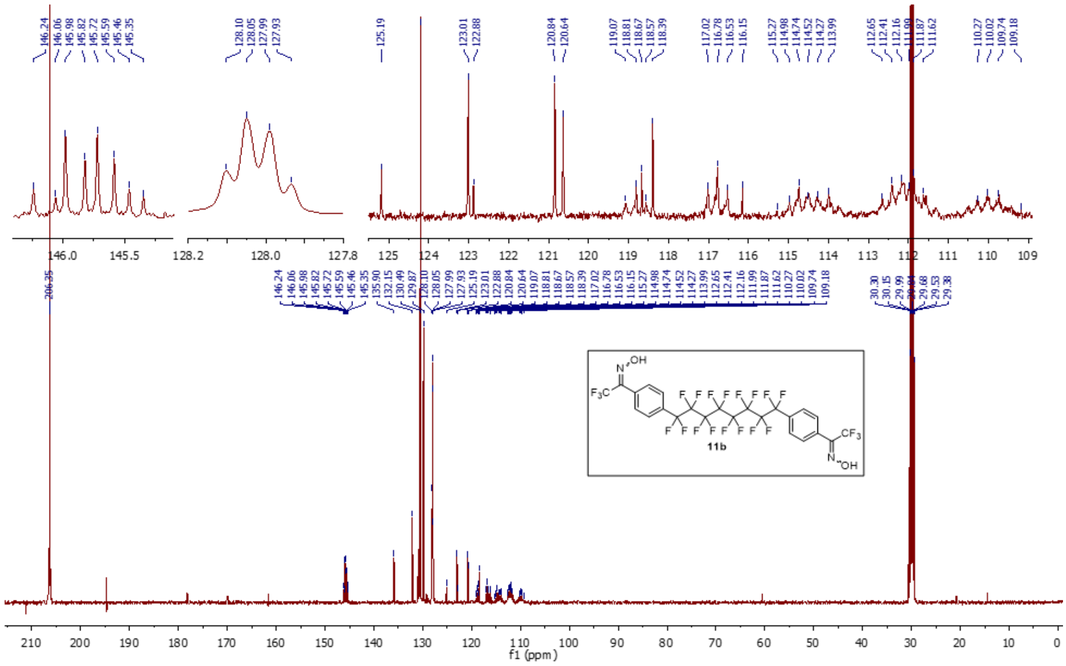

Figure S27. ^{13}C NMR spectrum of **11b** in acetone-d6 at 125 MHz. The product is a mixture of *E*,*E* *E*,*Z*, and *Z*,*Z* isomers.

*2.9 Synthesis of tosyl oxime **12b***

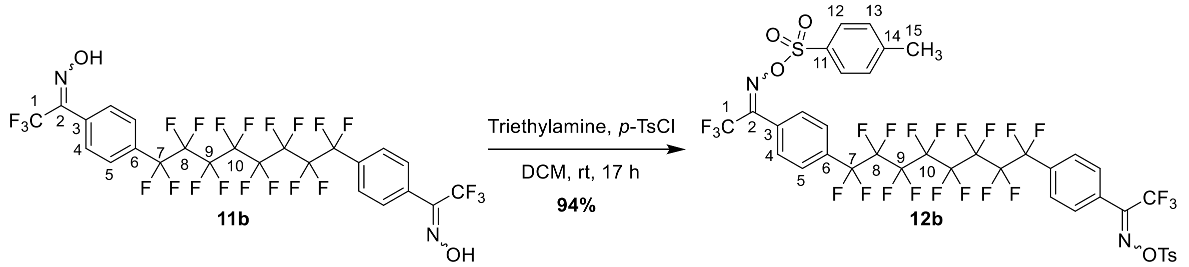

A suspension of crude bis-oxime **11b** (15.0 g crude, 19.32 mmol) in dry DCM (150 mL) was stirred at 0 °C under argon for 15 minutes. To this suspension, triethylamine (16.16 mL, 115.9 mmol, 6 equiv.) was added dropwise over 10 minutes followed by *p*-toluenesulfonyl chloride (7.74 g, 40.58 mmol, 2.1 equiv.) and DMAP (47 mg, 0.39 mmol, 0.02 equiv.) at same temperature. The reaction mixture was allowed to warm to room temperature and stirring was continued for 17 hours. The mixture was then diluted with DCM (300 mL) and transferred into a separatory funnel. The organic layer was then washed subsequently with water (1 × 50 mL), saturated NH_4Cl solution (2 × 100 mL), water (2 × 100 mL), and brine (1 × 50 mL), before being dried over Na_2SO_4, and concentrated to afford the desired *O*-tosyl oxime product (19.63 g, 94% crude) as a white solid. The obtained product is a mixture of *syn*- and *anti*- isomers. ^{1}H NMR (500 MHz, chloroform-d) δ 7.90 (dd, *J* = 8.5, 6.9 Hz, 6H), 7.72 (dd, *J* = 8.5, 1.8 Hz, 4H), 7.70–7.64 (m, 3H), 7.59 (d, *J* = 8.2 Hz, 3H), 7.54 (d, *J* = 8.2 Hz, 4H), 7.43–7.36 (m, 6H), 2.49 (s, 6H), 2.47 (s, 4H). ^{19}F NMR (283 MHz, chloroform-d) δ –61.56 (3.85F), –66.87 (6.00F), –108.28 – –115.89 (m, 5.8F), –120.75 – –122.33 (m, 17.0F). ^{13}C NMR (125 MHz, chloroform-d + 2 drops acetone-d6) δ 152.70 (q, *J* = 34.3 Hz) and 152.62 (q, *J* = 32.6 Hz), 146.54 and 146.2 (q, *J* = 1.9 Hz), 132.17 (t, *J* = 24.5 Hz) and 132.12 (t, *J* = 24.8 Hz), 131.42 and 128.27, 131.11 and 130.88, 130.02 (t, *J* = 2.2 Hz), 129.26 (d, *J* = 1.6 Hz) and 128.81, 129.23 – 129.03 (m), 127.53 (t, *J* = 6.3 Hz) and 127.37 (t, *J* = 6.7 Hz), 119.34 (q, *J* = 278.0 Hz), 115.37 (tt, *J* = 257.9 Hz, *J* = 32.8 Hz), 113.49–108.28 (m), 21.71. IR (ATR) υ (cm^{-1}): 1596, 1393, 1296, 1195, 1179, 1143, 1108, 899, 818. HRMS (ESI+) m/z [M+Na] calculated for $C_{38}H_{22}F_{22}N_2NaO_6S_2^+$: 1107.0460, found: 1107.0486.

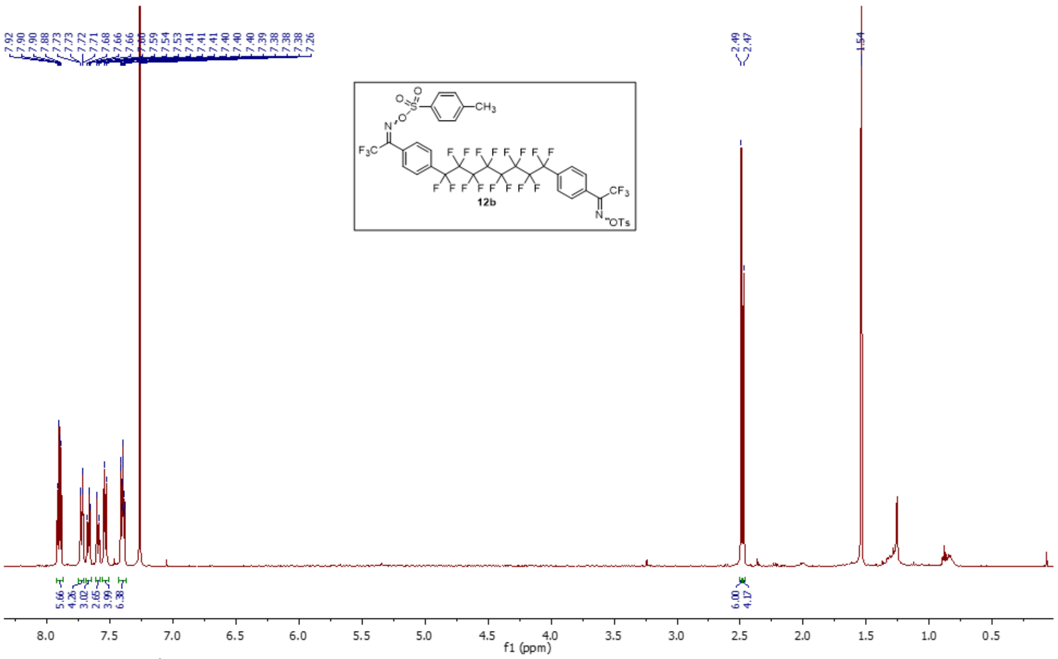

Figure S28. ^{1}H NMR spectrum of **12b** in chloroform-d at 500 MHz. The product is a mixture of *syn*- and *anti*-isomers.

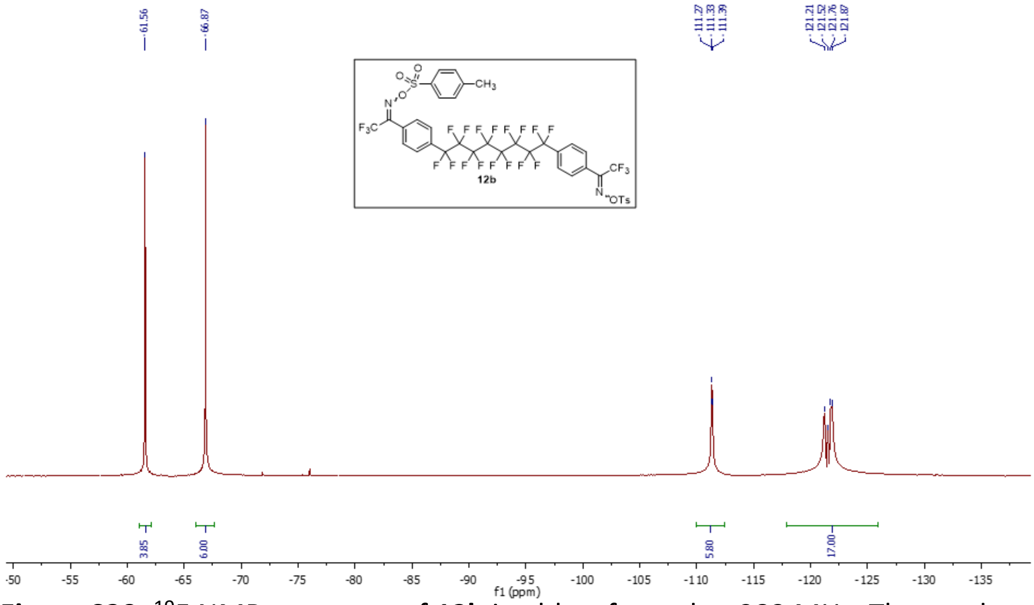

Figure S29. ^{19}F NMR spectrum of **12b** in chloroform-d at 283 MHz. The product is a mixture of *syn*- and *anti*-isomers.

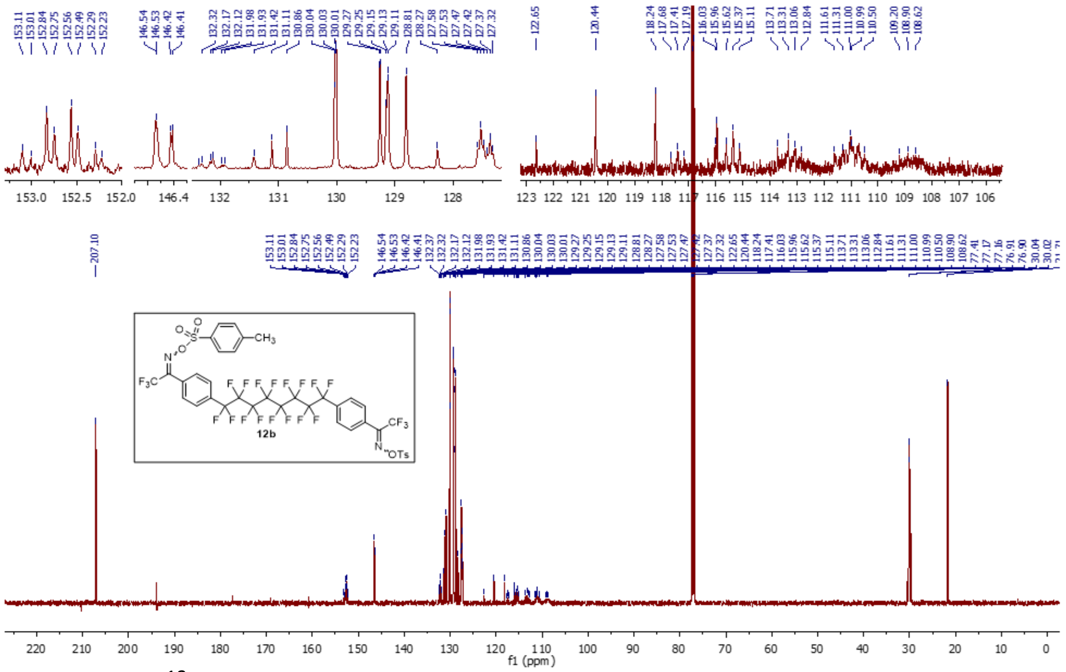

Figure S30. ^{13}C NMR spectrum of **12b** in chloroform-d + acetone-d6 at 125 MHz. The product is a mixture of *syn-* and *anti-*isomers.

2.10 Synthesis of bis-diaziridine ***8b***

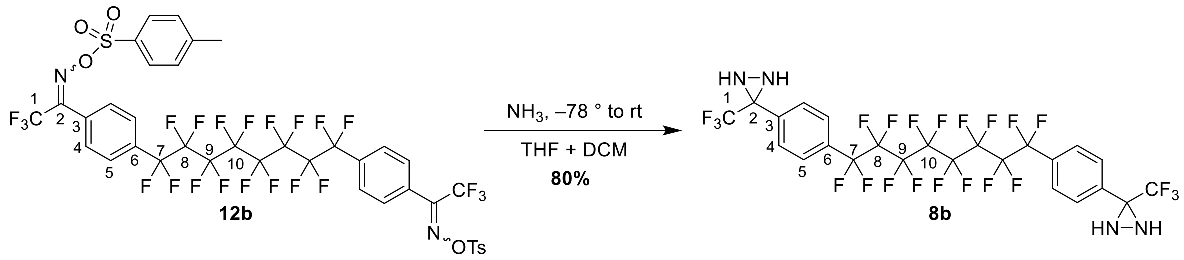

Reaction and work up were carried out similarly to the protocol described for the of synthesis **8a**, using the following materials: **12a** (19.58 g, 18.05 mmol) in a mixture of DCM (30 mL) and anhydrous THF (50 mL), and condensed ammonia (*ca.* 200 mL). The crude product was purified by silica gel flash-column chromatography, using a gradient of 15% to 40% EtOAc in hexanes to afford diaziridine **8b** (11.21 g, 80%) as white solid. ^{1}H NMR (500 MHz, chloroform-d) δ 7.79 (d, J = 8.2 Hz, 4H, H-4), 7.67 (d, J = 8.5 Hz, 4H, H-5), 2.88 (d, J = 8.8 Hz, 2H, diaziridine-H), 2.27 (d, J = 8.8 Hz, 2H, diaziridine-H). ^{19}F NMR (471 MHz, chloroform-d) δ –75.15 (s, 6F), –110.99 (t, J = 14.5 Hz, 4F), –121.17 (broad-s, 4F), –121.73 (broad-s, 8F). ^{13}C NMR (125 MHz, chloroform-d) δ 135.82 (C-3), 131.14 (t, $^{2}J_{C\text{-}F}$ = 24.5 Hz, C-6), 128.65 (C-4), 127.61 (t, $^{3}J_{C\text{-}F}$ = 6.4 Hz, C-5), 123.39 (q, $^{1}J_{C\text{-}F}$ = 278.1 Hz, C-1), 115.67 (tt, $^{1}J_{C\text{-}F}$ = 257.4 Hz, $^{2}J_{C\text{-}F}$ = 30.97 Hz, C-7), 114.00–107.94 (m, C-8, C-9, and C-10), 57.80 (q, $^{2}J_{C\text{-}F}$ = 36.4 Hz, C-2). IR (ATR) υ (cm^{-1}): 3267, 1398, 1301, 1137, 1112, 1099, 828. HRMS (ESI+) m/z [M+H] calculated for $C_{24}H_{13}F_{22}N_4^+$: 775.0783, found 775.0804.

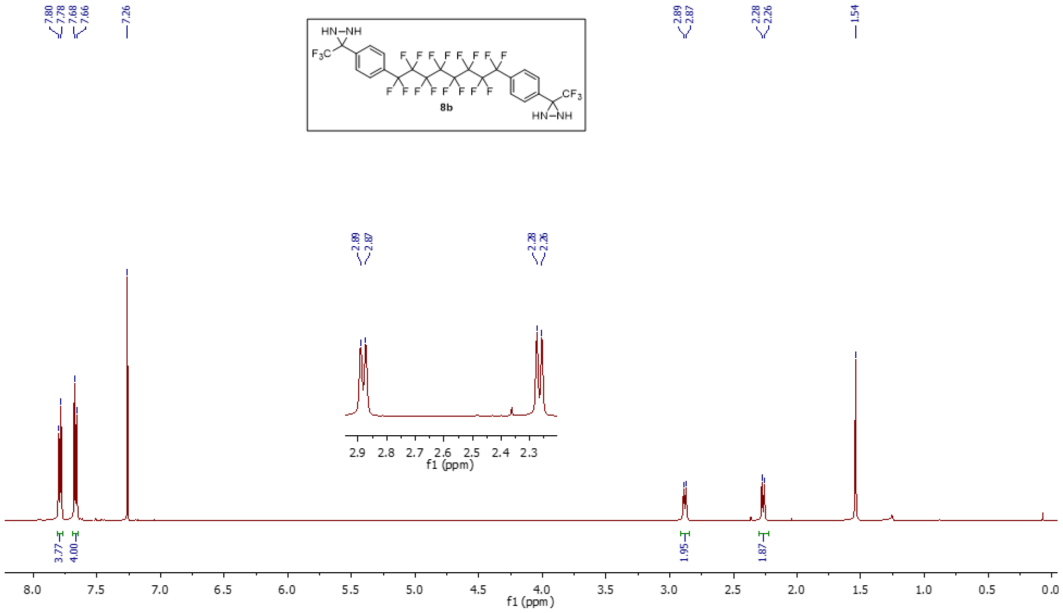

Figure S31. ^{1}H NMR spectrum of **8b** in chloroform-d at 500 MHz. The bis-diaziridine is a mixture of *meso*- and *rac*-diastereomers.

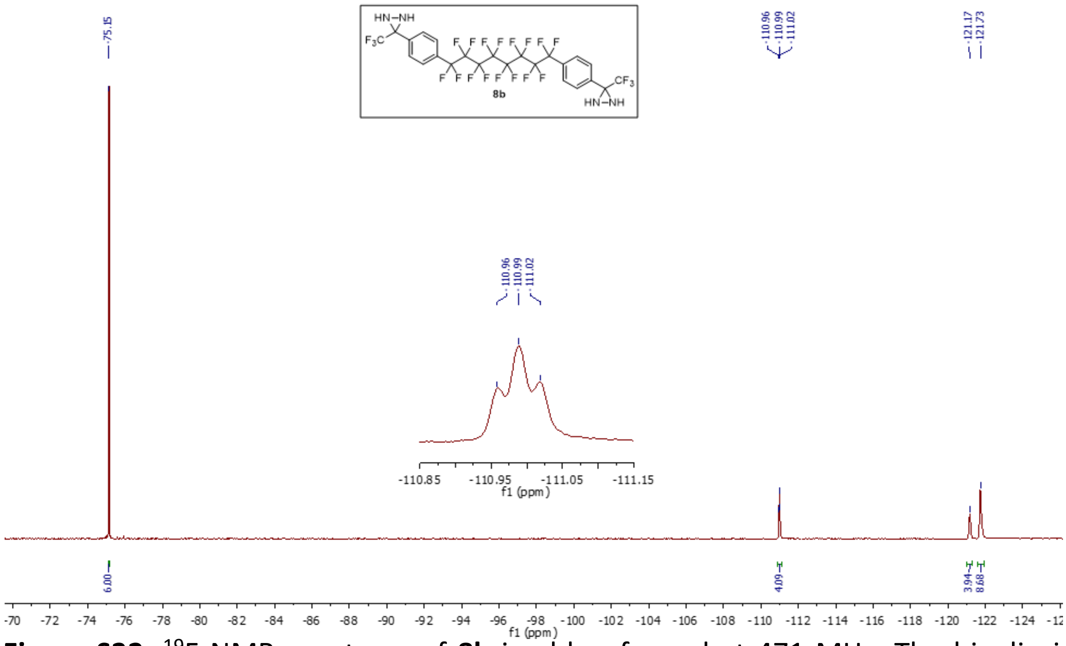

Figure S32. ^{19}F NMR spectrum of **8b** in chloroform-d at 471 MHz. The bis-diaziridine is a mixture of *meso*- and *rac*-diastereomers.

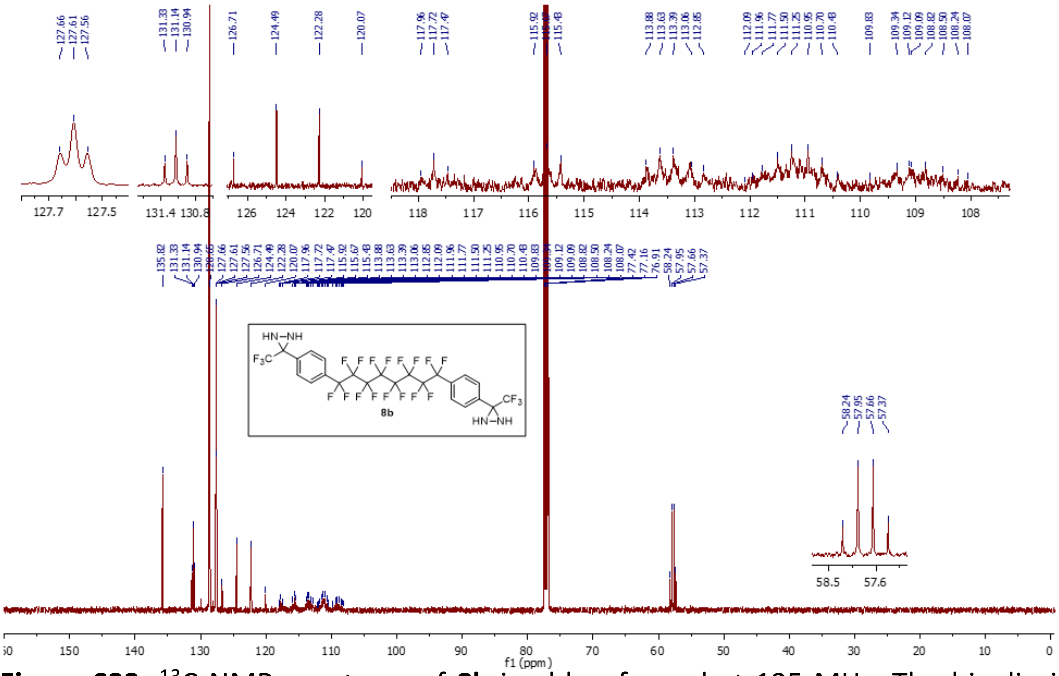

Figure S33. ^{13}C NMR spectrum of **8b** in chloroform-d at 125 MHz. The bis-diaziridine is a mixture of *meso*- and *rac*-diastereomers.

*2.11 Synthesis of bis-diazirine **2b***

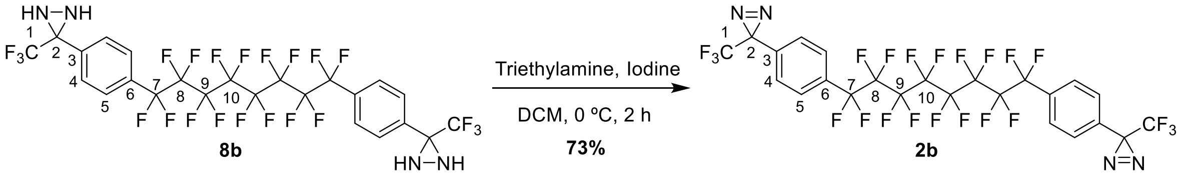

Reaction, work up, and purification were carried out similarly to the protocol described for the of synthesis **2a**, using the following materials: **8b** (11.2 g, 14.47 mmol) in DCM (110 mL), triethylamine (12.1 mL, 86.82 mmol, 6 equiv.) and iodine (8.07 g, 31.83 mmol, 2.2 equiv.). The desired product **2b** (8.12 g, 73% yield) was obtained as a shiny white crystals. ^{1}H NMR (300 MHz, chloroform-d) δ 7.63 (d, *J* = 8.5 Hz, 4H, H-5), 7.32 (d, *J* = 8.3 Hz, 4H, H-4). ^{19}F NMR (283 MHz, chloroform-d) δ –64.98 (s, 6F), –111.16 (t, *J* = 14.3 Hz, 4F), –121.23 (broad s, 8F). ^{13}C NMR (125 MHz, chloroform-d) δ 133.43 (t, $^{3}J_{C\text{-}F}$ = 1.7 Hz, C-3), 130.57 (t, $^{2}J_{C\text{-}F}$ = 24.6 Hz, C-6), 127.58 (t, $^{3}J_{C\text{-}F}$ = 6.5 Hz, C-5), 126.82 (C-4), 121.98 (q, $^{1}J_{C\text{-}F}$ = 274.7 Hz, C-1), 118.6–108.41 (m, C-7, C-8, C-9 and C-10), 28.42 (q, $^{2}J_{C\text{-}F}$ = 41.0 Hz). IR (ATR) υ (cm^{-1}): 1615, 1348, 1304, 1148, 1136, 1101, 940, 818. HRMS (FD+) m/z [M] calculated for $C_{24}H_8F_{22}N_4$+: $C_{24}H_8F_{22}N_4$: 770.0392, found: 770.0388.

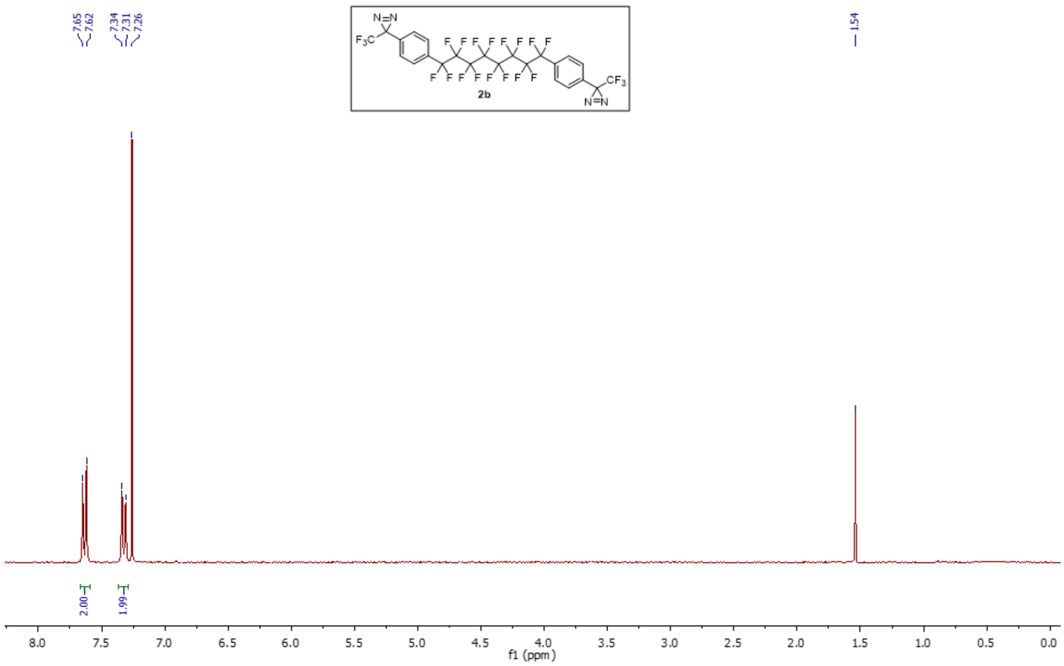

Figure S34. ^{1}H NMR spectrum of **2b** in chloroform-d at 300 MHz.

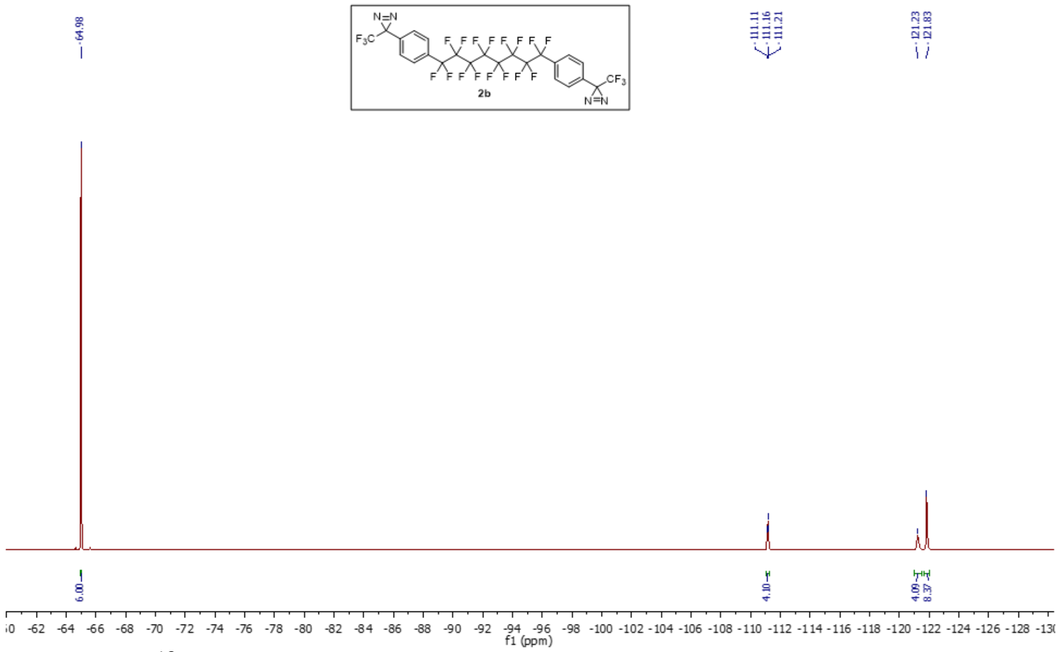

Figure S35. ^{19}F NMR spectrum of **2b** in chloroform-d at 283 MHz.

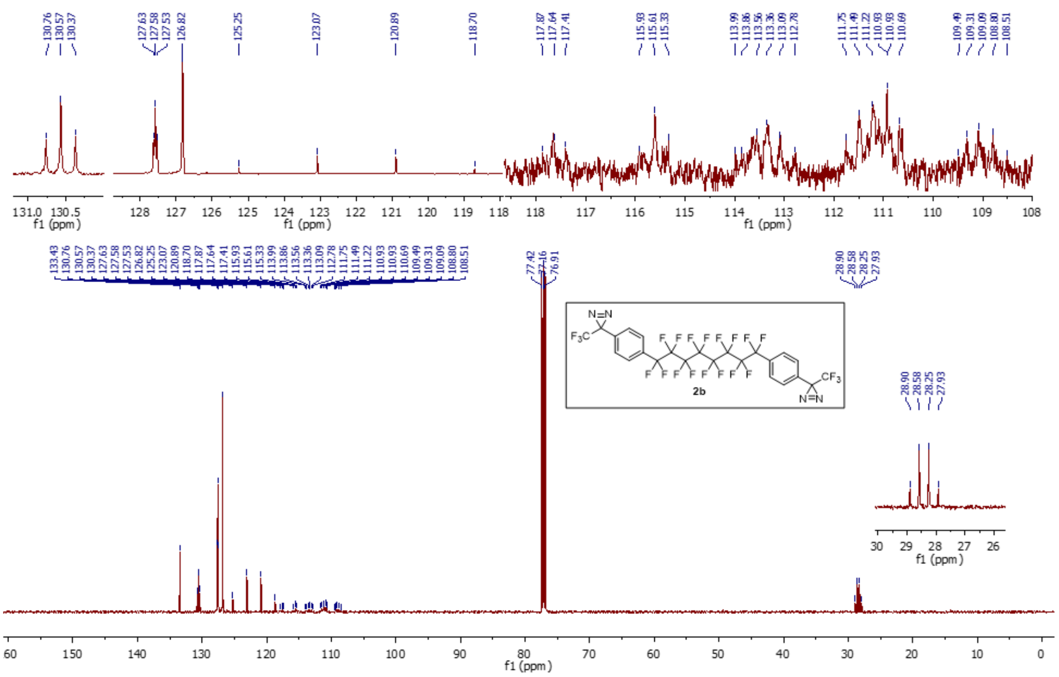

Figure S39. ^{13}C NMR spectrum of **2b** in chloroform-d at 125 MHz.

3. Crosslinking of cyclohexane, as a molecular model for polyethylene

3.1 General procedure for the thermal crosslinking of cyclohexane

Anhydrous cyclohexane taken from a sure-seal bottle was subjected to three freeze/thaw cycles to remove dissolved oxygen. A 10 mM solution of bis-diazirine **1**, **2a** or **2b** in the anhydrous, deoxygenated cyclohexane was prepared in a sealed tube. After capping the tube, the apparatus was heated to 140 °C and stirred at that temperature for 4 h. The reaction mixture was then cooled to room temperature, transferred to a round bottom flask, and concentrated in vacuo. The crude material was dissolved in 10% diethyl ether in pentane (*ca.* 2 mL), loaded onto a column packed with silica gel and eluted with pentane or petroleum ether (see below for comparative NMR data using different purification methods). Several 2–4 mL fractions were collected in 12 x 75 mm test tubes. Fractions that contained the desired product (as determined by $^{1}H/^{19}F$ spectra), were combined and concentrated.

Analytical data for C–H insertion product **9**, prepared using crosslinker **1**, is reported in reference 2. Analytical data for C–H insertion products **10a** and **10b** are provided below.

*3.2 Cyclohexane crosslinking with **2a** to afford **10a***

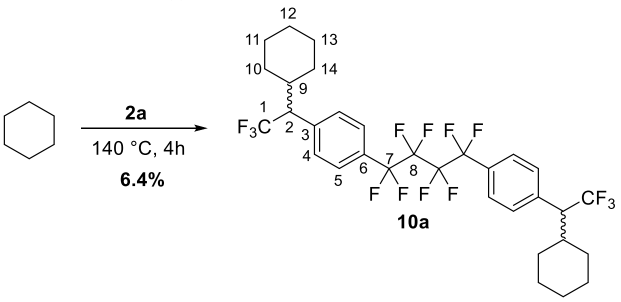

Reaction, workup, and purification were performed as described in the general protocol above, using crosslinker **2a** (0.20 g, 0.35 mmol) in cyclohexane (35 mL). The desired product **10a** (15.3 mg, 6.4%) was isolated as light-yellow solid. ^{1}H NMR (500 MHz, chloroform-d) δ 7.56 (d, J = 8.1 Hz, 4H, H-5), 7.35

(d, J = 8.1 Hz, 4H, H-4), 3.11 (apparent pentet, J = 9.7 Hz, 2H, H-2), 2.05–1.92 (m, 4H, H-9 and cyclohexyl-Hs), 1.78 (d, J = 13.2 Hz, 2H, cyclohexyl-Hs), 1.63 (d, J = 11.5 Hz, 4H, cyclohexyl-Hs), 1.41 (d, J = 12.0 Hz, 2H, cyclohexyl-Hs), 1.22–1.03 (m, 8H, cyclohexyl-Hs), 0.83–0.73 (m, 2H, cyclohexyl-Hs). ^{19}F NMR (471 MHz, chloroform-d) δ –63.19 (s, 6F), –110.53 (t, J = 13.6 Hz, 4F), –121.30 (d, J = 14.3 Hz, 4F). ^{13}C NMR (125 MHz, chloroform-d) δ 139.24 (C-3), 129.43 (C-4), 129.34 (t, $^{2}J_{C\text{-}F}$ = 24 Hz, C-6), 127.18 (t, $^{3}J_{C\text{-}F}$ = 5.6 Hz, C-5), 126.96 (q, $^{1}J_{C\text{-}F}$ = 281.6 Hz, C-1), 118.59–108.68 (m, C-7 and C-8), 56.19 (q, $^{2}J_{C\text{-}F}$ = 25.5 Hz), 38.66 (C-9), 31.55 (cyclohexyl-C), 30.84 (cyclohexyl-C), 26.22 (cyclohexyl-C), 26.13 (cyclohexyl-C), 26.03 (cyclohexyl-C). IR (ATR) υ (cm^{-1}): 2926, 2855, 1620, 1452, 1288, 1254, 1229, 1178, 1158, 1118, 1100, 843, 819. HRMS (FD+) m/z [M] calculated for $C_{32}H_{32}F_{14}^{+}$: 682.2275, found 682.2284.

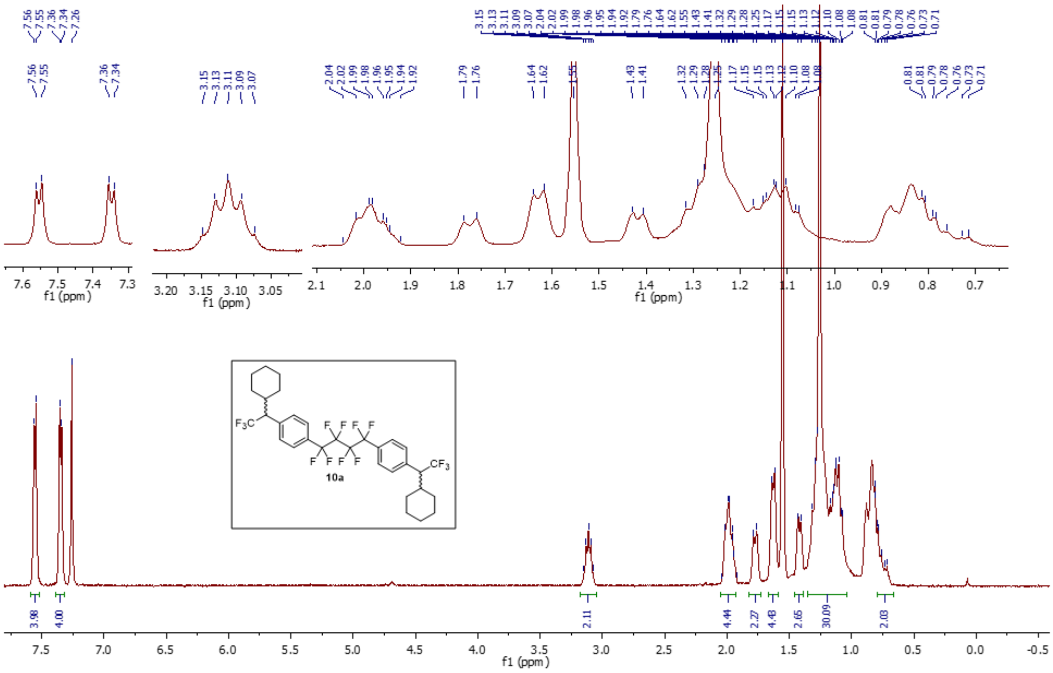

Figure S40a. ^{1}H NMR spectrum of **10a** (purified using pentane) in chloroform-d at 500 MHz. The product is contaminated with a greasy residue from the pentane solvent used for purification.

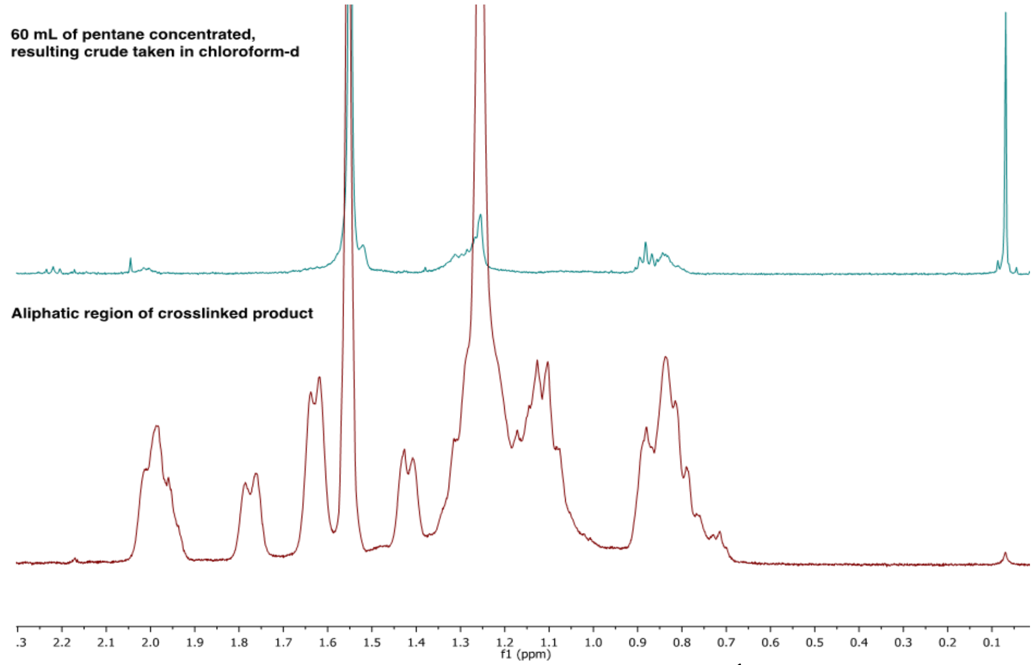

Figure S40b. Comparison of the aliphatic region of the ^{1}H NMR spectrum for **10a** (bottom) to the ^{1}H NMR spectrum of the greasy residue obtained by evaporation of the pentane solvent used for column chromatography (top). Overlapping peaks reveal that the impurity observed in the isolated product is due to the solvent used for purification.

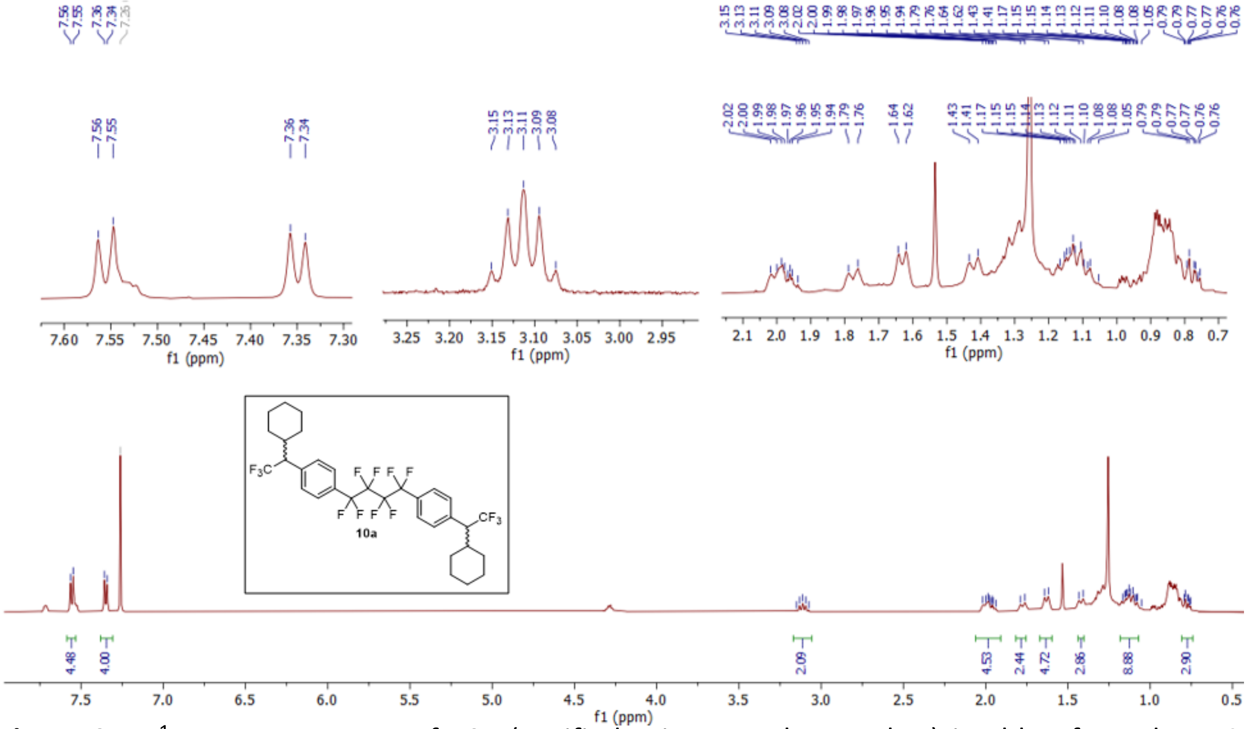

Figure S41. ^{1}H NMR spectrum of **10a** (purified using petroleum ether) in chloroform-d at 500 MHz. Less grease was observed using this purification method, although it was harder to exclude minor impurities.

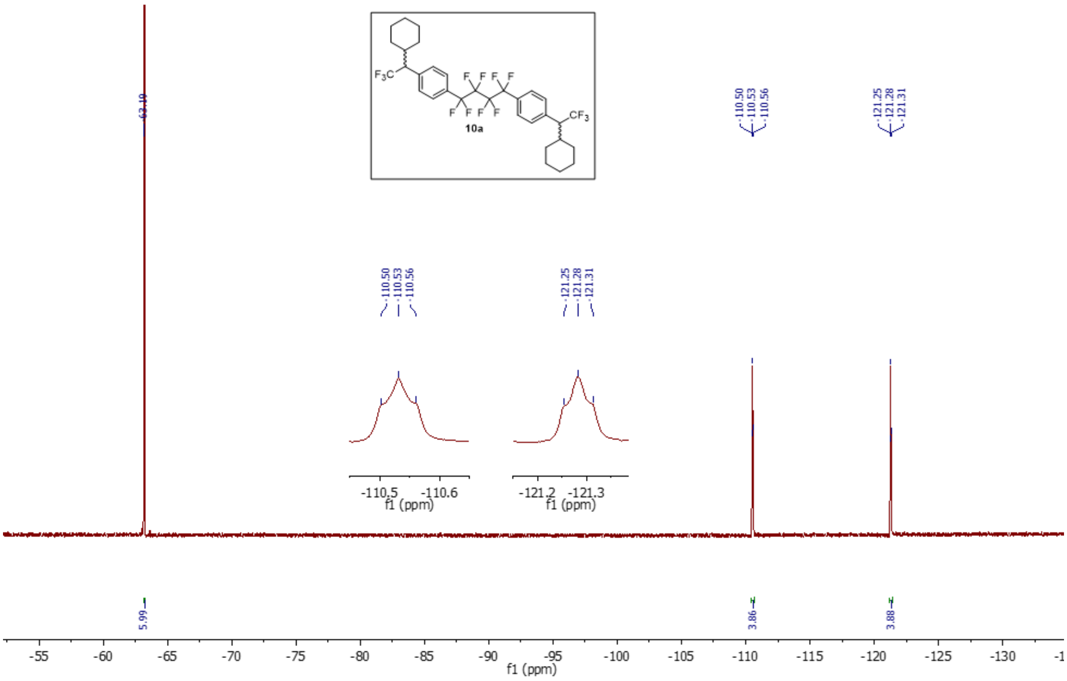

Figure S42. ^{19}F NMR spectrum of **10a** in chloroform-d at 471 MHz.

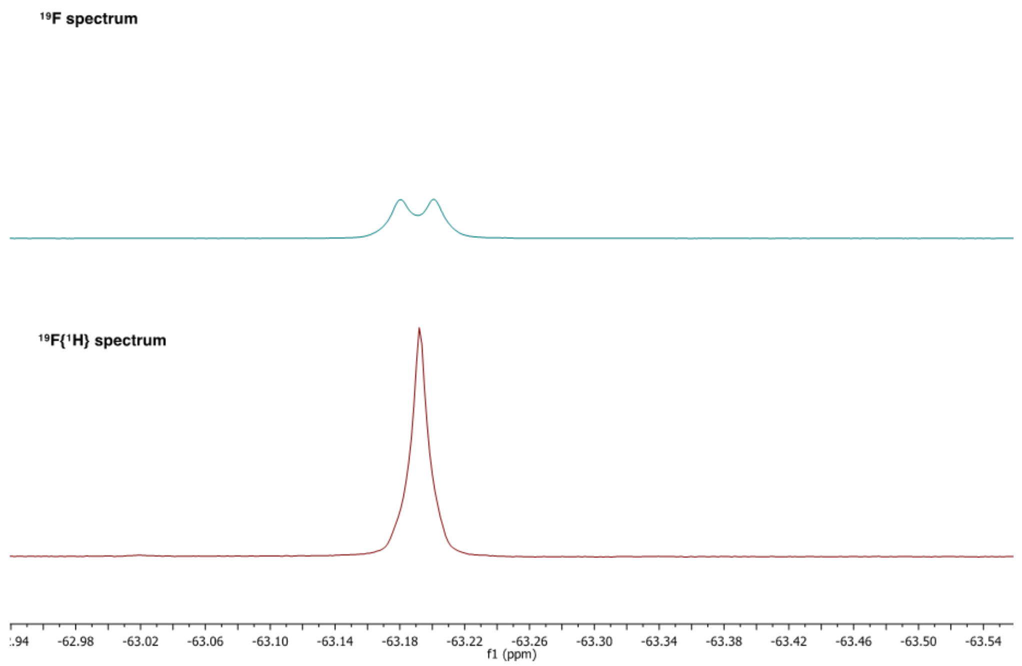

Figure S43. Comparison of the proton coupled (top) and proton decoupled (bottom) ^{19}F NMR spectra for **10a**, confirming that the CF_3 group corresponding to the signal at –63 ppm is adjacent to a methine group that results from C–H insertion into the cyclohexane.

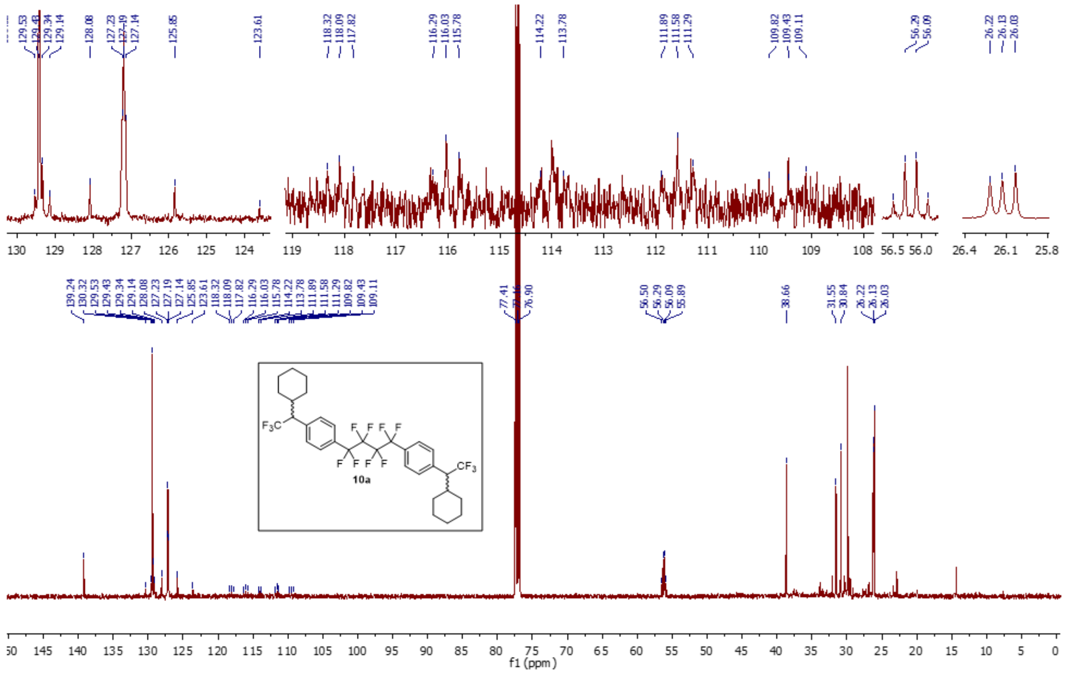

Figure S44. ^{13}C NMR spectrum of **10a** in chloroform-d at 125 MHz.

*3.3 Cyclohexane crosslinking with **2b** to afford **10b***

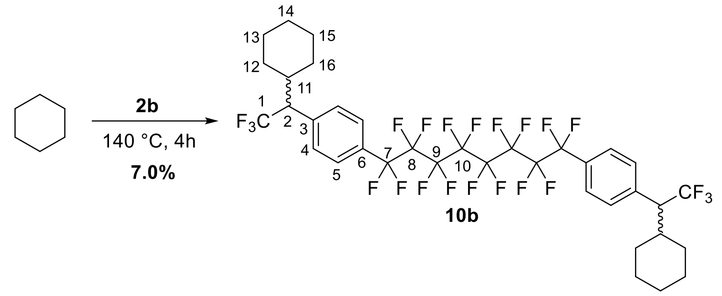

Reaction, workup, and purification were performed as described in the general protocol above, using crosslinker **2a** (0.20 g, 0.26 mmol) in cyclohexane (26 mL). The desired product **10b** (16.1 mg, 7.0%) was isolated as light-yellow solid. ^{1}H NMR (500 MHz, chloroform-d) δ 7.56 (d, *J* = 8.1 Hz, 4H, H-5), 7.37 (d, *J* = 8.1 Hz, 4H, H-4), 3.13 (apparent pentet, *J* = 9.6 Hz, 2H, H-2), 2.04–1.93 (m, 4H, H-11 and H-12), 1.83–1.71 (m, 2H, H-13), 1.64 (d, J = 11.8 Hz, 4H, H-14 and H-15), 1.42 (d, J = 12.4 Hz, 2H, H-16), 1.19–1.07 (m, 8H, H-12, H-13, H-14 and H-15), 0.82–0.73 (m, 2H, H-16). ^{19}F NMR (471 MHz, chloroform-d) δ –63.19 (s, 6F), –110.64 (t, *J* = 14.7 Hz, 4F), –121.27 (broad-s, 4F), –121.76 (broad-s, 8F). ^{13}C NMR (125 MHz, chloroform-d) δ 139.61 (C-3), 129.58 (C-4), 128.73 (t, $^{2}J_{C\text{-}F}$ = 24.5 Hz, C-6), 127.18 (t, $^{3}J_{C\text{-}F}$ = 6.4 Hz, C-5), 126.93 (q, $^{1}J_{C\text{-}F}$ = 281.5 Hz, C-1), 119.46–107.51 (m, C7-C-10), 56.22 (q, *J* = 25.4 Hz, C-2), 38.67 (C-11), 31.56 (cyclohexyl-C), 30.85 (cyclohexyl-C), 26.22 (cyclohexyl-C), 26.13 (cyclohexyl-C), 26.03 (cyclohexyl-C). IR (ATR) υ (cm^{-1}): 2926, 2854, 1620, 1454, 1293, 1254, 1212, 1152, 1137, 1101 836, 818. HRMS (FD+) m/z [M] calculated for $C_{36}H_{32}F_{22}^{+}$: 882.2147, found: 882.2159.

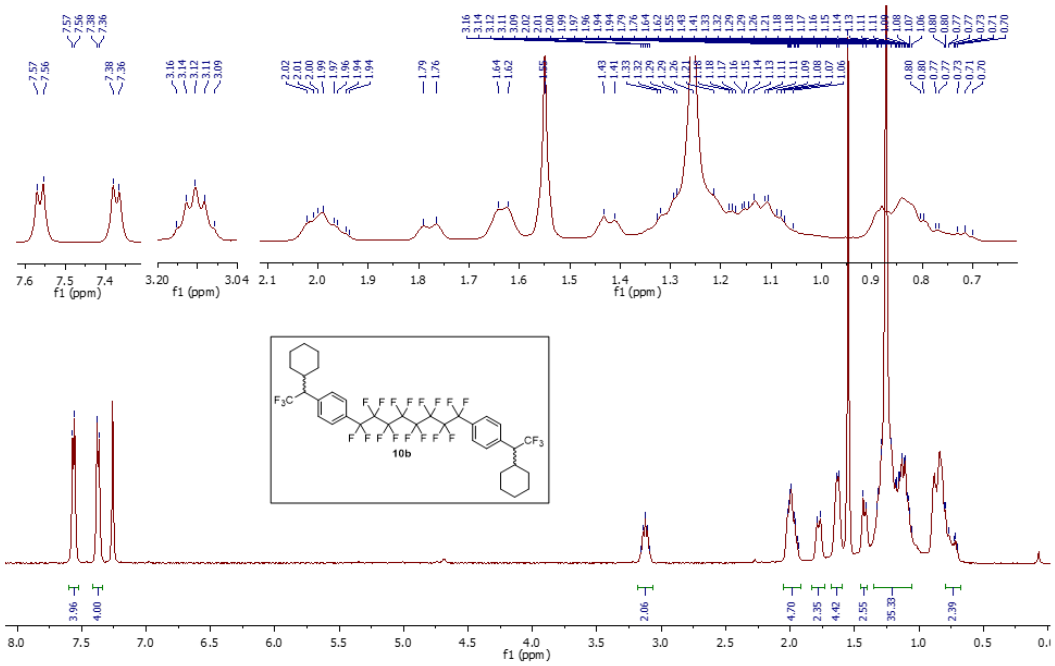

Figure S45a. ^{1}H NMR spectrum of **10b** (purified using pentane) in chloroform-d at 500 MHz. The product is contaminated with a greasy residue from the pentane solvent used for purification.

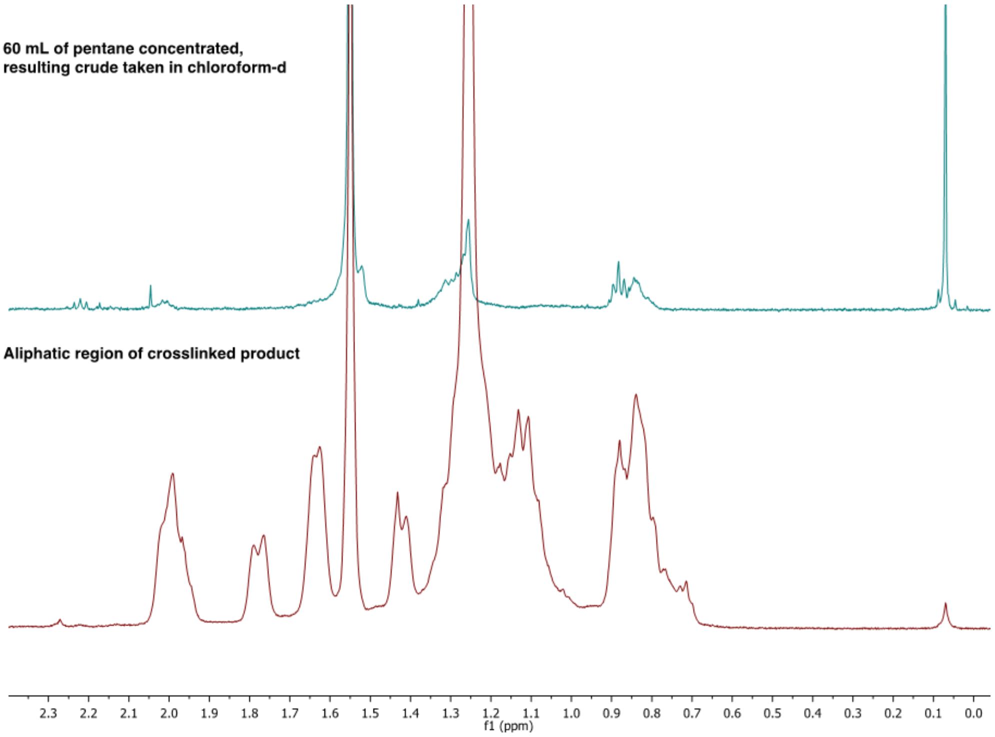

Figure S45b. Comparison of the aliphatic region of the ^{1}H NMR spectrum for **10b** (bottom) to the ^{1}H NMR spectrum of the greasy residue obtained by evaporation of the pentane solvent used for column chromatography (top). Overlapping peaks reveal that the impurity observed in the isolated product is due to the solvent used for purification.

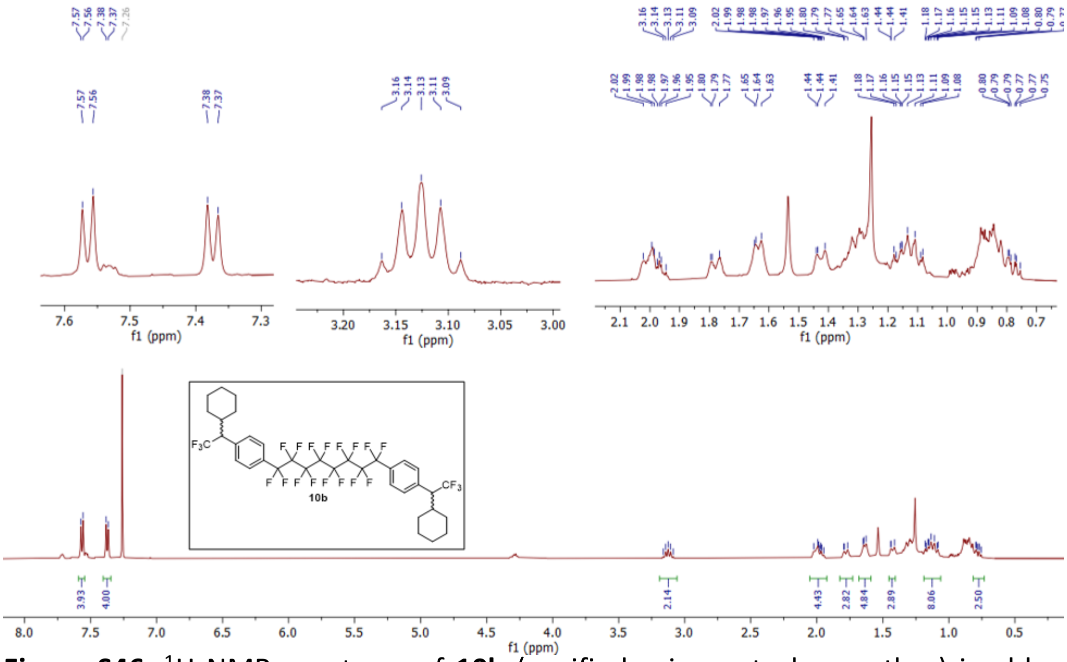

Figure S46. ^{1}H NMR spectrum of **10b** (purified using petroleum ether) in chloroform-d at 500 MHz. Less grease was observed using this purification method, although it was harder to exclude minor impurities.

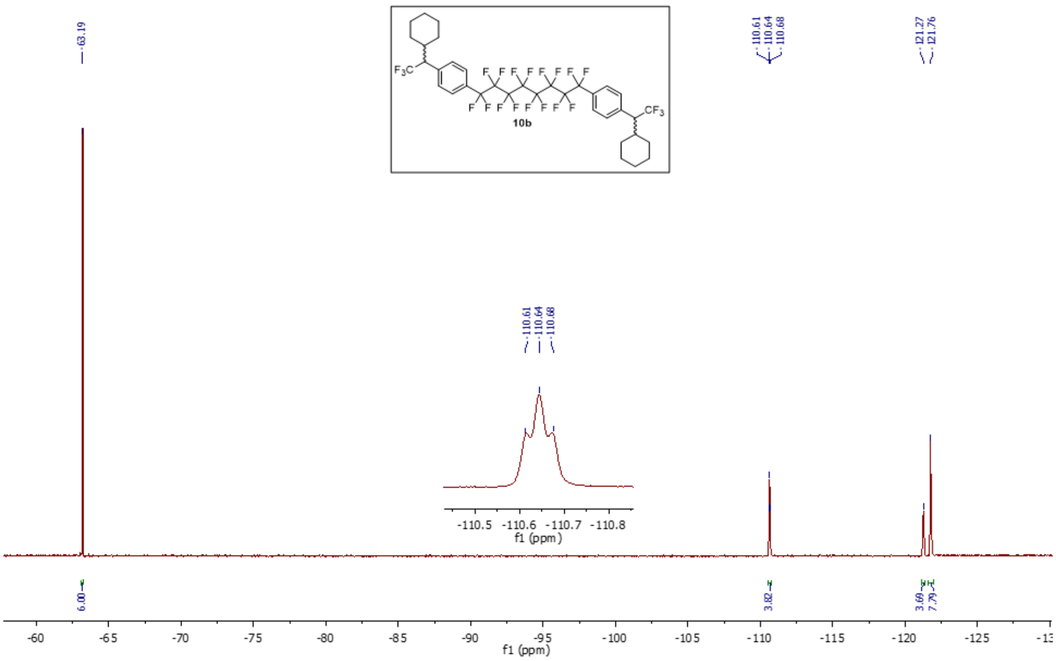

Figure S47. ^{19}F NMR spectrum of **10b** in chloroform-d at 471 MHz.

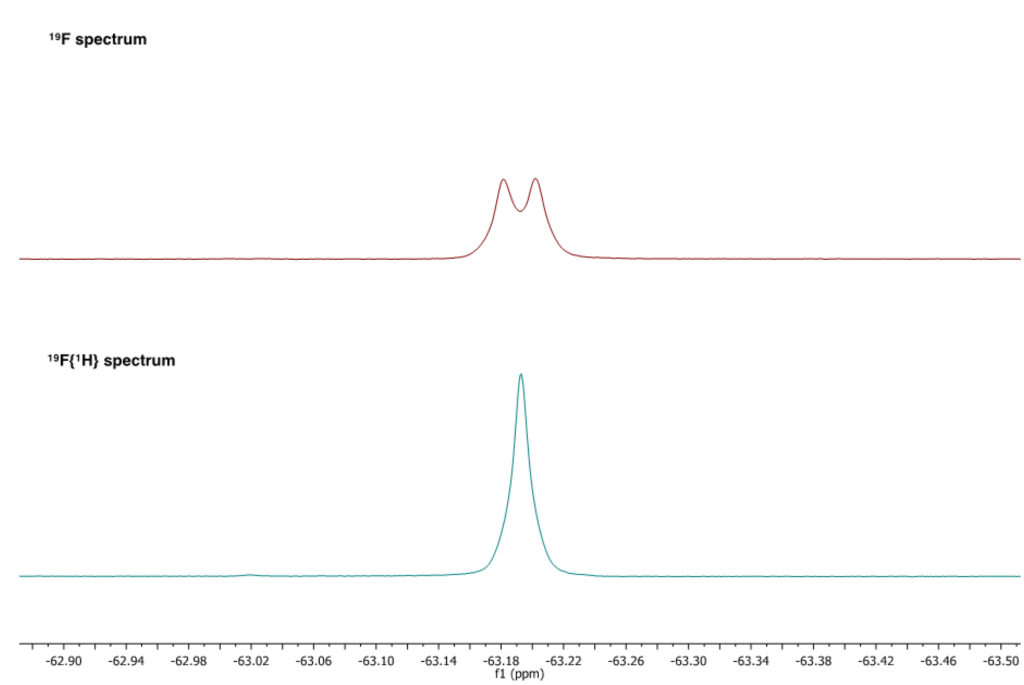

Figure S48. Comparison of the proton coupled (top) and proton decoupled (bottom) ^{19}F NMR spectra for **10b**, confirming that the CF_3 group corresponding to the signal at –63 ppm is adjacent to a methine group that results from C–H insertion into the cyclohexane.

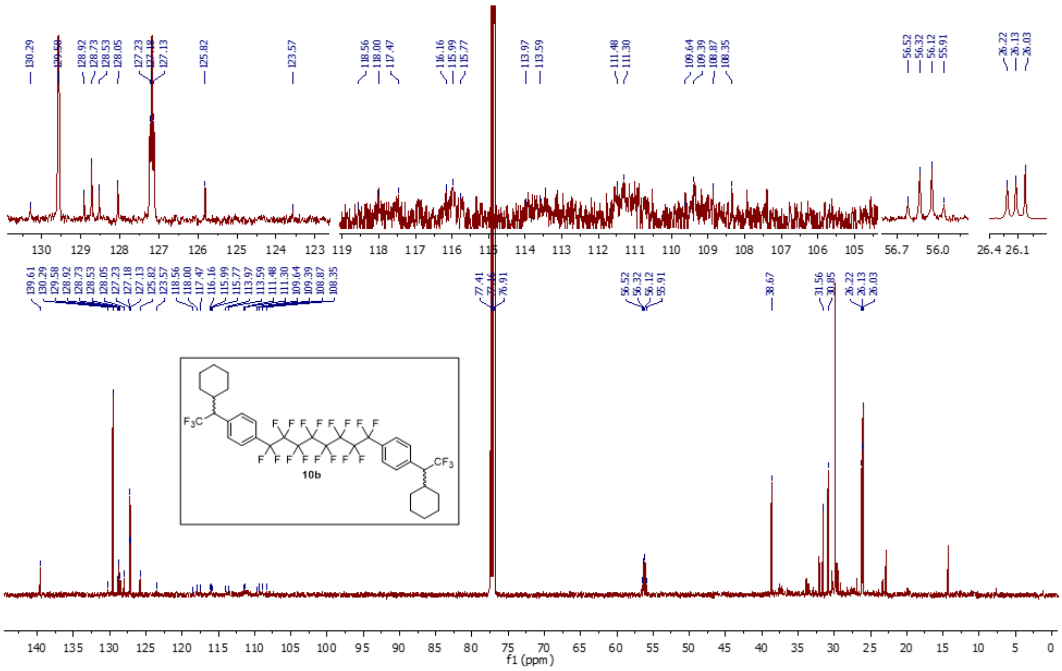

Figure S49. ^{13}C NMR spectrum of **10b** in chloroform-d at 125 MHz.

4. Assessment of Thermal Properties

4.1 Differential scanning calorimetry (DSC)

A sample of the diazirine to be analyzed (2–9 mg) was placed in a Tzero aluminum pan and sealed with a matching lid. The lid to the pan was pierced with a small pinhole to allow evolution of nitrogen gas. The pan was placed in the oven of the calorimeter, and heated from 40 °C to 200 °C at a rate of 5 °C/min, with an identical empty pan as a reference. The oven was constantly flushed by a 50 mL/min flow of nitrogen gas during the analysis.

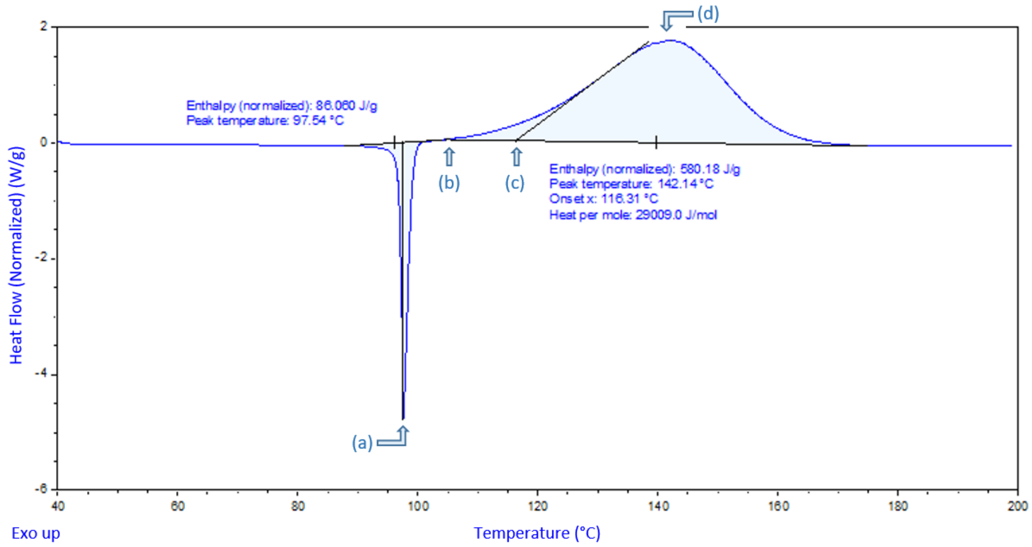

Figure S50. Representative DSC trace for crosslinker **2a**. (a) Melting temperature. (b) Start of diazirine activation. (c) Numerically determined T_{onset} value, calculated by extrapolating the tangent of the upward slope to the baseline. (d) T_{max} value. Refer to Table S1 for tabulated data.

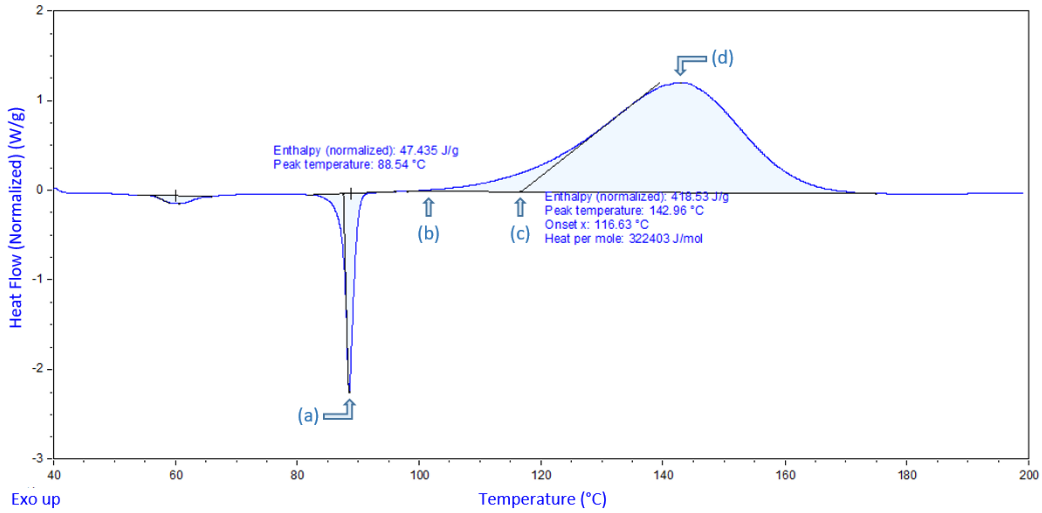

Figure S51. Representative DSC trace for crosslinker **2b**. (a) Melting temperature. (b) Start of diazirine activation. (c) Numerically determined T_{onset} value, calculated by extrapolating the tangent of the upward slope to the baseline. (d) T_{max} value. Refer to Table S1 for tabulated data.

4.2 Assessment of explosivity

Shock sensitivity (SS) and propensity for explosive propagation (EP) were calculated according to Yoshida's methods[3], according to the following formulae:

$$\text{Shock sensitivity} = \log (Q_{DSC}) - 0.72 \times \log (T_{onset} - 25) - 0.98$$

$$\text{Explosive propagation} = \log (Q_{DSC}) - 0.38 \times \log (T_{onset} - 25) - 1.67$$

where Q_{DSC} is the enthalpy of nitrogen release (measured in cal/g) and T_{onset} is the onset temperature for diazirine activation (measured by extrapolation of the tangent of the DSC curve, as shown above, and reported in ºC). A material is considered likely to be explosive if shock sensitivity and/or explosive propagation values are positive.

Table S1. Thermal Properties for Synthesized Crosslinkers

Crosslinker:	**1**	**2a**	**2b**
Melting transition (°C)	34[a]	97.7 ± 0.2	88.7 ± 0.2
T_{onset} (°C)	113.1 ± 0.2	116.6 ± 0.5	116.8 ± 0.3
T_{max} (°C)	137.3 ± 0.9	141.8 ± 0.9	142.8 ± 0.5
Q_{DSC} (J/g)	646 ± 20	587 ± 15	430 ± 30
Q_{DSC} (kJ/mol)	335 ± 10	335 ± 9	331 ± 23
Shock sensitivity (SS)	–0.19	–0.24	–0.38
Explosive propagation (EP)	–0.22	–0.27	–0.40

[a] Melting point for crosslinker **1** taken from reference 2. The other data for compound **1** were freshly determined by DSC, so that accurate comparisons of Yoshida correlations could be made to the data from compounds **2a** and **2b**.

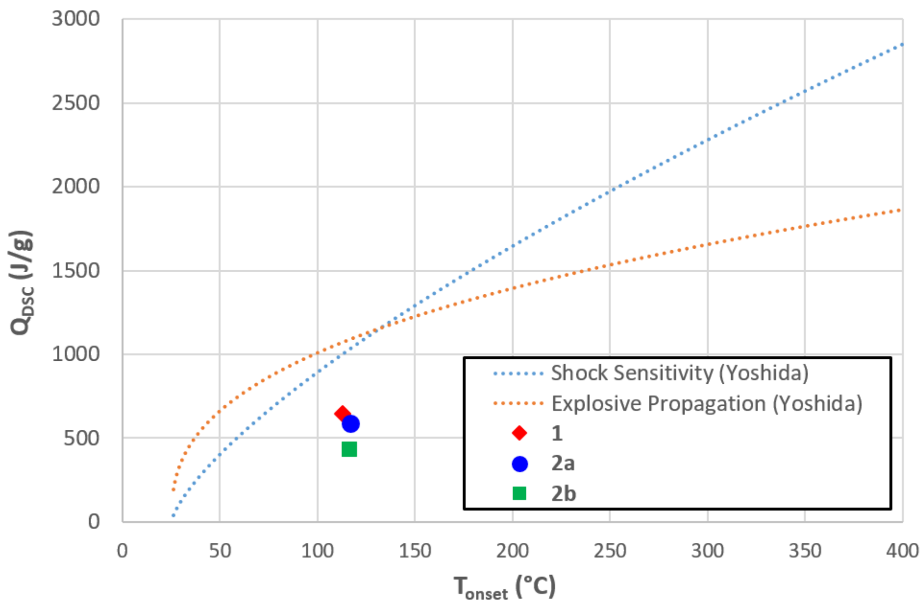

Figure S52. Yoshida correlations showing that all three crosslinkers are below the thresholds for shock sensitivity and explosive propagation.

5. Crosslinking of PDMS

*5.1 Experimental procedure for comparison of crosslinking effects of **1**, **2a** and **2b** analyzed by GPC*

A series of samples was prepared by mixing low viscosity (25 cSt) PDMS (200 mg) with various amounts of **1**, **2a** or **2b** (see Table S2) in glass vials. DCM (200 µL) was added to each vial, following which all vials were mixed for 15 seconds on a vortex mixer and left to dry in the fume hood overnight. THF (200 µL) was added to each vial, following which all vials were mixed for 15 seconds on a vortex mixer and left to dry in the fume hood overnight. The contents of the vials were further dried by heating the vials at 45–50 °C in a heat block for 2 hours. The contents of the vials were further dried by placing them under high vacuum after mixing them for 15 seconds on a vortex mixer at the highest speed (to disrupt their gel structure). The removal of solvents was monitored by measuring the weights of each vial over the whole process.

The samples were placed in a heat block (appropriately chosen to snugly fit each vial) and heated at 110 °C for 16 hours. This induced crosslinking via thermal activation. See Figure S53 for pictures of the samples before and after crosslinking. Once cooled, each sample was homogenized (vortex mixer or manually) and 10 mg was dissolved in 1 mL THF. The solutions were then filtered through a 0.45 µm PTFE filter directly into vials for GPC analysis. An extra control sample was prepared by dissolving 10 mg of PDMS 25 cSt into 1 mL of THF and filtering the resulting solution through a 0.45 µm PTFE filter. All filtered samples were clear solutions. A blank THF sample was analyzed prior to each GPC run to ensure that the system was free from contamination.

All samples were analyzed using a Malvern OMNISEC gel permeation chromatograph equipped with an automatic sampler, a pump, an injector, an inline degasser, and a column oven (30 °C). The elution columns T3000 and T5000 from Viscotek were used. Detection was conducted by means of viscometry and differential refractive index. HPLC-grade tetrahydrofuran (Thermo Fisher) spiked with 1 vol% triethylamine was used as the eluent, with a flow rate of 1.0 mL/min. The relevant portion of the chromatograms obtained from the viscometer and refractive index detector are displayed in Figure S54. The retention times for the major peak of each sample are reported in Table S3. When plotted against crosslinker loading, the retention times show comparable behavior of the three crosslinkers. See Figure S55 for a comparison of the trends observed by viscometric and refractive index detection.

Table S2. Composition and Concentration of Samples Prepared for the PDMS Crosslinking Experiment

Vial	Crosslinker	MW (g/mol)	Loading (µmol/g)	PDMS (mg)	Crosslinker (µmol)	Crosslinker (mg)	Loading (wt%)	Loading (mol%)
A1	**1**	520.28	0	200	0	0.0	0.0%	0.0%
A2		520.28	20	200	4	2.1	1.0%	1.1%
A3		520.28	40	200	8	4.2	2.1%	2.2%
A4		520.28	70	200	14	7.3	3.6%	3.9%
A5		520.28	100	200	20	10.4	5.2%	5.5%
A6		520.28	130	200	26	13.5	6.8%	7.2%
A7		520.28	160	200	32	16.6	8.3%	8.8%
A8		520.28	200	200	40	20.8	10.4%	11.0%
D2	**2a**	570.29	20	200	4	2.3	1.1%	1.1%
D3		570.29	40	200	8	4.6	2.3%	2.2%
D4		570.29	70	200	14	8.0	4.0%	3.9%
D5		570.29	100	200	20	11.4	5.7%	5.5%
D6		570.29	130	200	26	14.8	7.4%	7.2%
D7		570.29	160	200	32	18.2	9.1%	8.8%
D8		570.29	200	200	40	22.8	11.4%	11.0%
E2	**2b**	770.32	20	200	4	3.1	1.5%	1.1%
E3		770.32	40	200	8	6.2	3.1%	2.2%
E4		770.32	70	200	14	10.8	5.4%	3.9%
E5		770.32	100	200	20	15.4	7.7%	5.5%
E6		770.32	130	200	26	20.0	10.0%	7.2%
E7		770.32	160	200	32	24.7	12.3%	8.8%
E8		770.32	200	200	40	30.8	15.4%	11.0%

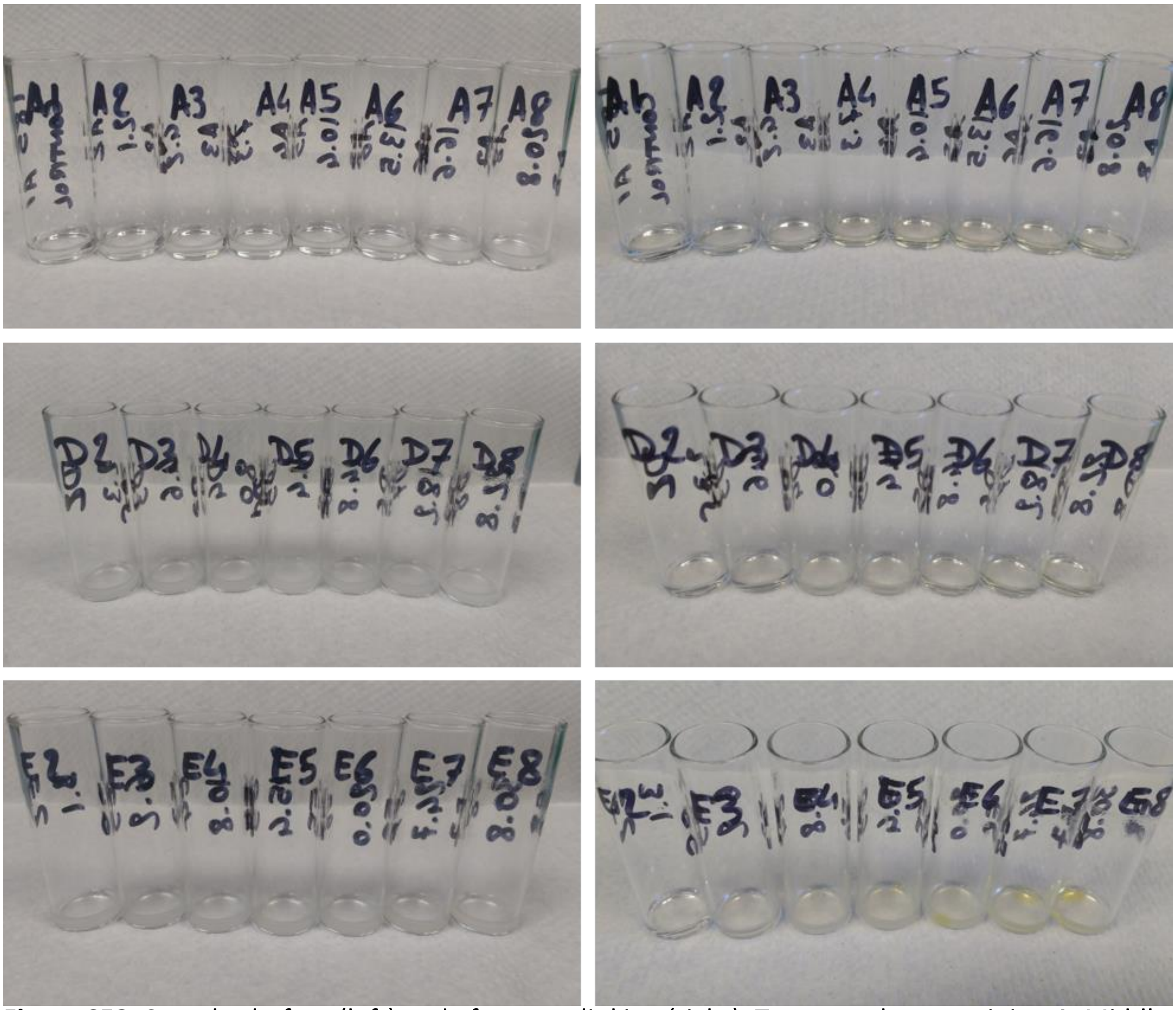

Figure S53. Samples before (left) and after crosslinking (right). Top: samples containing **1**; Middle: samples containing **2a**; Bottom: samples containing **2b**.

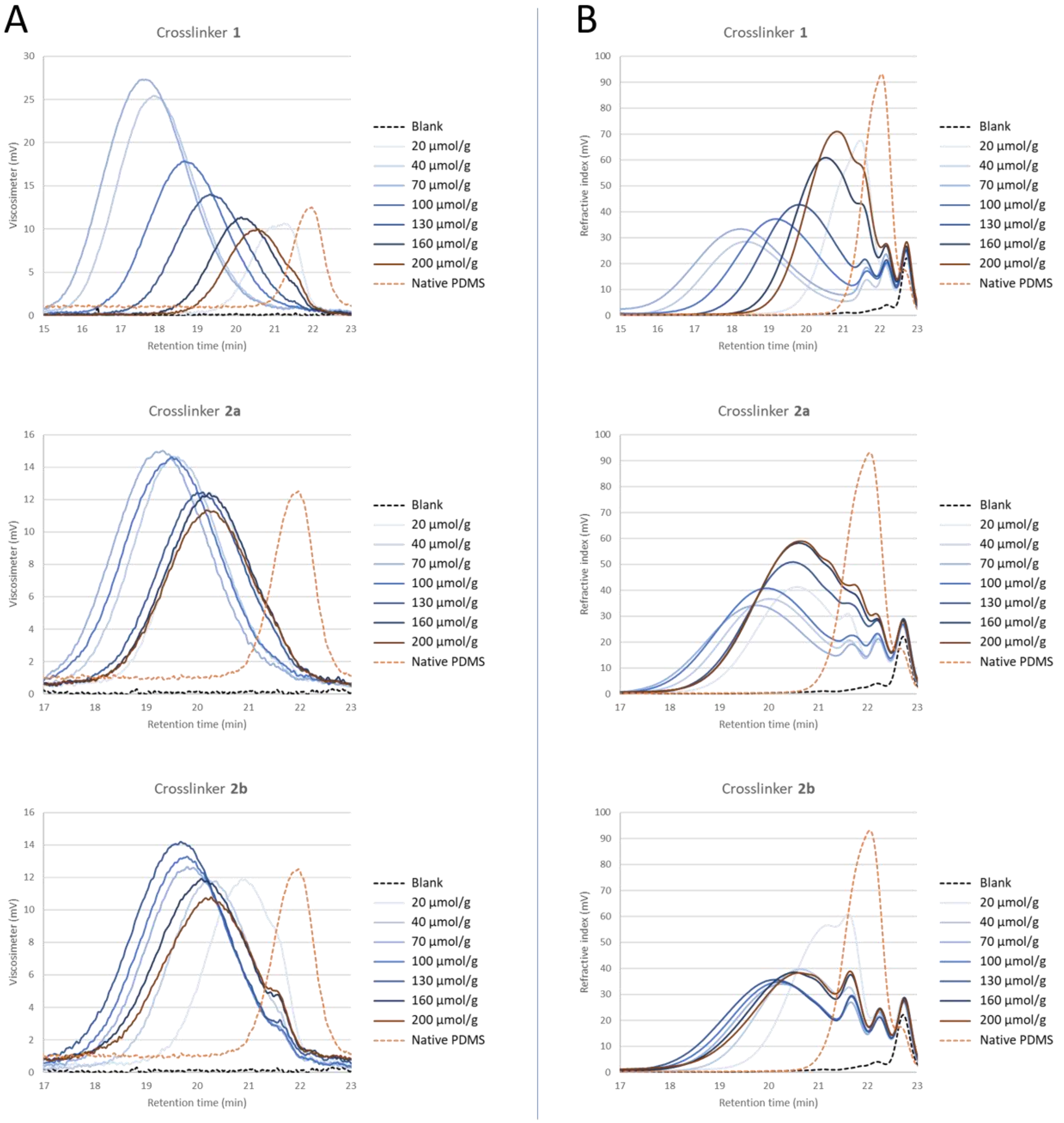

Figure S54. GPC chromatograms showing a change of retention time depending on the concentration of crosslinker used. A: Viscometric detection. B: Refractive index detection.

Table S3. Retention Times for Major Peak in GPC Chromatogram[a]

Viscometric Detection				Refractive Index Detection			
Loading (µmol/g)	Crosslinker			Loading (µmol/g)	Crosslinker		
	1	2a	2b		1	2a	2b
**0**	21.97	21.97	21.97	**0**	22.05	22.05	22.05
**20**	21.25	20.24	20.86	**20**	21.47	20.60	21.61
**40**	17.86	19.54	20.23	**40**	18.36	20.00	20.61
**70**	17.57	19.33	19.81	**70**	18.25	19.76	20.28
**100**	18.67	19.50	19.80	**100**	19.19	19.95	20.17
**130**	19.33	20.11	19.68	**130**	19.84	20.50	20.11
**160**	20.14	20.22	20.08	**160**	20.56	20.60	20.55
**200**	20.58	20.17	20.27	**200**	20.83	20.65	21.65

[a] Retention times in minutes, as a function of crosslinker and concentration.

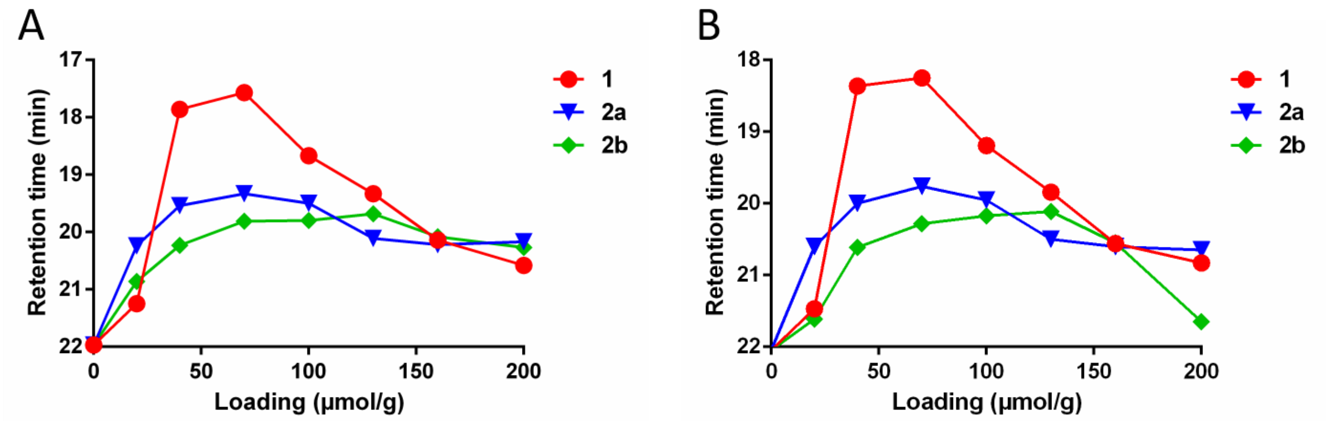

Figure S55. Comparison of PDMS crosslinking efficacy for **1**, **2a** and **2b**, using both viscometric detection (A) and refractive index detection (B). The retention time at which the largest viscosity signal was observed for each experiment was then plotted against crosslinker concentration. The resulting curves confirm that all three reagents are capable of crosslinking PDMS.

6. Adhesion Testing

6.1 Preparation of adhered HDPE–HDPE samples

Pairs of 4”x1”x¼” bars of HDPE (Quadrant or Röchling Engineering Plastics) were treated with 1, 5 or 10 mg of crosslinkers **1**, **2a**, or **2b** and the strength of adhesive bonding was determined in accordance with ASTM D3163. After cutting the HDPE sheet into bars of the appropriate size, the edges were scraped to smoothness using an exacto knife and the bars were wiped with Kimwipes to remove dust/plastic particles. The crosslinkers were suspended in 120 µL of diethyl ether and were deposited onto the 1”x0.5” overlap zone using a pipette. The solvent was allowed to evaporate, and then each pair of bars was held together with binder clamps and placed into an oven pre-heated to 115 °C. After 15 h, the samples were removed from the oven, cooled to room temperature, and challenged on a lap-shear experiment.

Negative (vehicle) controls were prepared in an identical manner, except that 120 µL of pure diethyl ether was added to the bars in place of the crosslinker solution described above. Vehicle control samples were incubated at the same temperature (115 °C) as the other samples, for an identical length of time.

Positive (cyanoacrylate) controls were prepared by adding *ca.* 10 mg of commercial super glue (1 drop) directly to the overlap zone of an HDPE lap-shear sample, with no solvent. Superglue control samples were not heated prior to analysis, but were held together using the same binder clamps as described above to allow sufficient curing to take place.

6.2 Preparation of adhered HDPE–PP and UHMWPE–PP samples

Pairs of 4”x1”x¼” bars of HDPE (Quadrant) and polypropylene (PP, Röchling Engineering Plastics), or else pairs of ultra-high molecular weight polyethylene (UHMWPE, Röchling Engineering Plastics) and polypropylene (PP, Röchling Engineering Plastics) were treated with 5 mg of crosslinkers **1**, **2a**, or **2b**. The remaining preparation was the same as described in section 6.1.

6.3 Preparation of adhered PMMA–PMMA, PP–PP and PC–PC samples

Pairs of 4”x1”x¼” bars of poly(methyl methacrylate) (PMMA, Acrylite FF), PP (Röchling), or polycarbonate (PC, Lexan) were treated with 5 mg of crosslinkers **1**, **2a**, or **2b**. The remaining preparation was the same as described in section 6.1, except that the activation temperature used for the PMMA–PMMA samples was 105–110 °C, while the activation temperature used for the PP–PP and PC–PC samples was 125–135 °C

6.4 Lap-shear test

The two trailing ends of the adhered HDPE samples prepared as described above were clamped in a universal testing system (Instron, Series 5969) and pulled apart at a rate of 3 mm/min until breakage of the bond. The maximum force was recorded for each specimen, and plots of load vs. linear extension were used to determine mechanical compliance. Representative force–displacement curves can be found in Figure S56.

Adhesion strength (MPa) was calculated as the amount of shear force (Newtons) needed to break the sample, divided by the overlap area (in mm^2). Plots of adhesion strength vs. crosslinker type for aged HDPE samples (Quadrant) and new HDPE samples (Röchling) can be found in Figure 3b and Figure 3a, respectively. Corresponding plots of adhesion energy density (determined by dividing the area under the force–displacement curve (in mJ) by overlap area (in mm^2) can be found in Figure S57. Plots of adhesion strength vs. crosslinker type for new Quadrant HDPE samples, as well as for polypropylene–polypropylene bonding, HDPE–polypropylene bonding, and ultra-high molecular weight polyethylene (UHMWPE)–polypropylene bonding, can be found in Figure S58. Plots of adhesion strength vs. crosslinker type for PMMA–PMMA bonding and polycarbonate–polycarbonate bonding can be found in Figure S59.

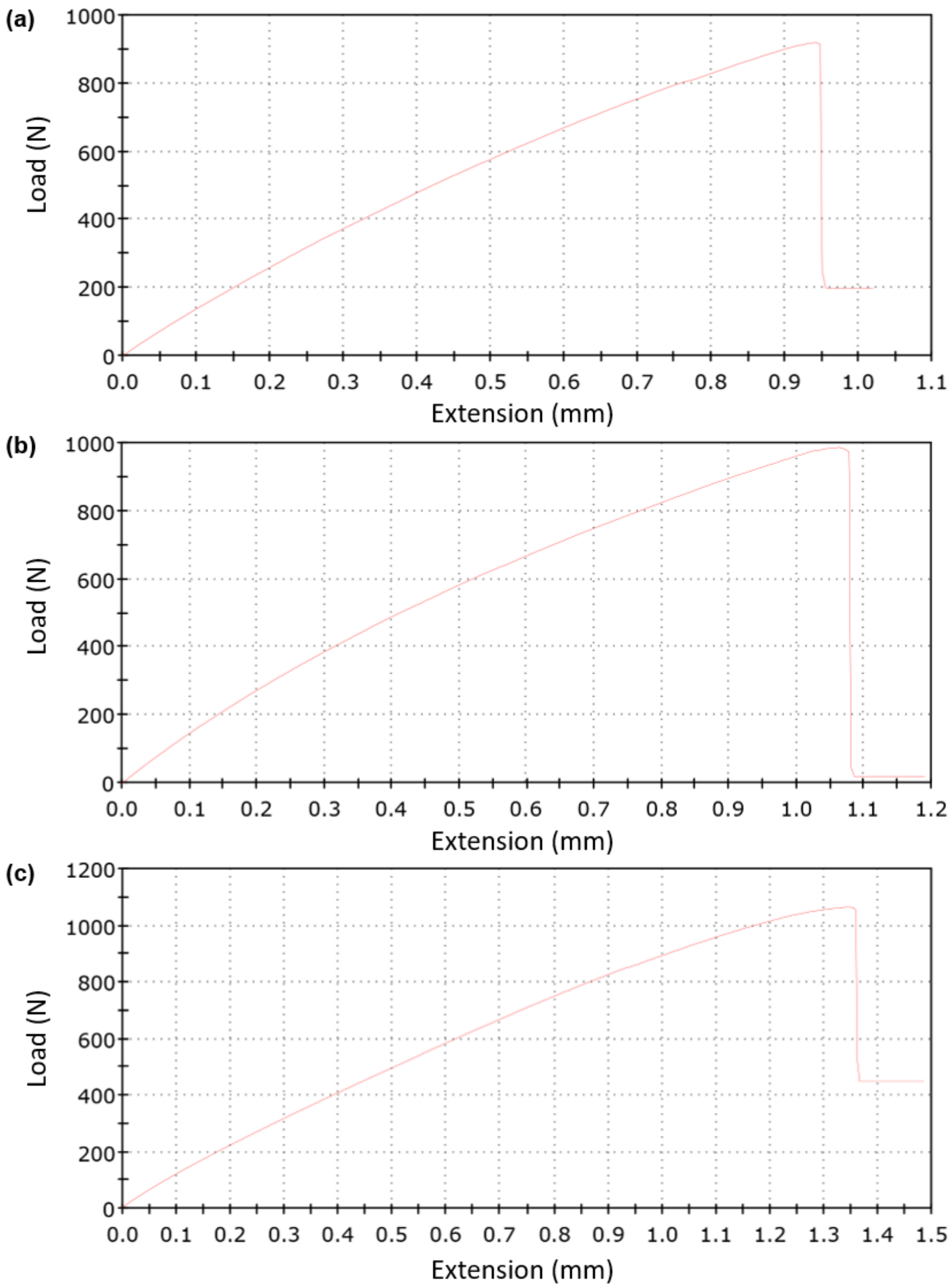

Figure S56. Representative force–extension curves for Quadrant HDPE bars (>6 months old) treated with (a) 1 mg **1**; (b) 1 mg **2a**; and (c) 1 mg **2b**.

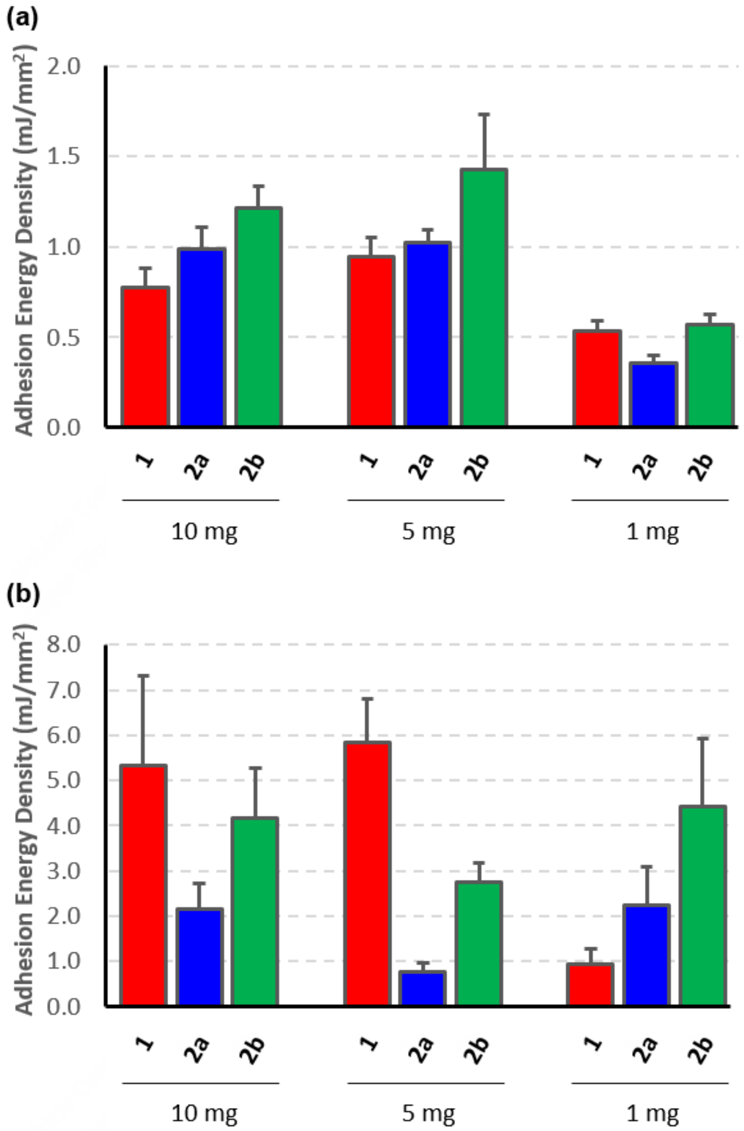

Figure S57. Lap-shear data for crosslinkers **1**, **2a** and **2b**, expressed as adhesion energy density. (a) Adhesion energy density data collected using new HDPE bars (Röchling). (b) Adhesion energy density data collected using old HDPE bars (Quadrant). For the corresponding plots of adhesion strength (in units of MPa) refer to Figure 3. Error bars indicate standard error in all cases.

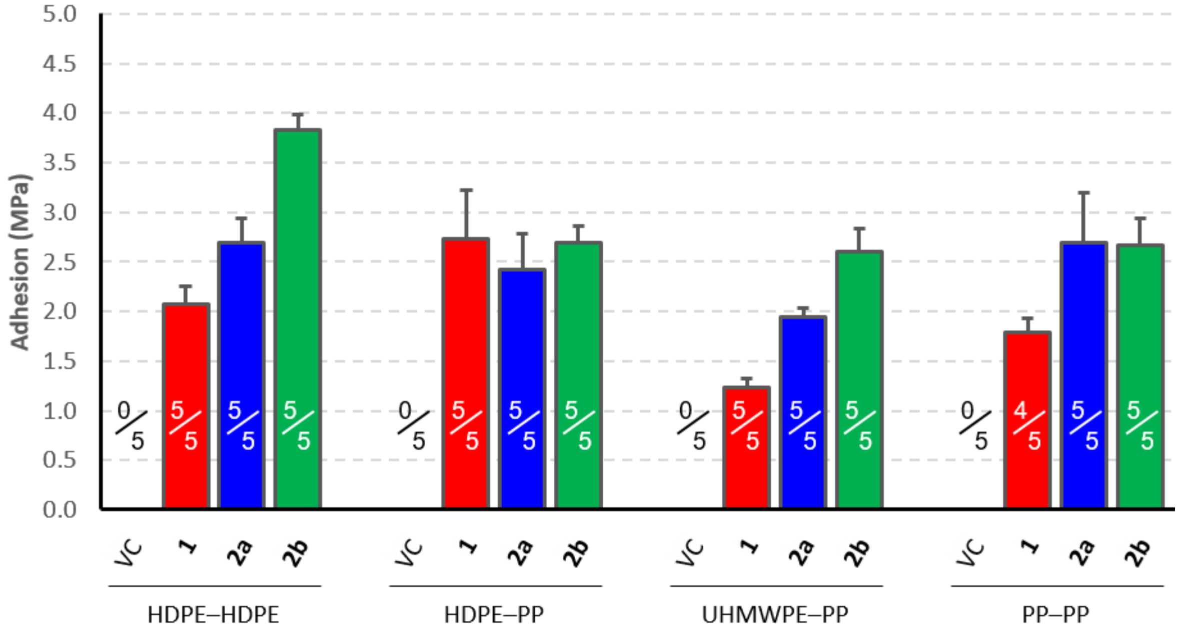

Figure S58. Lap-shear data indicating adhesion strength for mixed-material bonding, using 5 mg of crosslinkers **1**, **2a** and **2b**. VC = vehicle control. HDPE = new high density polyethylene samples from Quadrant. PP = polypropylene. UHMWPE = ultra-high molecular weight polyethylene. Fractions indicate the number of samples that survived clamping into the instrument. Error bars indicate standard error in all cases.

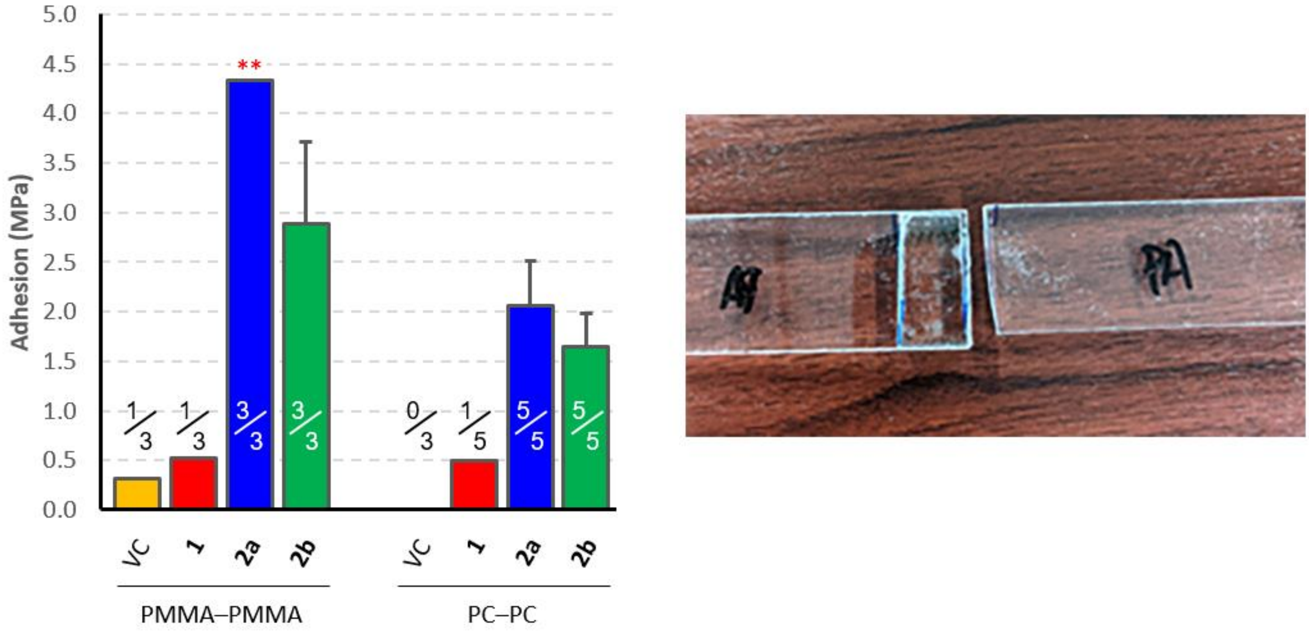

Figure S59. Lap-shear data indicating adhesion strength for polar polymer samples, using 5 mg of crosslinkers **1**, **2a** and **2b**. VC = vehicle control. PMMA = poly(methyl methacrylate). PC = polycarbonate. Fractions indicate the number of samples that survived clamping into the instrument. Error bars indicate standard error in all cases. ** indicates stock break failure for all three PMMA–PMMA lap-shear samples treated with **2a** (see inset figure). This indicates that the adhesive bond was stronger than the substrate material.

6.5 Analysis of lap-shear samples following breakage

After conducting the lap-shear experiments, HDPE bars were examined under a microscope to identify any visible features within the bonded region. For most samples, no visible difference was observed between the bonded and unbonded surfaces (Figure S60).

We therefore used an optical profilometer (Bruker Contour GT 3D Optical microscope) to examine the bonded and unbonded surfaces to gain insight into the mode of failure during the lap-shear testing. Figure S61A shows two images (from separate areas of the same bar) for a typical surface texture of Röchling HDPE bars outside of the bonded regions. Craters of *ca.* 1 µM in depth are visible, as are scratches resulting from manufacture, handling, and shipping.

Typical surface textures of the bonded regions (for both the top and bottom bars from the lap-shear sandwich) of the same Röchling HDPE bar treated with 1 mg of crosslinker **2b** are shown in Figure S61B and S61C. Once again, two separate images were acquired within each area of interest.

The bonded regions on both sides of the lap-shear samples display the same craters and scratches that were visible within the control region, but also display evidence of surface reconfiguration (scaling visible in the inverted-color images), which evidently arises due to the shear loading exerted on the bonded regions during the testing. The similarity between the surface texture of the two sides of the lap-shear sample (by comparison with the untreated control) indicates that similar mechanical stresses were experienced by both bars at the time of rupture, which is consistent with a very broad definition of cohesion failure.[4]

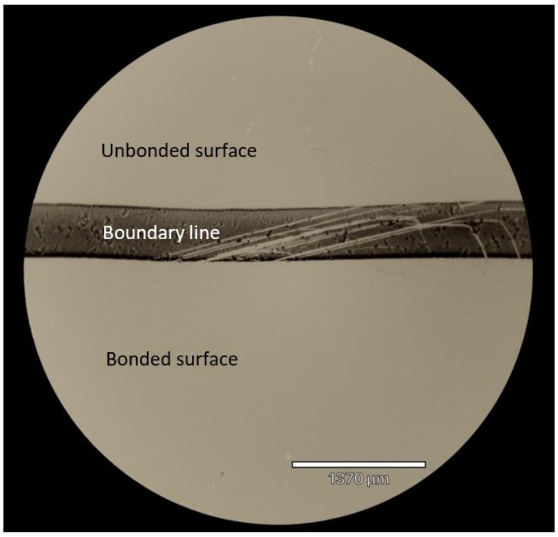

Figure S60. Optical microscope image of a Röchling HDPE bar, following lap-shear testing. The image was acquired sing a Rebel Hybrid microscope, with a 4X magnification.

A. Untreated Rochling HDPE bar

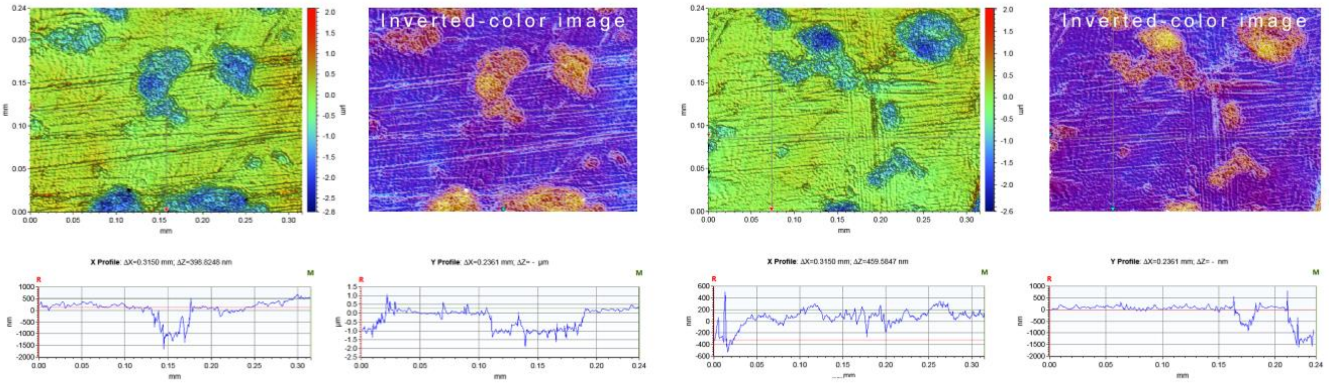

B. Rochling HDPE bar after application of 1 mg **2b** and lap-shear rupture (side 1)

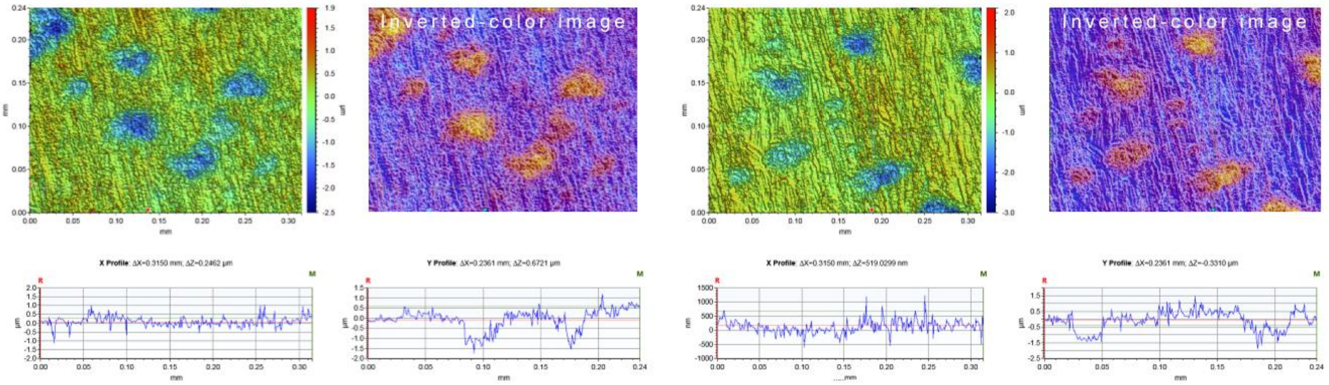

C. Rochling HDPE bar after application of 1 mg **2b** and lap-shear rupture (side 2)

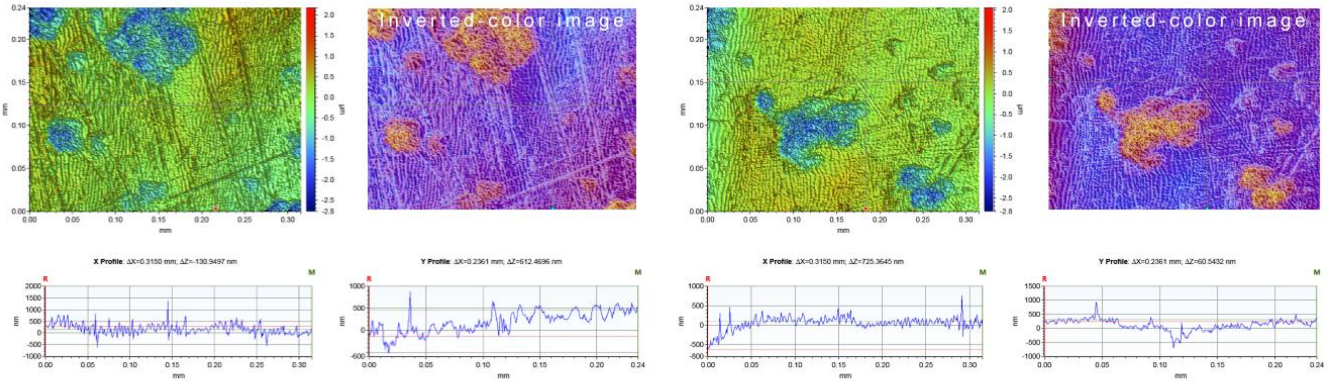

Figure S61. Optical profilometry measurements of a Röchling HDPE bar, following lap-shear testing. Two control regions are shown, together with four regions from within the bonded area (two from each bar that constituted the lap-shear sandwich). For each acquired image, a corresponding inverted-color image is provided that reveals the presence of surface restructuring within the bonded regions, relative to the control regions.

Supplemental Results and Discussion

1. In Silico Evaluation of Carbene Additions to Arenes vs. Perfluoroarenes

Carbenes may react with benzene or its derivatives through mechanisms shown in Figure S62. We used a reduced model for the carbene because the saturated tether connecting the two carbene units in the crosslinker ensures that the carbene centers are electronically isolated. Depending on the electronic state of the carbene, addition or insertion products may be obtained via different pathways. The computational exploration of these pathways indicated that concerted singlet insertion into the hexafluorobenzene C–F bond (Figure S62(a)) and the direct abstraction of an F atom from C_6F_6 (Figure S62(d)) have much higher barriers than the addition pathways (Figure S62(b) and Figure S62(c)). The results of the calculations are presented in Table S4. We were unable to locate a transition state associated with direct insertion to the hexafluorobenzene C–F bond, but its higher bond strength suggests to us that this process will have a higher barrier than that associated with C–H insertion.

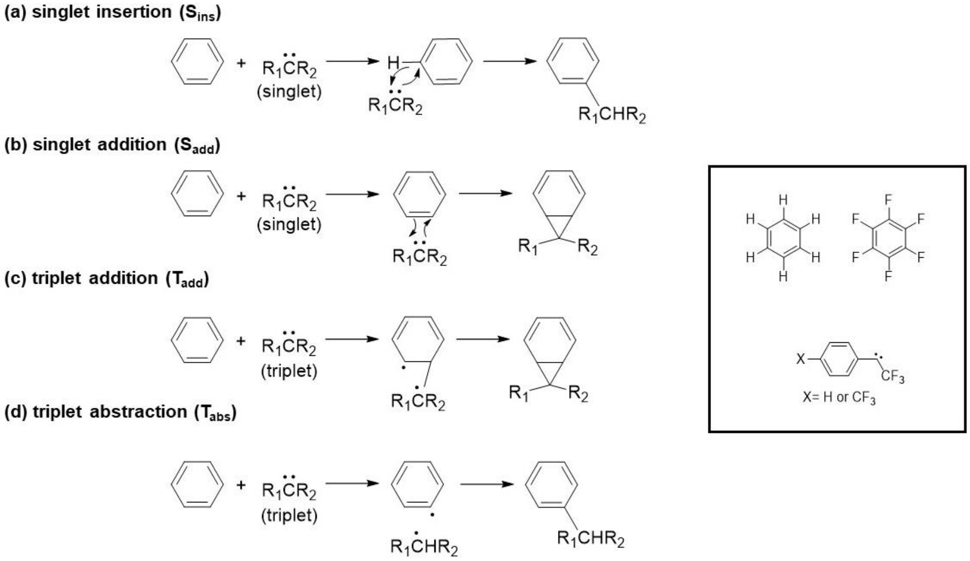

Figure S62. Possible reactions between carbene and benzene, and model carbene with benzene derivatives used for the study (shown in the square).

For comparative purposes, we performed analogous calculations on benzene (see Table S4). The singlet C–H insertion has a higher barrier than the addition pathways. The barriers to atom abstraction pathways are the highest amongst the four reactions. Barriers to carbene addition to the ring moiety are found to be quite similar to those in C_6F_6.

Our calculations further indicate that the model carbene used for our calculations adopts a triplet ground state electronic configuration, which means that the mechanism for the reaction between our carbene and the ring species will be dominated by the T_{add} pathway shown in Figure S62(c). The data in the Table show that fluorinating the aromatic rings does not significantly change the T_{add} barrier heights, suggesting that this strategy will not avoid cyclopropanation.

Table S4. Calculated[a] enthalpy and free-energy reaction barriers (kJ/mol) of model carbene to hexafluorobenzene or benzene[b]

benzene derivative	*para* substituent	reaction barriers	S_{ins}	S_{add}	T_{add}	T_{abs}
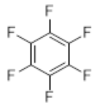	X = H	ΔH_{298K}	n/a	–32.8	10.5	167.1
		ΔG_{298K}	n/a	27.8	66.4	211.6
	X = CF_3	ΔH_{298K}	n/a	–34.9	11.2	168.3
		ΔG_{298K}	n/a	25.2	68.0	213.1
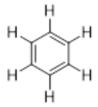	X = H	ΔH_{298K}	6.0	–33.7	3.6	26.6
		ΔG_{298K}	59.9	22.7	55.9	73.1
	X = CF_3	ΔH_{298K}	-2.5	–41.2	0.2	25.2
		ΔG_{298K}	52.3	16.0	54.1	71.8

[a] The geometry optimization and frequency calculations were performed using M06-2X[5]-D3[6]/6-31G(d,p) with the Gaussian 16[7] package.

[b] Calculations were carried out at standard conditions of 298 K and 1 atm pressure.

Coordinates of optimized structure.

The unit is in Å, and the format is shown below:

Name; E_{el}; H_{298K}; G_{298K}

charge multiplicity

cartesian coordinate

model carbene (singlet) X=H; E_{el}= -607.0358015; H_{298K}= -606.915700; G_{298K}= -606.961020

0 1

C	1.59501	1.37617	-0.00008
C	2.90960	0.93500	0.00004
C	3.16196	-0.43569	0.00008
C	2.11462	-1.36481	0.00001
C	0.80392	-0.92646	-0.00010
C	0.50980	0.46613	-0.00013
C	-0.78159	1.08531	-0.00017
C	-1.91775	0.09079	-0.00001
F	-1.88485	-0.70556	-1.09196
F	-3.11606	0.68083	-0.00007
F	-1.88474	-0.70512	1.09227
H	3.73158	1.64213	0.00006
H	1.34742	2.43245	-0.00016
H	-0.00582	-1.64644	-0.00018
H	2.33625	-2.42651	0.00004
H	4.18815	-0.79166	0.00016

model carbene (singlet) X=CF_3; E_{el}= -943.9696441; H_{298K}= -943.840751; G_{298K}= -943.896138

0 1

C	-0.06613	1.69998	-0.00208
C	1.30072	1.45340	-0.00984
C	1.73558	0.13337	-0.01577
C	0.83648	-0.94019	-0.01525
C	-0.52123	-0.69117	-0.00742
C	-1.00693	0.64604	-0.00061
C	-2.37777	1.07988	0.00659
C	-3.36097	-0.06695	0.00248
F	-3.21140	-0.84197	-1.09391
F	-4.62920	0.34588	0.00683
F	-3.20692	-0.85205	1.09106
H	2.02086	2.26280	-0.01368
H	-0.45864	2.71103	0.00214
H	-1.21929	-1.51947	-0.00781
H	1.21666	-1.95637	-0.02486
C	3.21007	-0.18702	0.00086
F	3.96361	0.90317	-0.16957
F	3.55810	-0.74835	1.16532
F	3.51930	-1.05802	-0.96745

model carbene (triplet) X=H; E_{el}= -607.0469848; H_{298K}= -606.927070; G_{298K}= -606.974017

0 3

C	1.64127	1.37611	0.00001
C	2.96339	0.96564	-0.00002
C	3.27963	-0.39439	-0.00002
C	2.25900	-1.34696	0.00000
C	0.93095	-0.95473	0.00003
C	0.59742	0.42268	0.00002
C	-0.74857	0.83507	0.00008
C	-2.00940	0.07188	0.00000
F	-2.10824	-0.72952	-1.07800
F	-3.07080	0.88370	0.00016
F	-2.10816	-0.72982	1.07777
H	3.75586	1.70664	-0.00004
H	1.38831	2.43123	0.00002
H	0.13720	-1.69430	0.00005
H	2.50405	-2.40397	0.00001
H	4.31718	-0.71064	-0.00003

model carbene (triplet) X=CF_3; E_{el}= -943.9841186; H_{298K}= -943.855318; G_{298K}= -943.912560

0 3

C	-0.01393	1.65946	-0.00735
C	1.34538	1.40777	-0.02399
C	1.80835	0.09090	-0.03840
C	0.91198	-0.97860	-0.03812
C	-0.45031	-0.74013	-0.02178
C	-0.93928	0.59013	-0.00466
C	-2.32328	0.84502	0.01068
C	-3.48748	-0.06272	0.00737
F	-3.50540	-0.83799	-1.09209
F	-4.63531	0.61666	0.04773
F	-3.46474	-0.89728	1.06292
H	2.05578	2.22714	-0.03405
H	-0.38596	2.67819	0.00006
H	-1.15152	-1.56742	-0.02499
H	1.28893	-1.99528	-0.05845
C	3.28575	-0.17586	0.00390
F	3.97941	0.79089	-0.61338
F	3.73862	-0.23837	1.26538
F	3.59516	-1.34041	-0.58261

Transition state of singlet insertion to benzene with X=H; E_{el}= -839.1781227; H_{298K}= -838.950859; G_{298K}= -839.008414

0 1

C	0.13502	0.60266	-0.59495
C	-2.71379	0.50655	0.02842
C	-3.81707	-0.28469	-0.24635
C	-3.75047	-1.66945	-0.07767
C	-2.57784	-2.26291	0.38928
C	-1.46707	-1.47934	0.66517
C	-1.51189	-0.09122	0.45263
H	-2.75655	1.58003	-0.10454
H	-4.73639	0.17492	-0.59355
H	-4.61624	-2.28423	-0.30184
H	-2.53513	-3.33616	0.54246
H	-0.55080	-1.93487	1.02692
H	-0.77981	0.51222	1.01694
C	1.28074	-0.25016	-0.25092
C	1.32657	-1.49994	-0.90250
C	2.37406	0.11019	0.56225
C	2.40292	-2.35827	-0.74577
H	0.48572	-1.76390	-1.53769
C	3.46584	-0.73984	0.70041
H	2.37540	1.06513	1.07427
C	3.47912	-1.97456	0.05678
H	2.41805	-3.31523	-1.25686
H	4.30663	-0.44110	1.31785
H	4.33068	-2.63748	0.17486
C	0.26773	2.03414	-0.14971
F	-0.83015	2.76238	-0.42532
F	0.50554	2.27614	1.17658
F	1.29075	2.57500	-0.82696

Transition state of singlet insertion to benzene with X=CF_3; E_{el}= -1176.1153446; H_{298K}= -1175.879127; G_{298K}= -1175.946432

0 1

C	1.42037	-0.73970	-0.57300
C	3.84459	0.66267	-0.06145
C	4.34668	1.90547	-0.39903
C	3.60438	3.05936	-0.12684
C	2.36744	2.97026	0.51230
C	1.85930	1.72738	0.85688
C	2.56482	0.55330	0.52798
H	4.40291	-0.23942	-0.27870
H	5.31614	1.98654	-0.87851
H	3.99931	4.03174	-0.40356
H	1.81033	3.87072	0.74783
H	0.89866	1.64800	1.35588
H	2.32777	-0.33062	1.13403
C	0.00577	-0.47469	-0.27543
C	-0.54395	0.69216	-0.85361
C	-0.86819	-1.35099	0.39367
C	-1.88986	0.98132	-0.75514
H	0.13067	1.35607	-1.38619
C	-2.23257	-1.07983	0.46959
H	-0.48816	-2.25970	0.84356
C	-2.73527	0.08523	-0.09126
H	-2.30222	1.87798	-1.20657
H	-2.90223	-1.76839	0.97212
C	1.91027	-2.08439	-0.11321
F	3.23541	-2.23459	-0.29721
F	1.71380	-2.42572	1.19795
F	1.28871	-3.01818	-0.84710
C	-4.19384	0.42834	0.01878
F	-4.91879	-0.58671	0.50433
F	-4.38381	1.48175	0.82932
F	-4.70786	0.76253	-1.17411

Transition state of singlet addition to benzene with X=H; E_{el}= -839.1939857; H_{298K}= -838.965972; G_{298K}= -839.022574

0 1

C	-1.56316	-0.53414	-1.31923
C	-2.86253	-0.94019	-1.04604
C	-3.13483	-1.59508	0.15279
C	-2.11386	-1.83647	1.07384
C	-0.81867	-1.42058	0.80354
C	-0.50736	-0.76964	-0.41380
C	0.79917	-0.35107	-0.88464
C	2.00614	-0.88769	-0.17136
H	-1.31458	-0.03512	-2.25065
H	-0.03394	-1.60564	1.52602
F	2.20952	-2.11344	-0.68724
F	2.01555	-1.05436	1.18472
F	3.10288	-0.14632	-0.42071
H	-2.33498	-2.34887	2.00440
H	1.88883	1.93528	-1.19952
C	0.99736	1.93512	-0.58544
C	-0.21808	2.43089	-1.10161
C	-1.33432	2.49341	-0.29527
C	-1.25450	2.11385	1.05801
C	-0.04242	1.72221	1.59940
C	1.09185	1.64455	0.78426
H	-0.27665	2.71614	-2.14691
H	-2.28117	2.83682	-0.70013
H	-2.14252	2.15332	1.68073
H	0.03071	1.46404	2.65081
H	2.04756	1.35970	1.20694
H	-3.65682	-0.75772	-1.76206
H	-4.14679	-1.92187	0.37276

Transition state of singlet addition to benzene with X=CF_3; E_{el}= -1176.1307694; H_{298K}= -1175.893891; G_{298K}= -1175.960250

0 1

C	-0.41046	0.11737	-1.48474
C	-1.78674	0.18380	-1.30939
C	-2.34567	-0.39160	-0.17358
C	-1.54939	-1.02819	0.77992
C	-0.17617	-1.07818	0.60486
C	0.42761	-0.51192	-0.54290
C	1.83410	-0.53543	-0.91000
C	2.74380	-1.43676	-0.12434
H	0.06125	0.53968	-2.36577
H	0.43848	-1.56626	1.34976
F	2.60002	-2.65151	-0.68341
F	2.56996	-1.62314	1.21655
F	4.03191	-1.06999	-0.24595
H	-2.01292	-1.48038	1.65023
H	3.62943	1.33701	-0.95520
C	2.72987	1.61341	-0.41955
C	1.79168	2.48054	-1.01140
C	0.67232	2.87128	-0.30448
C	0.48515	2.44016	1.02113
C	1.44553	1.65667	1.63978
C	2.57730	1.24978	0.92595
H	1.93668	2.80523	-2.03656
H	-0.06931	3.51392	-0.76858
H	-0.40512	2.74154	1.56400
H	1.31941	1.34912	2.67270
H	3.34102	0.65286	1.40983
H	-2.42355	0.66078	-2.04571
C	-3.82636	-0.28314	0.07291
F	-4.30949	-1.38482	0.66168
F	-4.10321	0.74967	0.88403
F	-4.51038	-0.09133	-1.06176

Transition state of triplet addition to benzene with X=H; E_{el}= -839.1901998; H_{298K}= -838.963124; G_{298K}= -839.022929

0 3

C	0.57908	-1.68084	-0.49981
C	1.41702	-2.78484	-0.49288
C	2.75565	-2.65255	-0.12203
C	3.25490	-1.40032	0.23838
C	2.42978	-0.28671	0.23231
C	1.06941	-0.40750	-0.13756
C	0.19760	0.71547	-0.13598
C	0.59766	2.13567	-0.07826
H	-0.45928	-1.77365	-0.80051
H	2.82183	0.68383	0.51712
F	1.66711	2.41463	-0.84431
F	0.91306	2.52266	1.17792
F	-0.39985	2.94395	-0.48579
H	4.29578	-1.29352	0.52636
H	-1.57774	1.57035	1.06285
C	-1.78642	0.60908	0.60129
C	-2.49913	0.58570	-0.63011
C	-3.12029	-0.57515	-1.05577
C	-3.12023	-1.71611	-0.24480
C	-2.49430	-1.68301	1.00646
C	-1.85743	-0.53395	1.44030
H	-2.51504	1.48135	-1.24225
H	-3.63151	-0.59315	-2.01295
H	-3.62342	-2.61816	-0.57668
H	-2.52100	-2.55912	1.64661
H	-1.38743	-0.50307	2.41778
H	1.02755	-3.75579	-0.78165
H	3.40752	-3.51985	-0.11652

Transition state of triplet addition to benzene with X=CF_3; E_{el}= -1176.1287192; H_{298K}= -1175.892682; G_{298K}= -1175.962180

0 3

C	-0.39389	0.75296	-0.45767
C	-1.75897	0.97920	-0.41965
C	-2.62224	-0.04382	-0.02831
C	-2.12199	-1.29898	0.31984
C	-0.75908	-1.53557	0.27997
C	0.13439	-0.50941	-0.10992
C	1.53994	-0.71863	-0.13635
C	2.22891	-2.02583	-0.09548
H	0.28719	1.53741	-0.76922
H	-0.36938	-2.50867	0.55756
F	1.62757	-2.96008	-0.85167
F	2.28863	-2.52235	1.15905
F	3.49871	-1.92284	-0.52853
H	-2.80508	-2.08273	0.62936
H	3.43703	-0.11402	1.02831
C	2.91318	0.72652	0.58106
C	3.38621	1.24142	-0.65845
C	3.02162	2.51057	-1.07177
C	2.24786	3.32994	-0.24118
C	1.84463	2.86811	1.01704
C	2.18974	1.59630	1.43884
H	4.00596	0.60913	-1.28557
H	3.35880	2.88201	-2.03406
H	1.97966	4.33049	-0.56351
H	1.27127	3.51656	1.67175
H	1.89364	1.24501	2.42182
H	-2.16246	1.94950	-0.68790
C	-4.10532	0.18413	-0.03870
F	-4.72385	-0.53443	0.91012
F	-4.41196	1.47416	0.16316
F	-4.65316	-0.16850	-1.21259

Transition state of triplet abstraction to benzene with X=H; E_{el}= -839.1759966; H_{298K}= -838.954365; G_{298K}= -839.016385

0 3

C	-1.29333	-1.76299	-0.26104
C	-2.30288	-2.70833	-0.31547
C	-3.63762	-2.32042	-0.18179
C	-3.95441	-0.97332	0.00383
C	-2.95552	-0.01579	0.05843
C	-1.59591	-0.39395	-0.06935
C	-0.54613	0.55506	-0.00911
C	-0.70825	2.02043	0.12076
H	-0.25430	-2.05746	-0.37782
H	-3.20745	1.02879	0.20463
F	-1.25701	2.37598	1.29860
F	-1.50596	2.53566	-0.83559
F	0.47664	2.64499	0.02840
H	-4.99134	-0.67039	0.10658
H	0.67948	0.14746	0.04695
C	1.97459	-0.24555	0.03591
C	2.34906	-1.37037	0.74886
C	3.69280	-1.74959	0.70997
C	4.60809	-1.00224	-0.02814
C	4.19587	0.12952	-0.73000
C	2.85701	0.52280	-0.70149
H	1.62539	-1.93803	1.32678
H	4.02211	-2.62669	1.25846
H	5.65067	-1.30186	-0.05450
H	4.91662	0.70878	-1.29884
H	2.51624	1.40522	-1.23349
H	-2.05370	-3.75367	-0.46559
H	-4.42684	-3.06342	-0.22401

Transition state of triplet abstraction to benzene with X=CF_3; E_{el}= -1176.1136518; H_{298K}= -1175.883146; G_{298K}= -1175.955443

0 3

C	0.63847	-0.82355	0.15176
C	1.94379	-1.27808	0.15856
C	2.99451	-0.37068	0.01156
C	2.73890	0.99272	-0.14067
C	1.43595	1.45733	-0.14690
C	0.35362	0.55432	-0.00231
C	-0.99311	0.99299	-0.01240
C	-1.44319	2.40250	-0.09288
H	-0.18597	-1.51937	0.27461
H	1.23758	2.51581	-0.27088
F	-1.12438	2.97390	-1.26924
F	-0.88715	3.16978	0.86399
F	-2.77394	2.48880	0.04834
H	3.56636	1.68328	-0.26186
H	-1.95119	0.11906	-0.05962
C	-2.96713	-0.76647	-0.03696
C	-2.87660	-1.92192	-0.79204
C	-3.94535	-2.81958	-0.73782
C	-5.05495	-2.53704	0.05589
C	-5.11341	-1.35900	0.79892
C	-4.05480	-0.45035	0.75645
H	-2.00919	-2.12421	-1.41376
H	-3.90954	-3.73635	-1.31803
H	-5.88186	-3.23854	0.09355
H	-5.98393	-1.14467	1.41101
H	-4.08318	0.47683	1.31980
H	2.15821	-2.33517	0.27216
C	4.41155	-0.86187	0.07863
F	5.24392	-0.07317	-0.61533
F	4.86311	-0.89374	1.34212
F	4.52658	-2.10607	-0.40830

Transition state of singlet addition to hexafluorobenzene with X=H; E_{el}= -1434.3694436; H_{298K}= -1434.184692;
G_{298K}= -1434.252730

0 1

C	1.46509	1.12942	-1.45331
C	2.72914	1.62373	-1.17536
C	2.93588	2.34715	-0.00001
C	1.88367	2.57701	0.88698
C	0.61864	2.07716	0.61174
C	0.37717	1.33895	-0.57295
C	-0.87909	0.77005	-1.00902
C	-2.11416	1.26059	-0.29705
H	1.27306	0.55739	-2.35697
H	-0.19037	2.25537	1.30924
F	-2.31947	2.51659	-0.72987
F	-2.10429	1.31692	1.06427
F	-3.19070	0.53293	-0.61168
H	2.05558	3.14340	1.79588
C	-0.87132	-1.43502	-0.71088
C	0.45843	-1.71651	-1.09103
C	1.48362	-1.57399	-0.19024
C	1.20955	-1.26861	1.15244
C	-0.09802	-1.12609	1.57417
C	-1.14028	-1.28230	0.66048
H	3.54995	1.44893	-1.86208
F	-1.85754	-1.76280	-1.53152
F	-2.38949	-1.27367	1.09031
F	-0.37319	-0.89108	2.85007
F	2.21403	-1.11924	2.00339
F	2.74688	-1.68480	-0.58350
F	0.72241	-1.90921	-2.37688
H	3.92420	2.73492	0.22697

Transition state of singlet addition to hexafluorobenzene with X=CF_3; E_{el}= -1771.3040462; H_{298K}= -1771.110539;
G_{298K}= -1771.188858

0 1

C	0.86117	0.22126	-1.58233
C	2.22130	0.08201	-1.36806
C	2.78623	0.65686	-0.22874
C	2.01050	1.36714	0.68332
C	0.64515	1.50012	0.46571
C	0.03732	0.92946	-0.67627
C	-1.36327	0.99374	-1.05143
C	-2.19933	2.00833	-0.31288
H	0.38543	-0.21684	-2.45504
H	0.04538	2.05040	1.17947
F	-1.83144	3.20683	-0.79694
F	-2.08781	2.08515	1.04233
F	-3.50299	1.84348	-0.55188
H	2.47689	1.81110	1.55516
C	-2.35647	-0.98823	-0.61216
C	-1.31516	-1.84183	-1.03037
C	-0.28713	-2.15514	-0.17523
C	-0.31843	-1.71920	1.15829
C	-1.39254	-0.98138	1.61898
C	-2.44352	-0.67582	0.75443
H	2.84624	-0.45933	-2.07001
C	4.25942	0.45738	0.02056
F	-3.42321	-0.84436	-1.38102
F	-3.52303	-0.07963	1.22615
F	-1.45423	-0.60117	2.88737
F	0.69123	-2.01347	1.96244
F	0.76635	-2.83480	-0.61032
F	-1.23988	-2.17175	-2.31220
F	4.73213	1.31179	0.93420
F	4.96595	0.62446	-1.10448
F	4.50411	-0.78360	0.46075

Transition state of triplet addition to hexafluorobenzene with X=H; E_{el}= -1434.3633799; H_{298K}= -1434.179559;
G_{298K}= -1434.251034

0 3

C	-0.76125	1.72116	-1.01124
C	-1.24423	3.01605	-1.12133
C	-2.53307	3.32212	-0.68483
C	-3.34086	2.32168	-0.14327
C	-2.86777	1.02430	-0.02362
C	-1.56150	0.70222	-0.45533
C	-1.05573	-0.62382	-0.31696
C	-1.87499	-1.85995	-0.22872
H	0.22703	1.46992	-1.38371
H	-3.49169	0.24955	0.40885
F	-2.83913	-1.86862	-1.16628
F	-2.49791	-1.97043	0.96056
F	-1.13545	-2.95964	-0.40136
H	-4.34519	2.55768	0.19249
C	0.72911	-0.92547	0.83695
C	1.64210	-1.39454	-0.14037
C	2.56797	-0.53779	-0.70582
C	2.71459	0.75599	-0.20933
C	1.89217	1.20805	0.82561
C	0.96237	0.35951	1.38526
H	-0.61908	3.78833	-1.55687
F	0.13735	-1.83241	1.62958
F	0.17334	0.77619	2.36372
F	2.03149	2.44870	1.27634
F	3.61310	1.57272	-0.74168
F	3.36541	-0.96163	-1.67950
F	1.54339	-2.64252	-0.56709
H	-2.90891	4.33596	-0.77142

Transition state of triplet addition to hexafluorobenzene with X=CF_3; E_{el}= -1771.3002128; H_{298K}= -1771.107571;
G_{298K}= -1771.188975

0 3

C	0.95024	-0.36976	-0.79735
C	2.27313	-0.77867	-0.78744
C	3.25517	0.07490	-0.28701
C	2.91944	1.34025	0.19379
C	1.59819	1.75409	0.19000
C	0.58645	0.89977	-0.30313
C	-0.78056	1.30605	-0.29206
C	-1.26283	2.71264	-0.27786
H	0.18420	-1.01012	-1.22227
H	1.33189	2.73393	0.57037
F	-0.59797	3.45720	-1.17833
F	-1.07191	3.29705	0.91968
F	-2.56381	2.79283	-0.56746
H	3.69733	1.99738	0.56690
C	-2.20488	0.10265	0.76919
C	-3.04717	-0.35815	-0.27390
C	-2.88637	-1.62731	-0.79730
C	-1.99732	-2.51970	-0.20015
C	-1.22829	-2.11899	0.89573
C	-1.37950	-0.85155	1.41391
H	2.55244	-1.74962	-1.18133
C	4.68216	-0.39084	-0.22185
F	-2.62587	1.14403	1.50244
F	-0.65345	-0.45492	2.44734
F	-0.37271	-2.97383	1.44069
F	-1.85396	-3.74079	-0.69345
F	-3.62171	-2.02440	-1.82840
F	-3.93403	0.47057	-0.79739
F	5.54068	0.63229	-0.32881
F	4.94531	-1.00537	0.94101
F	4.96133	-1.26397	-1.19964

Transition state of triplet abstraction to hexafluorobenzene with X=H; E_{el}= -1434.3023866; H_{298K}= -1434.119931;
G_{298K}= -1434.195711

0 3

C	2.40952	1.79975	-0.03747
C	3.31425	2.82162	0.19261
C	4.66749	2.53927	0.38896
C	5.11638	1.21795	0.35171
C	4.22938	0.18088	0.11850
C	2.85332	0.45744	-0.07656
C	1.94282	-0.58796	-0.32377
C	2.07892	-2.05797	-0.21034
H	1.35652	2.01043	-0.18379
H	4.57954	-0.84452	0.08351
F	2.25028	-2.45060	1.06241
F	3.15260	-2.49433	-0.89776
F	1.00429	-2.68473	-0.68877
H	6.16744	0.99708	0.50497
C	-1.32709	0.05860	-0.03093
C	-1.82343	1.32863	-0.18308
C	-3.20036	1.52160	-0.12971
C	-4.03552	0.42676	0.06664
C	-3.50867	-0.85242	0.21405
C	-2.13032	-1.03914	0.15993
F	0.31113	-0.19815	-0.12156
H	2.96441	3.84806	0.22297
F	-1.62417	-2.26008	0.30011
F	-1.02130	2.37957	-0.37352
F	-3.72377	2.73592	-0.26685
F	-5.35050	0.60509	0.11708
F	-4.32718	-1.88233	0.40200
H	5.36968	3.34578	0.57087

Transition state of triplet abstraction to hexafluorobenzene with X=CF_3; E_{el}= -1771.2390785; H_{298K}= -1771.047721;
G_{298K}= -1771.133703

0 3

C	-1.75146	-0.63021	-0.21337
C	-2.94017	-1.32246	-0.07210
C	-4.13836	-0.62612	0.09073
C	-4.15338	0.76938	0.11288
C	-2.97419	1.47642	-0.03199
C	-1.74829	0.78399	-0.19556
C	-0.54249	1.49383	-0.35071
C	-0.21859	2.93059	-0.18396
H	-0.81323	-1.15926	-0.33081
H	-2.98136	2.56008	-0.01840
F	-0.32224	3.31839	1.09644
F	-1.06891	3.69697	-0.89310
F	1.01899	3.19908	-0.59560
H	-5.09208	1.29496	0.25023
C	2.33716	-0.17673	-0.04400
C	2.39857	-1.53461	-0.23013
C	3.63667	-2.16513	-0.15520
C	4.77452	-1.40557	0.09699
C	4.68604	-0.02876	0.27811
C	3.44490	0.59709	0.20244
F	0.86802	0.59838	-0.15451
H	-2.94497	-2.40676	-0.07683
C	-5.43429	-1.38007	0.18754
F	3.35577	1.91163	0.37473
F	1.30457	-2.26108	-0.47388
F	3.74258	-3.47880	-0.32437
F	5.95828	-2.00179	0.16856
F	5.78718	0.67412	0.51875
F	-6.00129	-1.53621	-1.01808
F	-5.25729	-2.60444	0.70169
F	-6.32214	-0.73612	0.95779

hexafluorobenzene; E_{el}= -827.3198402; H_{298K}= -827.256504; G_{298K}= -827.302300

0 1

C	-1.11550	0.82801	0.00000
C	0.15932	1.38015	-0.00001
C	1.27480	0.55208	-0.00001
C	1.11550	-0.82801	-0.00000
C	-0.15932	-1.38015	0.00000
C	-1.27480	-0.55208	0.00001
F	-2.18177	1.61892	0.00000
F	0.31147	2.69897	-0.00001
F	2.49311	1.07955	-0.00001
F	2.18177	-1.61892	0.00000
F	-0.31147	-2.69897	0.00001
F	-2.49311	-1.07955	0.00001

benzene; E_{el}= -232.1443011; H_{298K}= -232.037442; G_{298K}= -232.070212

0 1

C	0.20704	-1.37731	0.00000
C	1.29633	-0.50935	-0.00002
C	1.08929	0.86795	0.00001
C	-0.20703	1.37731	-0.00000
C	-1.29633	0.50935	-0.00001
C	-1.08929	-0.86794	0.00001
H	0.36836	-2.45018	-0.00000
H	2.30612	-0.90613	0.00001
H	1.93780	1.54406	0.00003
H	-0.36835	2.45017	0.00002
H	-2.30611	0.90612	-0.00003
H	-1.93781	-1.54406	0.00002

References

1 F. Ceretta, A. Zaggia, L. Conte and B. Ameduri, *J. Fluorine Chem.*, 2012, **135**, 220–224.

2 M. L. Lepage, C. Simhadri, C. Liu, M. Takaffoli, L. Bi, B. Crawford, A. S. Milani and J. E. Wulff, *Science*, 2019, **366**, 875–878.

3 (*a*) T. Yoshida, Y. Wada and N. Foster, "Experimental evaluation of fire and explosion hazards of reactive chemical substances" in Safety of Reactive Chemicals and Pyrotechnics, T. Yoshida, Y. Wada and N. Foster, Eds. (Elsevier, vol. 5, 1995), pp. 75–253; (*b*) T. Yoshida, F. Yoshizawa, M. Itoh, T. Matsunaga and M. Watanabe, Prediction of Fire and Explosion Hazard for Reactive Chemicals (I): Estimation of Explosive Properties of Self-Reactive Chemicals from SC-DSC Data, *Kogyo Kayak*, 1987, **48**, 311–316.

4 S. Ebnesajjad, C. Ebnesajjad, "Theories of Adhesion" in Surface Treatment of Materials for Adhesive Bonding (Elsevier, ed. 2, 2013), pp. 77–91.

5 Y. Zhao, D. G. Truhlar, *Theor. Chem. Acc.*, 2008, **120**, 215–241.

6 S. Grimme, J. Antony, S. Ehrlich, H. Krieg, *J. Chem. Phys.* **2010**, *132*, 154104.

7 Gaussian 16, Revision C.01, M. J. Frisch, G. W. Trucks, H. B. Schlegel, G. E. Scuseria, M. A. Robb, J. R. Cheeseman, G. Scalmani, V. Barone, G. A. Petersson, H. Nakatsuji, X. Li, M. Caricato, A. V. Marenich, J. Bloino, B. G. Janesko, R. Gomperts, B. Mennucci, H. P. Hratchian, J. V. Ortiz, A. F. Izmaylov, J. L. Sonnenberg, D. Williams-Young, F. Ding, F. Lipparini, F. Egidi, J. Goings, B. Peng, A. Petrone, T. Henderson, D. Ranasinghe, V. G. Zakrzewski, J. Gao, N. Rega, G. Zheng, W. Liang, M. Hada, M. Ehara, K. Toyota, R. Fukuda, J. Hasegawa, M. Ishida, T. Nakajima, Y. Honda, O. Kitao, H. Nakai, T. Vreven, K. Throssell, J. A. Montgomery, Jr., J. E. Peralta, F. Ogliaro, M. J. Bearpark, J. J. Heyd, E. N. Brothers, K. N. Kudin, V. N. Staroverov, T. A. Keith, R. Kobayashi, J. Normand, K. Raghavachari, A. P. Rendell, J. C. Burant, S. S. Iyengar, J. Tomasi, M. Cossi, J. M. Millam, M. Klene, C. Adamo, R. Cammi, J. W. Ochterski, R. L. Martin, K. Morokuma, O. Farkas, J. B. Foresman, and D. J. Fox, Gaussian, Inc., Wallingford CT, 2016.
